# Local conservation scores without a priori assumptions on neutral substitution rates

**DOI:** 10.1186/1471-2105-9-190

**Published:** 2008-04-11

**Authors:** Janis Dingel, Pavol Hanus, Niccolò Leonardi, Joachim Hagenauer, Jürgen Zech, Jakob C Mueller

**Affiliations:** 1 Institute for Communications Engineering, Technische Universität München, Munich, Germany; 2 MRC Clinical Sciences Centre, Imperial College, London, UK; 3 Max-Planck Institute for Ornithology, Starnberg-Seewiesen, Germany

## Abstract

**Background:**

Comparative genomics aims to detect signals of evolutionary conservation as an indicator of functional constraint. Surprisingly, results of the ENCODE project revealed that about half of the experimentally verified functional elements found in non-coding DNA were classified as unconstrained by computational predictions. Following this observation, it has been hypothesized that this may be partly explained by biased estimates on neutral evolutionary rates used by existing sequence conservation metrics. All methods we are aware of rely on a comparison with the neutral rate and conservation is estimated by measuring the deviation of a particular genomic region from this rate. Consequently, it is a reasonable assumption that inaccurate neutral rate estimates may lead to biased conservation and constraint estimates.

**Results:**

We propose a conservation signal that is produced by local Maximum Likelihood estimation of evolutionary parameters using an optimized sliding window and present a Kullback-Leibler projection that allows multiple different estimated parameters to be transformed into a conservation measure. This conservation measure does not rely on assumptions about neutral evolutionary substitution rates and little a priori assumptions on the properties of the conserved regions are imposed. We show the accuracy of our approach (KuLCons) on synthetic data and compare it to the scores generated by state-of-the-art methods (phastCons, GERP, SCONE) in an ENCODE region. We find that KuLCons is most often in agreement with the conservation/constraint signatures detected by GERP and SCONE while qualitatively very different patterns from phastCons are observed. Opposed to standard methods KuLCons can be extended to more complex evolutionary models, e.g. taking insertion and deletion events into account and corresponding results show that scores obtained under this model can diverge significantly from scores using the simpler model.

**Conclusion:**

Our results suggest that discriminating among the different degrees of conservation is possible without making assumptions about neutral rates. We find, however, that it cannot be expected to discover considerably different constraint regions than GERP and SCONE. Consequently, we conclude that the reported discrepancies between experimentally verified functional and computationally identified constraint elements are likely not to be explained by biased neutral rate estimates.

## Background

Joint analysis of DNA orthologues from multiple species conveys important information about sequence properties. This comparative approach is a powerful concept in genome analysis today. DNA sequences with unexpected conservation across species have gained particular interest [1-3] as they are likely to encode important and constrained functionality across species. Throughout the paper the term *conserved *will refer to *primary sequence conservation *among multiple species. There are many types of conservation acting at different constraint levels upon the genome. Secondary and tertiary structures as well as interactions of non-coding RNA may be preserved with little primary sequence information remaining conserved [4].

The problem of measuring the conservation of sequences across multiple species has been addressed in a number of publications, [5-10]. Stojanovic et. al. compared 5 different methods for scoring the conservation of a multiple sequence alignment in gene regulatory regions [5]. Blanchette et. al. developed an exact algorithm, limited to short multiple sequences, for the detection of conserved motifs based on a parsimony approach [6]. Margulies et al. presented two alignment based methods that incorporate phylogenetic information and are suitable for whole genome analysis [7]. Siepel and Haussler presented an approach (phastCons) using a phylogenetic Hidden Markov Model (phylo-HMM) allowing for high throughput measurement of evolutionary constraint [8]. Cooper et al. introduced GERP and more recently Asthana et al. presented SCONE which produce per-base scores of conservation and constraint.

PhastCons, GERP and SCONE scores have been used as comparisons in this paper and are briefly reviewed in the *Discussion*. These methods require the a priori estimation of a neutral evolutionary rate and measure conservation as the "surprise" of observing the analyzed data assuming the neutral model. Neutral substitution rates are usually estimated from fourfold degenerated sites or ancestral repeats [11,12].

The ENCODE project revealed that about half of the analyzed functional elements found in non-coding DNA had been classified as unconstrained [13,14]. Pheasant and Mattick [15], among others, have argued that this could partly be explained by questioning the neutral rate of evolution used by existing sequence conservation studies. Wrong assumptions about the neutral rate would lead to biased conservation measures and eventually to an over- or underestimate of the fraction of the genome under evolutionary constraint. For example, ancestral repeats are often assumed to evolve neutrally, but have been previously shown to include a nontrivial amount of constrained DNA [9,16]. Here, we propose a method that tries to avoid such a priori assumptions. We suggest that the Maximum Likelihood (ML) estimate of rate heterogeneity is a more direct measure for sequence conservation. Different estimators for these rates have been presented and reviewed in the literature [17-20]. Here, we obtain the ML estimate of the rate process using an optimized window function. While this approach does not require assumptions about neutral rates, prior distribution of rates or transition probabilities between rate categories, we show *in silico *that reliable estimation in the mean squared error (MSE) sense is achieved in regions of conserved sequence. We present a qualitative comparison of the scores calculated by KuLCons and the established methods phastCons, GERP and SCONE that assume a neutral model. ENCODE regions were used for comparison.

Furthermore, we present an information theoretic projection of local multiple parameter estimates to a score which allows for richer or more complex parameter models like the consideration of insertion and deletion (InDel) rates. Results taking gaps in the alignment as InDels into account are presented.

### Probabilistic modeling in phylogenetics

We will summarize the basic concepts of mathematical phylogenetic modeling in order to introduce the notation. A more thorough introduction can be found for example in [21-23]. Throughout, we assume a given multiple sequence alignment ***A ***∈ {*A*, *C*, *G*, *T*, -}^*n *× *l *^of length *l *comprising the orthologous sequences of *n *species. We denote ***a***_*i *_as the *i*th column of ***A***. An evolutionary model is commonly described by a set of parameters ***ψ ***that imposes a probabilistic model on how a base of a common ancestor evolves along a phylogenetic tree. The realizations of this process are the columns of the multiple sequence alignments. A single column ***a ***of such an alignment follows the distribution *p*(***a***; ***ψ***). Different sites evolve differently and, hence, each column ***a***_*i *_could be associated with a different model ***ψ***_*i*_. Most often, ***ψ ***= {T, *λ*(*e*), ***R*, *π***, *θ*} comprises at least the following parameters: T = {*V*, *E*} denotes the topology of the binary phylogenetic tree relating the *n *species with nodes *V *and branches *E *⊂ {(*u*, *v*) : *u*, *v *∈ *V*, *u *≠ *v*}. It is often useful to distinguish between the set of inner nodes *I *⊂ *V *and the set of leaves *Q *= {*q*_1_, ..., *q*_*n*_} = *V*\*I*.

Furthermore, a map $\lambda :E\to {\mathbb{R}}_{+}$, *e *↦ *λ*(*e*) assigns positive branchlengths to *E*. The time continuous substitution process between two nodes is assumed to satisfy the Markov property and to be identical for all branches with discrete state space A = {*A, C, G, T*}. Such a process is specified by a rate matrix ***R ***and a stationary distribution ***π ***= [*π*_*A*_, ..., *π*_*T*_]. The transition probability matrix between two nodes connected by branch *e *is then given by ***P***_*e *_= *e*^*λ*(*e*)***R ***^[22]. Reversibility is an additional constraint, often assumed when modeling DNA sequences. In a time reversible process, the amount of substitutions from *μ *∈ A to *ν *∈ A is equal to the amount of substitutions from *ν *to *μ*, i.e. *π*_*μ *_*R*_*μν *_= *π*_*ν *_*R*_*νμ*_. The parameters presented so far model the evolution of sequences along a phylogenetic tree (time-process). However, different sites in the genome are subject to different evolutionary processes, e.g. due to selection pressures resulting in varying substitution rates (space-process). This characteristic of evolution over sites, often called rate heterogeneity, is commonly modeled by introducing a stochastic process **Θ **= {Θ_*i *_: *i *= 1 ... *l*}, where the realizations *θ*_*i *_of the random variables Θ_*i *_are scalars from ${\mathbb{R}}_{+}$ that can be thought of as "scaling the tree" T leading to different substitution rates between two nodes at different sites *i*:

(1) $$P_{e}=e^{\theta_{i}\lambda (e)R}.$$

Different models for the space process have been introduced: Yang modeled **Θ **by an independently and identically distributed (i.i.d.) process with the random variables Θ_*i *_following a gamma distribution [17] and later proposed process models with memory [19]. Felsenstein used Hidden Markov Models and showed how to calculate the likelihood and estimate rates using the Viterbi algorithm [24]. In our work however, we assume the *θ*_*i *_to be deterministic parameters, assigned to every column in ***A***, without prior distribution. More complex models of evolution ***ψ ***are possible, e.g. including rates of insertions and deletions [25,26].

### Likelihood in phylogenetics

Efficient calculation of the likelihood function *p*(***A***; ***ψ***) has been introduced by Felsenstein over 20 years ago [27]. The Felsenstein Algorithm (FA) reduces the global likelihood problem to message passing along the branches of the tree from the leaves up to the root with local message calculation at the nodes. Consider an alignment column ***a***_*i*_, i.e. an observation at the leaves of the phylogenetic tree T resulting from the evolution of the unknown *i*th base in the sequence of the common ancestor. Let *u*, *v*, *w *∈ *V *be three nodes in T, *u *being the parent of *v *and *w*. Denote *b*_*u*_, *b*_*v*_, *b*_*w *_the bases at the respective node. The essential observation of the FA is that, given the base *b*_*u*_, the observations at the leaves of the subtree rooted on *v*, $a_{i}^{\left(v\right)}$, are independent of those of the subtree rooted on *w*, $a_{i}^{\left(w\right)}$. The conditional likelihood of the observation $a_{i}^{\left(v\right)}=[a_{i}^{\left(v\right)},a_{i}^{\left(w\right)}]$ is then given by [22]

(2) $$\begin{array}{lll} p(a_{i}^{\left(u\right)}|b_{u}) & = & \left(\sum_{b_{v}}p(a_{i}^{\left(v\right)}|b_{v})p(b_{v}|b_{u})\right)\\ & & \times \left(\sum_{b_{w}}p(a_{i}^{\left(w\right)}|b_{w})p(b_{w}|b_{u})\right), \end{array}$$

with the transition probabilities *p*(·|·) obtained from (1). Clearly, Eq. (2) depends on ***ψ***_*i *_which we omitted for the simplicity of notation. The initial message at leaf *q*_*j *_∈ *Q *is

$$p(a_{i}^{\left(q_{j}\right)}|b_{q_{j}})=\{\begin{array}{ll} 1 & \text{if }a_{i}^{\left(q_{j}\right)}=b_{q_{j}}\\ 0 & \text{else} \end{array},j=1...n.$$

At the root node *r *we finally obtain the likelihood for the *i*th column as $p(a_{i};\psi_{i})=\sum_{b_{r}}\pi_{b_{r}}p(a_{i}^{\left(r\right)}|b_{r})$ and using the i.i.d. assumption

(3) $$p(A;\psi_{i})=\prod_{i=1}^{l}p(a_{i};\psi_{i}).$$

## Results

### Application to ENCODE data

**Figure 1:**
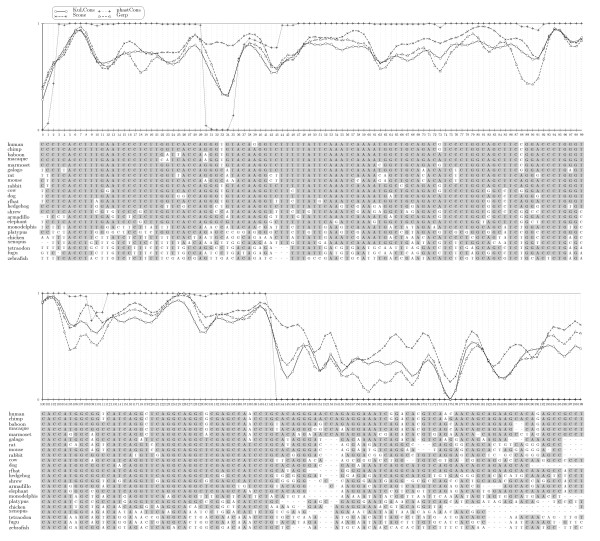
**Comparison of scores**. Comparison of KuLCons score signal to the phastCons, GERP and SCONE scores over an ENCODE region (hg17, ENm005, chr21:32677595-32677794). Scores have been smoothed using a Gauss window with *σ*_*w *_= 0.2 with size 15 (*δ *= 7). In order to facilitate comparison we plot the transformed version $1-\frac{\sigma_{i}}{\max_{i}\{\sigma_{i}\}}$ of our score and applied a similar transformation to the GERP scores in order to have scores in therange [0, 1]. In the alignment, bases with darker background represent bases identical to consensus.

Figure 1 compares KuLCons scores to the scores produced by phastCons, GERP and SCONE over a 200 bp nucleotide sequence alignment in an ENCODE region (ENm005). In order to facilitate the comparison, we show a transformed version of our score, that is

(4) $$1-\frac{\sigma_{i}}{\max_{i}\{\sigma_{i}\}},$$

where *σ*_*i *_denotes the conservation score as derived in the Section *Methods*. A similar transformation was applied to the GERP scores. This has the effect that 1 represents the highest possible conservation and zero the lowest, which is already the case in phastCons and SCONE scores. The transformation serves solely visualization purposes. Here, we would like to note that while normalized to be in the interval [0, 1] the scores can only be compared qualitatively as different scores are based on different models (see *Discussion*). For the calculation of our score, all parameters in ***ψ ***have been replaced by estimates except the rate heterogeneity parameter *θ*. We used the global average rate matrix ***R ***(non-conserved) published by Siepel et. al. [2]. However, using different realistic matrices had minor impact on the scores which is in accordance with previously published observations [9,18].

Single base resolution results in highly varying scores among columns. One can suggest that functional units, such as binding sites, are constraint at least over several neighboring base pairs. Assigning conservation to short regions and smoothing scores might thus be desirable. Furthermore, more reliable estimates on rates may be achieved using a sliding window when rates are correlated among adjacent sites. Therefore, KuLCons uses a window function which results in smoother scores (see *Methods*). The result in changing the size of the sliding window has a similar effect to the phastCons smoothness parameter.

PhastCons achieves smoothing by tuning the transition probabilities between the conserved/non-conserved states of its model and this smoothness parameter is chosen such that a predetermined coverage of conserved regions is achieved. Our method estimates the substitution rate incorporating neighboring columns in the maximum likelihood estimate and the specific smoothing effect of changing the window size will also depend on the window type used. Choosing a window size of one will result in single base resolution but the scores will be highly variable among neighboring columns (as in GERP and SCONE scores). Here, we applied the same window to smooth SCONE and GERP scores for comparison. It can be observed in Figure 1 that our score signal is in good agreement with the conservation estimate obtained by visual inspection of the multiple sequence alignment. The phastCons signal shows a binary characteristic and does not allow for discrimination among different conservation degrees. Consequently, phastCons shows a relatively rough-scale pattern of conservation which is different from the pattern by KuLCons, GERP and SCONE. This is explained by its underlying two-state phylo-HMM model (see *Discussion*).

Interestingly, the smoothed GERP and SCONE scores show a very similar characteristic to KuLCons with still some notable exceptions: in the region around 30 – 37 KuLCons and GERP indicate a relatively weak conservation while SCONE indicates higher conservation. On the other hand, KuLCons and SCONE both indicate higher conservation around 86 – 92 while GERP deviates significantly indicating weaker constraint. A different pattern can be observed in region 160 – 165 with KuLCons being intermediate. A plot over a 10, 000 basepair subregion of ENm005 is provided in *Additional file *2. In order to evaluate our method more thoroughly, we present simulation results in the next sections (additional simulations are provided in *Additional file *1).

### Sliding window ML estimation of a Markov Gamma process

In this Section, we show via simulations of synthetic data generated by a Markov Gamma process that our approach described in *Methods *is well suited for the estimation of conservation. I.i.d and Markov, continuous and discrete space models have been proposed for the rate process {Θ_*i *_: *i *= 1...*l*} along sites [21,24]. In the continuous case, the stationary distribution of {Θ_*i*_} is commonly assumed as a gamma distribution $p_{\Theta }(\theta )=x^{\alpha -1}\frac{e^{-\theta /\beta }}{\beta^{\alpha }\Gamma (\alpha )}=G_{\Theta }(\theta ;\alpha ,\beta )$[19]. Correlation among sites is introduced to account for the fact that neighboring sites are likely to experience similar substitution rates [18,20]. Discrete Markov models can be obtained by quantizing the range of *θ *in rate categories and calculating transition probabilities from the bivariate distribution of (Θ_*i*_, Θ_*i*+1_) [19] or using a Hidden Markov Model and estimating rate categories and transition probabilities from data [8,24].

Rate estimation has a long history in studies of molecular evolution. Yang derived the conditional mean estimator (CME) for *θ*_*i *_under a continuous i.i.d. gamma model which is known to minimize the mean squared error (MSE) and having the highest correlation (Corr(*θ*_*i*_, ${\hat{\theta }}_{i}$)) between true *θ *and estimated $\hat{\theta }$ value. However, the method requires knowledge about the prior distribution of Θ and it was shown in [18] that rate estimates are sensitive to the choice of the parameters of the distribution. In addition, in the context of application to whole genome alignments the method is computationally too time consuming. A low complexity version of the CME approximates the rates via discrete rate categories [17]. The discrete CME has also been derived in a Markov chain framework with rate categories derived from an underlying bivariate gamma distribution of adjacent sites. It was shown that the discrete approximation achieves almost the same accuracy as the continuous version when using a sufficient number of categories [19]. However, in order to find a good partitioning of the categories, a prior distribution on T has to be assumed. Models of among-site rate variation were reviewed in [28].

#### Simulation model

**Figure 2:**
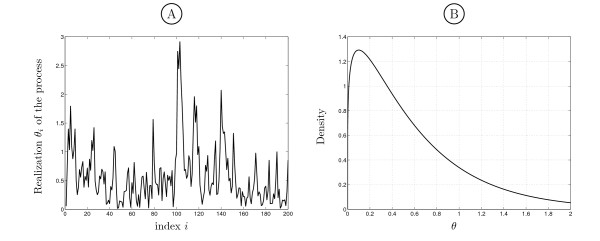
**Sample realization of the simulated Markov Gamma process**. A: Sample path of {Θ_*i*_} with marginal *G*_Θ_(*θ*, 1.2, 0.5) and *ρ*_*θ *_= 0.7. B: Marginal probability density of *θ *used in the simulation.

In the context of conservation measurement, the estimator is not required to give reliable results on the whole spectrum of possible rates, but to provide a good estimate for the degree of conservation of a region. The situation that we simulate mimics a moderately conserved region with "islands" of more or less conservation due to variance and autocorrelation of the rate. A good conservation estimator will take into account autocorrelation among sites while retaining the sensitivity of reporting variability within regions. Using a Markov gamma rate model, we generated alignment columns and estimated the rates using site-by-site ML estimation and the sliding window Maximum Likelihood procedure described in *Methods*. Simulation of Markov gamma processes was performed as described by Moran [29] and Phatarfod [30]. The rates *θ*_*i *_follow a process with a stationary distribution *G*(*θ*_*i*_; 1.2, 0.5), i.e. *E*{Θ} = 0.6 and VAR(Θ) = 0.3, and correlation Corr(*θ*_*i*_, *θ*_*i*+*j*_) = $\rho_{\theta }^{j}$ among sites. Analysis of substitution rates has shown that *θ *is mostly in the range [0, 1] (for the chosen parameters in this simulation, 80% of the *θ*_*i *_are expected to fall in this interval) and we simulate an overall moderately conserved region (*E*{Θ} = 0.6) with varying conservation inside, which is modeled by the rate variance (VAR{Θ} = 0.3) and autocorrelation. In Figure 2 a sample realization of the rate process {Θ_*i*_, 1...*l*} is shown for *l *= 200 with the parameters described above and *ρ*_*θ *_= 0.7 revealing several regions with different degrees of substitution rates. Alignment columns were simulated under the described model on a subtree of the 28 species ENCODE tree comprising 18 species.

#### Simulation results of rate process estimation using sliding window Maximum Likelihood

**Figure 3:**
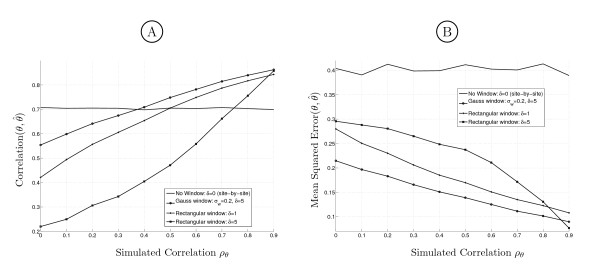
**Performance comparison**. Performance of ML estimation of a Markov gamma process using different window functions. A: Correlation between true (*θ*) and estimated ($\hat{\theta }$) rate. B: Mean squared error.

The true simulated *θ *is compared to its estimate $\hat{\theta }$ obtained by the different methods. In Figure 3 two performance measures are shown, the MSE and Corr(*θ*, $\hat{\theta }$), for different window types over the range of among site rate autocorrelation *ρ*_*θ*_. For site-by-site ML estimates we restricted the maximum value of $\hat{\theta }$ to 3 because it was reported by Nielsen that estimates of highly variable columns tend to go to infinity [20]. Around 99% of *θ *will have values lower 3 under the assumed gamma distribution. Choosing different maximum values had minor effects on the results.

The best MSE is achieved with the Gauss window of variance 0.2 (Eq. (5) with *σ*_*w *_= 0.2) in the complete range of *ρ*_*θ*_. For very slowly changing rates (*ρ*_*θ *_= 0.9) the performance coincides with the large rectangular window. Interestingly, for uncorrelated sites, the large Gauss window clearly gives the best results, outperforming the small rectangular window and site-by-site estimation. Apparently, even though the window introduces a bias, the error variance is reduced, obviously leading to an overall performance improvement. The maximum correlation Corr(*θ*, $\hat{\theta }$) and the minimum MSE are achieved. This suggests that the method is very well suited for estimating *θ *with unknown prior distribution and with arbitrary autocorrelation among adjacent sites. A similar processing could be based on a window version of the Bayesian approach with rate categories [17].

### Statistical analysis of the proposed ML based estimate

As the proposed ML estimate is based on a relatively small sample size, we study the density of the estimated rate variation $\hat{\theta }$ and compare it to the theoretically achievable pdf. We assumed all parameters in ***ψ***_*i *_to be fixed except for *θ*_*i*_, reducing the problem to scalar parameter estimation. We check whether the ML Estimator (MLE) attains the Cramér-Rao lower bound for the small sample size

$$E\left\{{\left(\hat{\theta }-\theta \right)}^{2}\right\}\geq -E{\left\{\frac{\partial^{2}\log p(x;\theta )}{\partial \theta^{2}}\right\}}^{-1}=\frac{1}{I(\theta )}.$$

**Figure 4:**
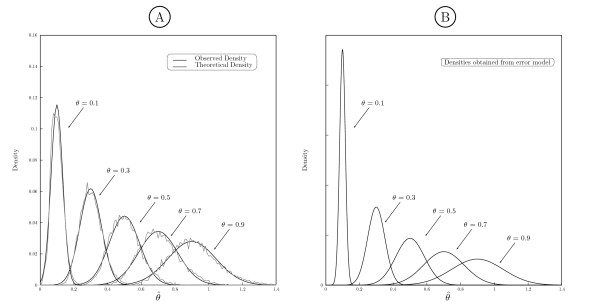
**Variance analysis and error model**. A: Observed and theoretical pdfs of estimates for *δ *= 7 simulated at different *θ*. B: Densities of rate heterogeneity estimates under multiplicative error model.

It is well known that the MLE asymptotically achieves this bound for large sample sizes, i.e.$\hat{\theta }\overset{a}{\sim }N(\theta ,I{\left(\theta \right)}^{-1})$, where N(*μ*, *σ*^2^) denotes the normal distribution with mean *μ *and variance *σ*^2^. We performed a computer simulation using 100000 realizations of alignments of length (2*δ *+ 1), generated according to a fixed evolutionary model ***ψ***. We estimated $\hat{\theta }$ and computed I(*θ*) for each sample. Figure 4 shows the theoretical achievable pdfs N(*θ*, *I*(*θ*)^-1^) versus the observed pdfs of $\hat{\theta }$ for different simulated *θ*. Even for small window sizes, e.g. *δ *= 7, the MLE closely approaches its asymptotic distribution. At low values of *θ*, the variances are relatively small, i.e. different values of *θ *can be distinguished with high probability. It can also be observed that the variance of the estimation increases with increasing *θ*. Hence, our estimator is best discriminating between different degrees of conservation in relatively conserved regions even at small window sizes whereas in non-conserved regions, the information revealed by the window is not enough to allow for precise differentiation. The accuracy increases with the number of species in the alignment. These results can be used to identify whether a region is more conserved than another: we propose an estimation model for *θ *with a multiplicative error

$$\hat{\theta }=(1+\eta )\theta ,$$

where $\eta \sim N(0,\sigma_{\eta }^{2})$ is a normally distributed random variable. This has the effect that the variance of the estimation will depend on its mean and higher values will have a higher variance such as observed in Figure 4. The best fitting variance $\sigma_{\eta }^{2}$ can be determined via simulations on synthetic data and a log likelihood ratio test can subsequently be performed to detect differentially evolving regions with statistical significance. The multiplicative variance will depend on the tree and other parameters used. A simulation of the multiplicative model is also shown in Figure 4, demonstrating that it fits very well the distribution of estimates obtained from the simulated genomic data.

### Conservation score respecting InDel history

**Figure 5:**
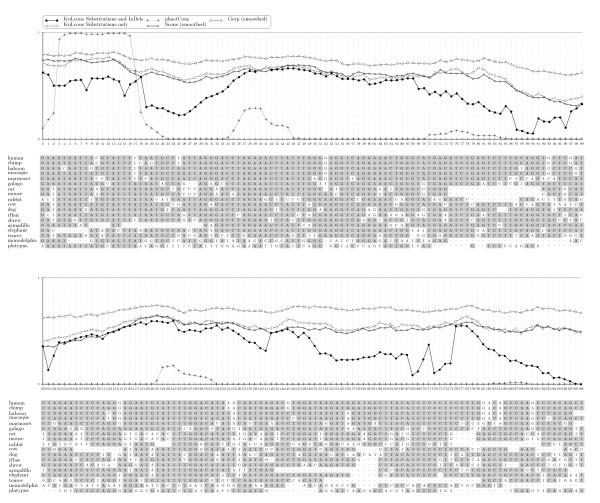
**Comparison of conservation scores under the extended phylogenetic InDel model**. Comparison of KuLCons score taking gaps as InDels into account and KuLCons score treating them as missing data in an ENCODE region (hg17, ENr212, chr5:142147118-142147317). Scores are based on estimating the parameters in a rectangular window with *δ *= 10. The Figure also shows phastCons, GERP and SCONE scores for comparison.

The KL projection allows a whole set of parameters to contribute to the conservation score in a probabilistic framework. As a possible application, we considered an extended evolutionary model to obtain a score that probabilistically incorporates insertion and deletion events. These InDels give rise to gaps in the alignment which are usually neglected when measuring the conservation. Figure 5 shows two different scores for a 200 bp fragment of an ENCODE region. One score represents conservation estimation based only on local substitution rate estimates, neglecting gaps. For the other score, 3 parameters have been estimated: the substitution rate *θ*, and InDel parameters ${\hat{c}}_{I}$ and ${\hat{c}}_{D}$. The program Indelign [26] was used to estimate ${\hat{c}}_{I}$ and ${\hat{c}}_{D}$. All parameters were estimated in a rectangular sliding window of length 21 over the alignment. Note that in this case ***ψ ***comprises 2 additional parameters *c*_*I *_and *c*_*D*_. Probabilities ${\hat{p}}_{I}^{\left(e,k\right)}$ and ${\hat{p}}_{D}^{\left(e,k\right)}$ of an insertion or deletion of length *k *= 1, 2, .., 2*δ *+ 1 on branch *e *were derived from ${\hat{c}}_{I}$ and ${\hat{c}}_{D}$ as described in [26]. The probability of a fully conserved column is then given as the probability of absence of mutations (substitution, deletion and insertion) in each branch and the score is the KL divergence between the probabilities of a fully conserved column under the estimated model and under the maximum conserving process (see details in the *Methods *section). Obtained KuLCons scores are further compared to phastCons, GERP and SCONE. The latter method is also accounting for InDel events. In [31], Siepel et al. present an extension of phastCons accounting for lineage-specific "gained" or "lost" elements. Similar to our approach the authors use a separately reconstructed InDel history and compute emission probabilities of InDels for a phylo-HMM. However, to our knowledge phastCons has not yet been further developed in this direction and the signal of phastCons shown in Figure 5 treats gaps as missing data. As expected, the KuLCons score including the InDel estimation is always lower or equal to the InDel neglecting version. The scores coincide where no gaps are observed in the sliding window (positions 40–41) and differ when one or more gaps are observed (e.g., 72–96). A significant difference in the scores is observed in regions with many gaps. While the score based solely on the substitution rate indicates high conservation, the score respecting the gaps indicates low conservation. Compared to KuLCons, gaps seem to be far less penalized by the SCONE score which does not show notable deviations in the gappy regions.

## Discussion

In Figure 1 we showed a comparison of KuLCons to phastCons, GERP and SCONE. The methods aim to detect sequence conservation and/or constraint based on different models: phastCons quantizes the rate heterogeneity parameter in two different categories. One category represents constrained evolution and the other neutral evolution which are modeled as the states of a phylogenetic-Hidden Markov Model (phylo-HMM) each associated with different ***ψ ***[8]. PhastCons scores reflect the a posteriori state probabilities of the HMM and thus express the probability of constraint, based on the underlying degree of conservation and the assumptions about neutral evolution imposed on the Hidden-Markov model. While this is very well suited for high throughput processing, a simplistic binary model on genome evolution is imposed. The two state HMM implies that evolution is either conserving or neutral. The model has to be tuned with a priori information such as transition rates among the conserved and the neutral state, which implicitly imposes assumptions about the expected length and coverage of conserved regions. The result of the binary model can be clearly observed in Figure 1 providing clear indication for strong or weak conservation but lacking sensitivity for different degrees of conservation. GERP compares observed and expected substitution rates on a phylogenetic tree with fixed topology. The branch lengths of the observed tree are estimated for each column separately and branch lengths of the expected tree are based on the average of estimates from neutral sites. The final score is the difference of the observed to the expected substitution rate induced by the corresponding estimated trees [9]. GERP predicts constraint elements using a null model of shuffled alignments.

SCONE scores express the p-value that a position evolved neutrally given a model that accounts for context-dependency, InDel events and neutral evolution. Hence, the score can as well be interpreted as a probability of constraint [10].

Another method used in the ENCODE analysis, BinCons developed by Margulies et al. [7], was not included in the comparison because it was noted by Siepel [8] that scores of BinCons and phastCons give qualitatively similar results. In contrast to the approaches mentioned above, KuLCons considers the direct estimation of the rate heterogeneity *θ*_*i *_∈ ${\mathbb{R}}_{+}$ or more parameters from an evolutionary model ***ψ ***via Maximum Likelihood using an optimized sliding window. The Kullback-Leibler divergence is used to project the estimated parameters to a conservation score. The rate parameter *θ *is the crucial parameter for detecting evolutionary conservation and the ML sliding window approach *in silico *can achieve high estimation accuracy assuming a model of gamma distributed rates with autocorrelation. We believe that KuLCons has the following advantages:

1. The presented algorithm is free of assumptions about neutral evolutionary rates that are notoriously hard to determine [11,12,15]. Furthermore, it uses few a priori parameters that require biological considerations. We have shown that our ML estimation of substitution rates in an optimized Gauss window without assumptions on the rate prior leads to good performance in the MSE sense.

2. Our score reflects well the different degrees of conservations and is in accordance with state-of-the-art methods. This soft score may disclose new possibilities in comparative genome analysis allowing the comparison of different finescale conservation patterns within conserved regions of interest.

3. It is possible to extend the phylogenetic model as long as a distribution on the columns of the alignment is induced. A whole set of different process parameters can then be mapped to a conservation score via the Kullback-Leibler divergence. A score was shown in Figure 5 that uses co-estimated InDel rate parameters. Another possibility would be to assign different *θ *to different subtrees thus allowing for lineage-specific rate heterogeneities.

Our results show that the KuLCons score qualitatively exhibits similar conservation patterns in different regions as GERP and SCONE. This observation has two important consequences: first, it is possible to score the conservation of DNA sequences without having assumptions or estimates on neutral rates. The estimation and potential bias of these rates have been controversially discussed in the past [11,12,15,16]. Secondly however, our results suggest that conserved elements inferred from this method will probably not be very different from those discovered by GERP and SCONE opposed to the conjecture raised in [15]. This would mean that the discrepancies of experimentally verified functional elements and computationally predicted conserved regions [14,32,33] cannot be explained in majority by biased assumptions on neutral rates. One explanation might be that low scoring sequences experience constraints at a different information level (e.g. structure) that is not directly detectable by simple sequence alignments but rather structural alignments. An alternative explanation is that species specific functional elements that are not conserved across a given set of species are more important in functional evolution than currently discussed.

## Conclusion

We presented and evaluated a novel method for the calculation of sequence conservation scores over multiple sequence alignments. Opposed to existing methods, we avoid estimates of neutral substitution rates by testing divergence from perfectly conserved columns on the assumption that these represent maximum conservation. Furthermore our method does not assume a prior distribution on the rate heterogeneity and does not require prior tuning. Our simulation results suggest that local ML estimation of substitution rates in a sliding Gauss window can achieve a high accuracy in detecting patterns of conservation. We qualitatively compared our score to the scores of established methods (phastCons, GERP and SCONE) in ENCODE regions and found that our algorithm is well suited for discriminating among different degrees of conservation and reveals good accordance with scores produced by GERP and SCONE. We find that even though KuLCons differs from GERP and SCONE in several regions it does not seem to indicate surprisingly different conserved elements. A strong advantage of our approach is that it also allows for multiple parameters to contribute to the conservation score in a probabilistic framework and thus can for example account for insertions and deletions which many other known methods do not.

## Methods

### Data

ENCODE alignments (hg17, TBA alignment [34]) along with the corresponding phylogenetic tree as well as phastCons, GERP and SCONE scores (TBA Cons track) have been obtained from the UCSC Genome Browser [35]. Columns with gaps in the human sequence were removed. Scores (except phastCons) have been smoothed using a sliding window to make them comparable. The same window type and size has been applied to the scores.

### Conservation scores

In the framework of conservation estimation, a subset of parameters in ***ψ ***will be fixed over the alignment. For example, it is reasonable to assume that the topology T and the branch lengths *λ*(*e*) do not change considerably over alignment columns. These parameters as well as the stationary distribution ***π ***are generally replaced by their ML estimates from a large data set. In the following, we use $\hat{\psi }$ to refer to the estimate of the free parameters in ***ψ***.

We use a sliding window meaning that we use a section of the alignment $A_{i-\delta }^{i+\delta }=[a_{i-\delta },...,a_{i+\delta }]$, *i *= *δ *+ 1, ..., *l *- *δ *of length 2*δ *+ 1 around the column of interest to estimate the parameters that most likely resulted in the alignment observed in this window (for convenience, we restrict ourself to odd window sizes. However, the generalization to even window sizes is trivial). We use a window function *w*[*n*] to weigh the likelihoods of neighboring columns. For example, an equally weighting rectangular window could be chosen *w*[*n*] = 1, *n *= 0..2*δ*. It is well known that the choice of window functions with good spectral properties can significantly improve estimation. Several types of window functions optimized with respect to different properties exist, e.g. the Hamming-, Kaiser- or Gauss-window. The Gauss-window for example is given as

(5) $$w[n]=\{\begin{array}{ll} e^{-\frac{1}{2}{\left(\frac{n-\delta }{\sigma_{w}(n-\delta )}\right)}^{2}} & n=0..2\delta \\ 0 & \text{else} \end{array}.$$

The resulting estimator is then given by

(6) $${\hat{\psi }}_{i}=\underset{\psi_{i}}{\arg\max}\left\{\sum_{n=i-\delta }^{i+\delta }w[n-i+\delta ]\log(p(a_{n};\psi_{i}))\right\},$$

yielding a set of parameters describing the local ML evolutionary process for the data in the window. In order to obtain a scalar conservation score from the estimated parameters ${\hat{\psi }}_{i}$, we consider the probability mass function (pmf) of an alignment column that is parameterized by ${\hat{\psi }}_{i}$, *p*(***a***;${\hat{\psi }}_{i}$). Avoiding assumptions about the neutral evolutionary rate, we compare the estimated distribution to the distribution of the well defined absolute conservation, parameterized by the imaginary set of parameters ***ψ***^0 ^that does not allow for any substitution to occur, i.e.

$$p(a;\psi^{0})=\{\begin{array}{ll} \pi_{b} & \text{if }a^{\left(q_{j}\right)}=a^{\left(q_{k}\right)}=b\in A,\forall (j,k)\in {\left\{1,..,n\right\}}^{2},q_{j},q_{k}\in Q\\ 0 & \text{else} \end{array}.$$

A measure for the divergence between two probability mass functions is the Kullback-Leibler (KL) divergence (often termed relative Entropy) which is well established in statistics and information theory [36]. Let X be a discrete alphabet and *p*(*x*), *q*(*x*) two pmfs on X. The Kullback-Leibler divergence D(*p*||*q*) is defined as

$$D(p||q)=\sum_{x\in X}p(x)\log\frac{p(x)}{q(x)},$$

with the convention that $0\log\frac{0}{q}=0$ and $p\log\frac{p}{0}=\infty$. It can be shown that D(·||·) ≥ 0 with equality iff *p *= *q*. Denote *p*(***a***; ${\hat{\psi }}_{i}$) the pmf of a column ***a ***in the alignment generated by the ML evolutionary process, $\hat{\psi }$, estimated from $A_{i-\delta }^{i+\delta }$. Our conservation score function is given by

(7) $$\sigma ({\hat{\psi }}_{i})=D(p(a;\psi^{0})||p(a;{\hat{\psi }}_{i})).$$

As we measure the divergence to the maximum conservation, low score values indicate high conservation. Note that *p*(***a***; ***ψ***^0^) is equal to zero whenever $\exists (j,k):a^{\left(q_{j}\right)}\neq a^{\left(q_{k}\right)}$, i.e. it is only nonzero for fully conserved columns. That is in order to evaluate Eq. (7) we only have to consider the four columns having only As, Cs, Gs or Ts which are the only possible realizations of maximum conservation. Let $p(a_{\left[b\right]}^{\left(r\right)};\psi )$ denote the probability of a fully conserved column under ***ψ***, i.e. $a_{\left[b\right]}^{\left(r\right)}=[b,b,...,b]$, b ∈ A. Then, $p(a_{\left[b\right]}^{\left(r\right)};\psi^{0})=\pi_{b}$ under the maximum conserving model and we can rewrite Eq. (7) as

$$\sum_{b\in A}\pi_{b}\log\frac{\pi_{b}}{p(a_{\left[b\right]}^{\left(r\right)};{\hat{\psi }}_{i})}.$$

Our algorithm works as follows: given an alignment ***A***, we choose a suitable window type and fix the size of our sliding window by choosing a suitable *δ*. Then we obtain the local ML estimate ${\hat{\psi }}_{i}$ over $A_{i-\delta }^{i+\delta }$ according to (6) by message passing (FA) and maximization using a Newton method. The estimate is projected to a score via Kullback-Leibler divergence according to (7) and assigned to the column ***a***_*i*_. The sliding window is shifted forward, increasing *i *by 1 and the procedure is repeated until *i *reaches *l *- *δ*. A score *σ*_*i *_is now assigned to every alignment column ***a***_*i *_(scores at the borders of the alignment can be obtained by setting *p*(***a***_*n*_;***ψ***_*i*_) = 1 for *n *< 1 and *n *> *l*).

### Run time of the algorithm

Even though the run time of our algorithm is significantly higher than the computation times achieved by algorithms designed for high throughput analysis such as phastCons, our method is still feasible for assaying whole genome alignments. Using a single standard Linux PC (2 Gb RAM, 2.4 GHz) it was possible to calculate the scores for the human (hg18) reference 28-species alignment from UCSC Genome Browser [35] in less than 1 month. The complexity of the algorithm scales linear with the length of the alignment and linear with the number of inner nodes in the inspected phylogenetic tree. The latter is explained by the complexity of the Felsenstein algorithm that has to visit every node in the tree where the same update function is computed. The following modifications can significantly reduce the complexity of the algorithm:

1. Several authors have shown that estimation of substitution rates and thus detection of constraint is not sensitive on the choice of the rate matrix ***R***. Yang et al. showed that rate heterogeneity estimates under different models do not deviate significantly [18] and the authors of GERP [9] also state that using different realistic rate matrix models had negligible impact on their estimates. Our own results support these findings. Costly numerical evaluation of the matrix exponential can be avoided by using rate matrices with known analytical solution of the induced probability transitions (such as F84, HKY85, TN93 [21,23]). This reduces the running time of the algorithm.

2. We are currently investigating a low-complexity method for inference on a phylogenetic tree using metrics instead of probabilities. The technique known as log-max approximation has shown to provide sufficient accuracy while significantly reducing the algorithmic complexity in data transmission applications [37]. The possible applications of this method in the context of conservation measurement and phylogenetic inference will be addressed in a future contribution.

### Extending ***ψ ***to model Insertion and Deletion events

The KL projection allows a whole set of parameters from ***ψ ***to contribute to the conservation score. We extended the standard evolutionary model to include rates of insertions and deletions. These InDels give rise to gaps in the alignment which have usually been neglected when measuring the conservation. The probabilistic inference of insertion and deletion events along a phylogenetic tree is a difficult problem. Several methods have been proposed in the literature. Rivas showed in [38] how to extend the matrix ***R ***in order to model gaps as a fifth character. A maximum likelihood approach for inferring InDel scenarios was proposed by Blanchette et. al. in [25]. Recently, Kim and Sinha presented an algorithm, InDelign, for the annotation of InDels in a probabilistic framework [26]. Here we used InDelign to estimate the probability of deletions and insertions in the sliding window. The estimated probabilities ${\hat{p}}_{I}^{\left(e,k\right)}$ and ${\hat{p}}_{D}^{\left(e,k\right)}$ of an InDel of length *k *= 1, 2, .., 2*δ *+ 1 on branch *e *were calculated. InDel probabilities *p*_*I*_, *p*_*D *_are assumed to be proportional to the branch length *λ*(*e*)

*p*_*I *_= *c*_*I*_*λ*(*e*), *p*_*D *_= *c*_*D*_*λ*(*e*),

where *c*_*I *_and *c*_*D *_are constants, estimated as follows: Let *N*_*I*_,*N*_*D *_be the numbers of InDels from the parent of the two closest related species to either species. The constants are estimated as

$$\begin{array}{cc} {\hat{c}}_{I}=\frac{N_{I}}{(\lambda (e_{1})+\lambda (e_{2}))L}, & {\hat{c}}_{D}=\frac{N_{D}}{(\lambda (e_{1})+\lambda (e_{2}))L}, \end{array}$$

where (*λ*(*e*_1_) + *λ*(*e*_2_)) is the sum of the distances of these species and *L *the length of the sequence which is in our case the size of the window 2*δ *+ 1 [26].

The score was then calculated based on the 3 parameters using the KL divergence. As we are only interested in the probability of a fully conserved column, we have to calculate the probability that such a column is observed for the estimated values. The substitution and InDel processes are assumed to act independently, i.e. let again $p(a_{\left[b\right]}^{\left(r\right)};{\hat{c}}_{I},{\hat{c}}_{D},\hat{\theta })$ denote the probability of a fully conserved column under the estimated parameters, then $p(a_{\left[b\right]}^{\left(r\right)};{\hat{c}}_{I},{\hat{c}}_{D},\hat{\theta })=p(a_{\left[b\right]}^{\left(r\right)};{\hat{c}}_{I},{\hat{c}}_{I})p(a_{\left[b\right]}^{\left(r\right)};\hat{\theta })$. Due to assumptions on InDel events imposed by the InDelign algorithm, the first term corresponds to the probability that no InDel occurred on any of the branches *e*, at the actual and the *k *preceeding positions:

$$p(a_{\left[b\right]}^{\left(r\right)};{\hat{c}}_{I},{\hat{c}}_{D},\hat{\theta })=p(a_{\left[b\right]}^{\left(r\right)};\hat{\theta })\prod_{\forall e}\prod_{k=1}^{2\delta +1}{\left(1-p_{D}^{\left(e,k\right)}\right)}^{k}{\left(1-p_{I}^{\left(e,k\right)}\right)}^{k}.$$

Eq. (7) can then be evaluated using this probability.

## Availability

Conservation scores for hg18 are available for comparison from our website 

## Authors contributions

JD, PH, JCM and JH conceived the concepts and methods. JD performed the simulations and prepared the manuscript. NL performed part of the simulations on the InDel scores. JCM and JZ acted as advisors in biological modeling and interpretation. All authors contributed to the discussion and have approved the final manuscript.

## Supplementary Material

Additional file 1 Supplementary Material. Additional performance analysis of our method and further comparisons of the scores in ENCODE regions. Click here for file

Additional file 2 Scores in 10,000 bp ENCODE region. A comparison of KuLCons, phastCons, GERP and SCONE scores in a 10,000 bp ENCODE region (hg17, ENm005, Chr21:32668244-32678960). Click here for file
