# Supplementary material for: Local conservation scores without a priori assumptions on neutral substitution rates
Source: BMC Bioinformatics. 2008 Apr 11;9:190. doi: 10.1186/1471-2105-9-190 (PMC2375903; doi:10.1186/1471-2105-9-190)
Supplement: Additional file 1 — Supplementary Material. Additional performance analysis of our method and further comparisons of the scores in ENCODE regions. [file 1471-2105-9-190-S1.pdf]

Supplementary Material

Local Conservation Scores without a priori Assumptions on Neutral Substitution Rates

Dingel*et al.

Email: janis.dingel@tum.de;

*Corresponding author

Performance Evaluation on Synthetic Data

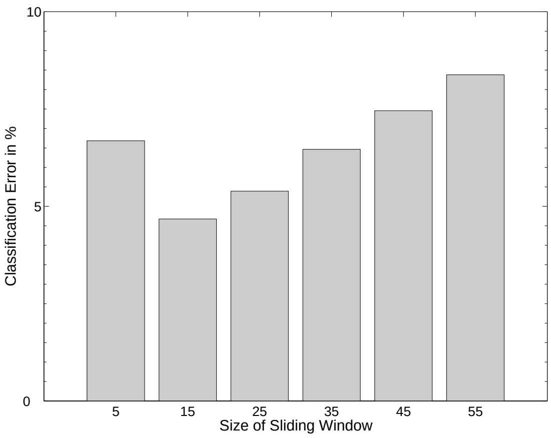

(a) Featuresize 35.

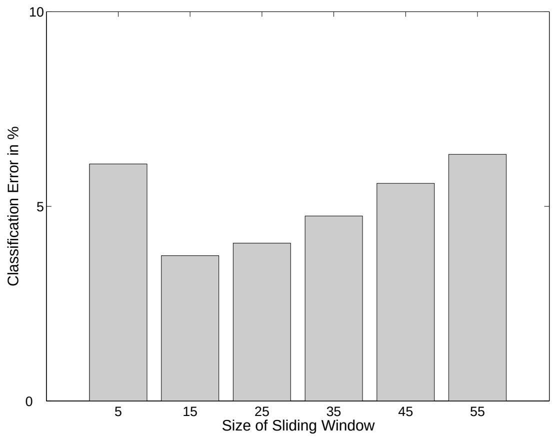

(b) Featuresize 50.

Figure S1: Classification performance of our estimator for different choices of δ and simulated featuresizes 35 and 50.

We present simulation results that try to evaluate the performance of our estimator when used as a detector for conserved regions with hard decision. We use the following setup: we generate synthetic alignment blocks $\boldsymbol{A}_n$ of variable sizes l_n from a given tree of 17 species obtained from UCSC [1]. For $\boldsymbol{R}$, we use the global averages of estimated matrices of Siepel et. al. available from http://www.soe.ucsc.edu/˜acs/conservation/. The block lengths are sampled from an exponential distribution (rounded to integer values) with mean $E\{l_n\}$, representing an expected "feature size". To each block, we assign a parameter $\theta_n \in \{0, 0.1, 0.2, \ldots 1.0\}$, where $\theta_n = 0$ leads to maximum conservation and

$\theta_n = 1$ represents minimal conservation. The θ_n are drawn at random following a uniform distribution. The blocks $\boldsymbol{A}_n$ are appended to form a large alignment $\boldsymbol{A} = [\boldsymbol{A}_1, \boldsymbol{A}_2, ...]$ of length $l = \sum_n l_n$. In this performance analysis we aim for a hard decision, thus we fix an arbitrary threshold $\theta^* \in \{0, 0.1, \ldots, 1.0\}$ which we will use a posteriori to classify the columns $\boldsymbol{a}_i$ as "conserved" or "nonconserved". We obtain the scores σ_i for $\boldsymbol{A}$ and transform our score as described in the *Methods* section. We define a cost function $c_i : [0, 1] \to \{0, 1\}$ that indicates missclassification of site i depending on a global score threshold $\zeta \in [0, 1]$

$$c_i(\zeta) = \begin{cases} 1 & (\sigma_i > \zeta) \wedge (\theta_i > \theta^*) \\ 1 & (\sigma_i < \zeta) \wedge (\theta_i < \theta^*) \\ 0 & \text{else} \end{cases} . \tag{1}$$

The total number of missclassified columns is then $\mathcal{E}(\zeta) = \sum_{i=\delta+1}^{l-\delta} c_i(\zeta)$. The performance is evaluated at the global threshold $\hat{\zeta}$ that minimizes the overall number of classification errors $\hat{\zeta} = \arg\min_\zeta \{\mathcal{E}(\zeta)\}$. Figure S1 shows the $\mathcal{E}(\hat{\zeta})$ of the method for different $E\{l_n\}$ with conservation defined as $\theta^* \leq 0.3$. The results are shown for different window sizes δ. The size of the sliding window achieving the best trade off for this simulation on 17 species seems to be around 15. Unsurprisingly, when the expected feature size $E\{l_n\}$ is very small, the overall classification performance of the MLE decreases significantly with growing window size δ (results not shown).

Additional Results on ENCODE regions

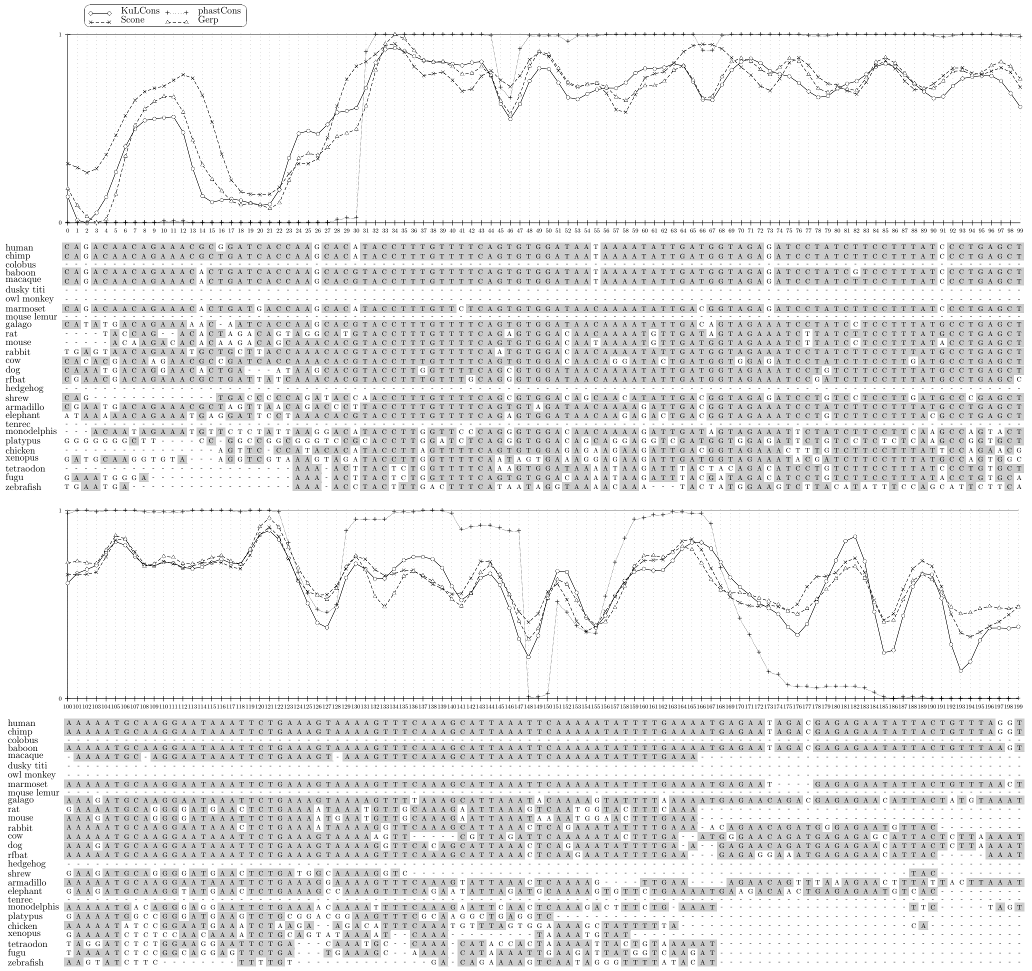

Figure S2: Comparison of our score signal (KuLCons) to the phastCons, GERP and SCONE scores over an ENCODE region (hg17, ENm005, chr21:32669544-32669743). KuLcons used a Gauss window with $\sigma_w = 0.2$ and the size was set to 15 ($\delta = 7$). SCONE and GERP have been smoothed using the same window. In order to facilitate comparison we plot the transformed version $1 - \frac{\sigma_i}{\max_i\{\sigma_i\}}$ of our score and a similar transformation has been applied to the GERP scores in order to have scores in the range $[0, 1]$.

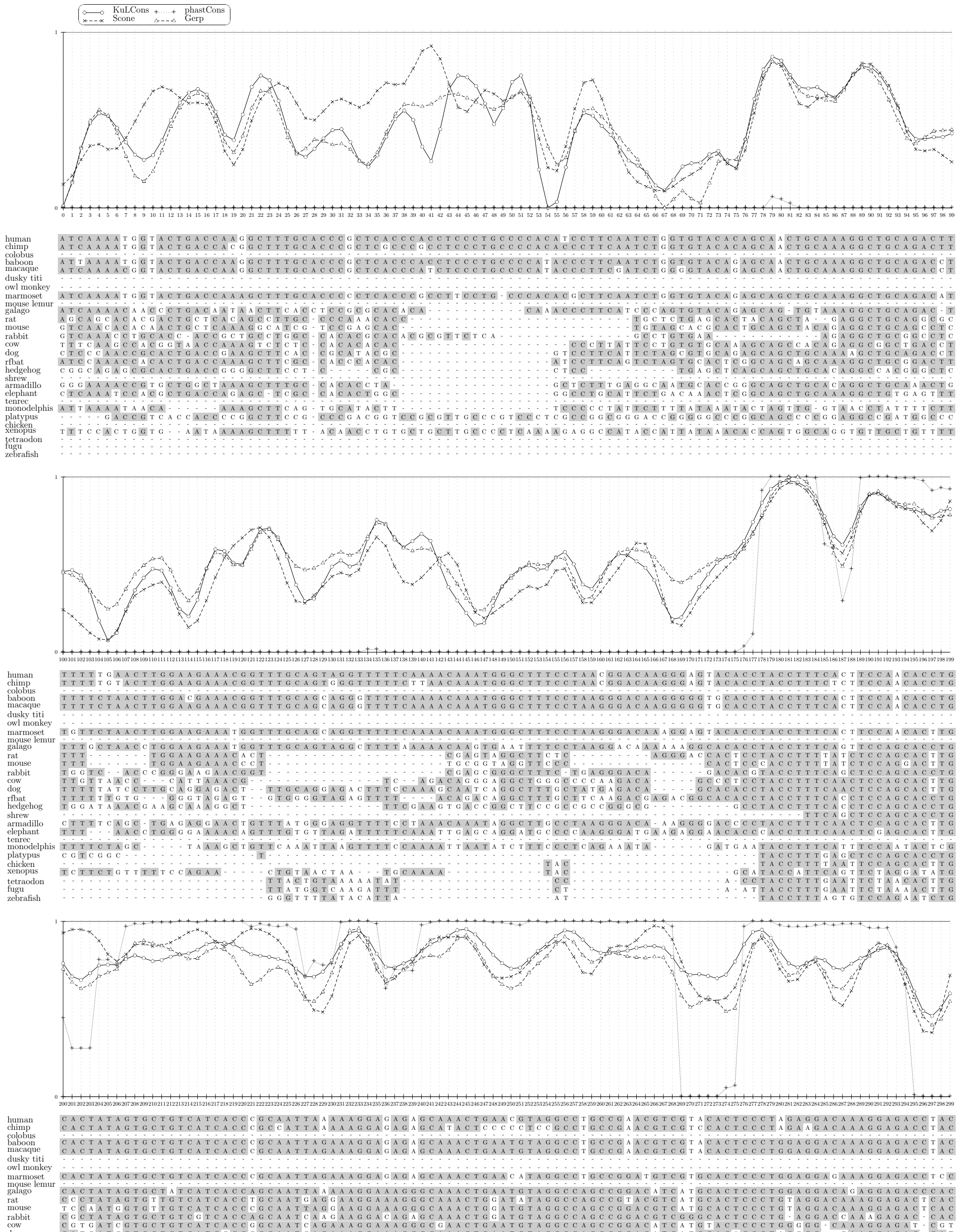

Figure S3: hg17, ENm005, chr21:32672444-32672743

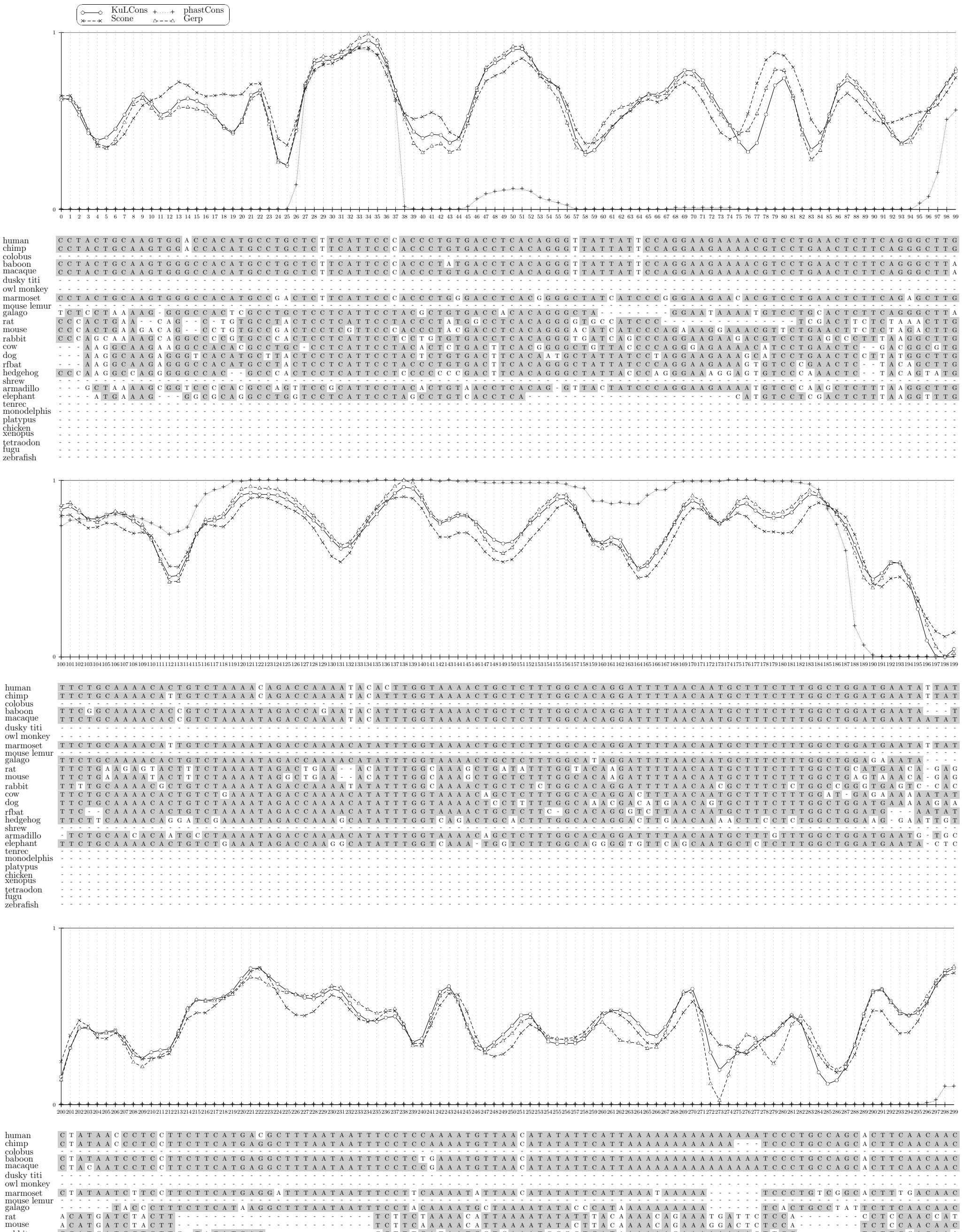

Figure S4: hg17, ENm005, chr21:32675344-32675643

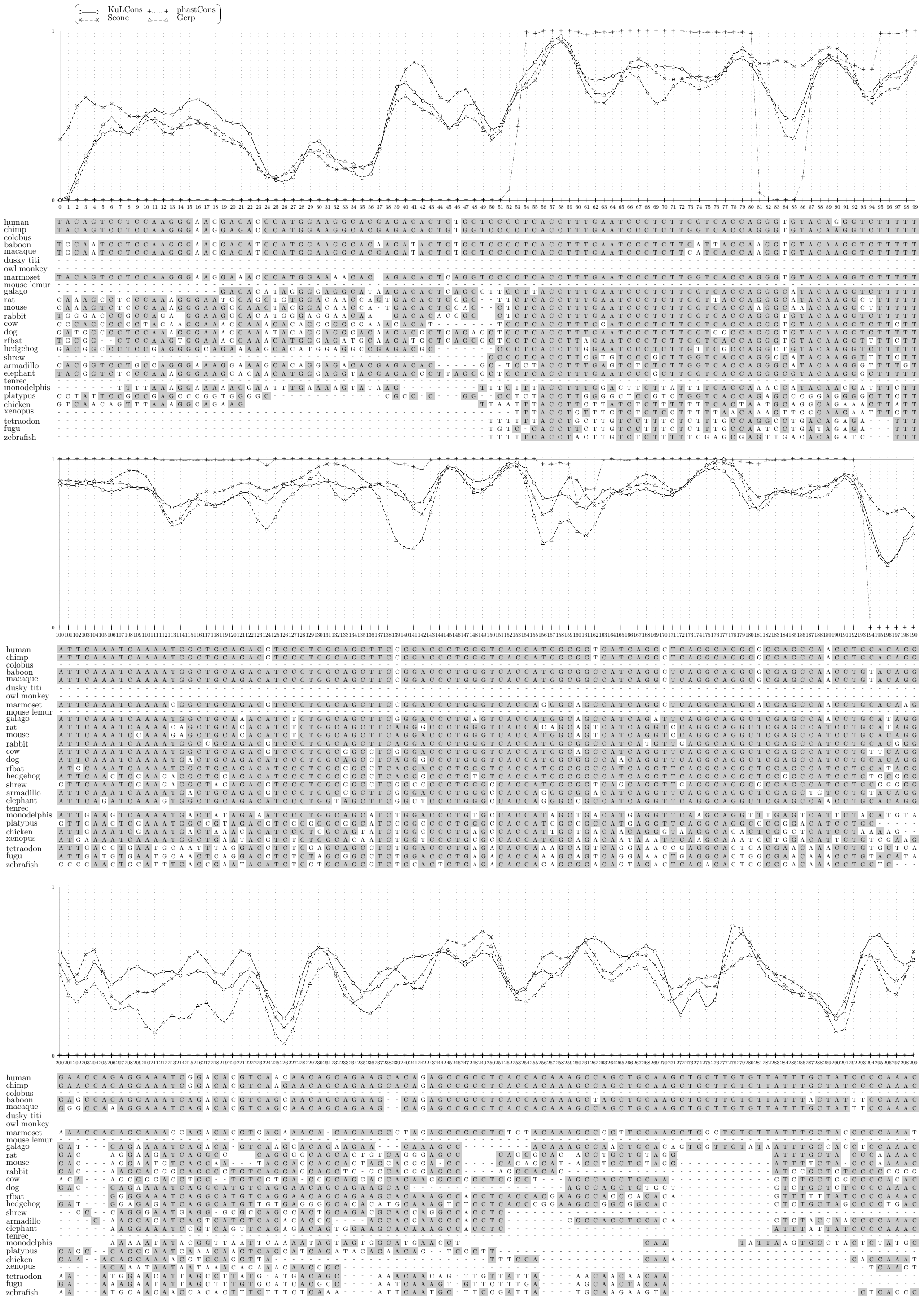

Figure S5: hg17, ENm005, chr21:32677544-32677843

References

1. Karolchik D, Baertsch R, Diekhans M, Furey TS, Hinrichs A, Lu YT, Roskin KM, Schwartz M, Sugnet CW, Thomas DJ, Weber RJ, Haussler D, and WJK: **The UCSC Genome Browser Database.** *Nucleic Acids Res* 2003, **31**:51–4.
