# Supplementary material for: Local conservation scores without a priori assumptions on neutral substitution rates
Source: BMC Bioinformatics. 2008 Apr 11;9:190. doi: 10.1186/1471-2105-9-190 (PMC2375903; doi:10.1186/1471-2105-9-190)
Supplement: Additional file 2 — Scores in 10,000 bp ENCODE region. A comparison of KuLCons, phastCons, GERP and SCONE scores in a 10,000 bp ENCODE region (hg17, ENm005, Chr21:32668244-32678960). [file 1471-2105-9-190-S2.pdf]

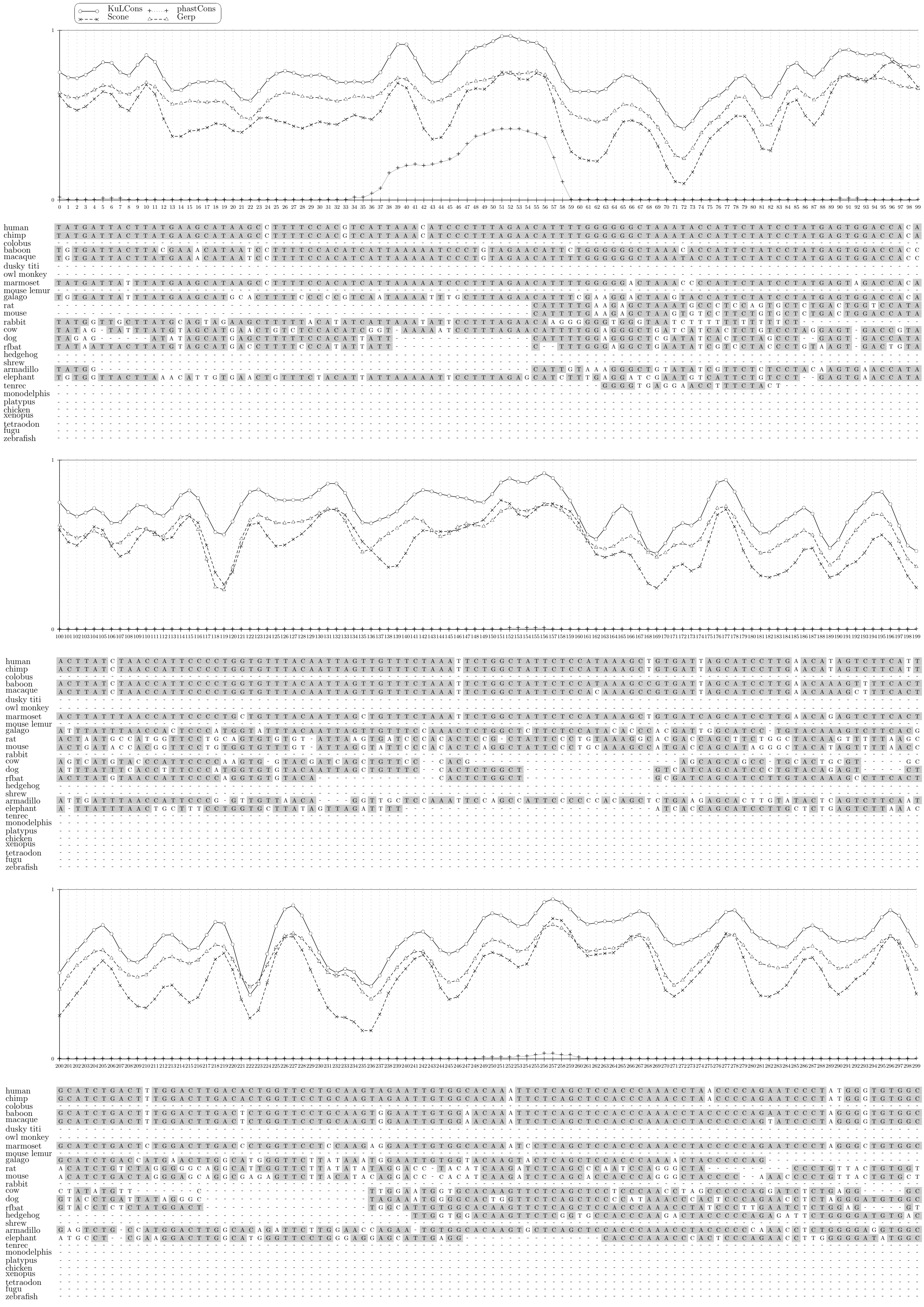
KuLCons
Scone
phastCons
Gerp
human
chimp
colobus
baboon
macaque
dusky titi
owl monkey
marmoset
mouse lemur
galago
rat
mouse
rabbit
cow
dog
rfbat
hedgehog
shrew
armadillo
elephant
tenrec
monodelphis
platypus
chicken
xenopus
tetraodon
fugu
zebrafish

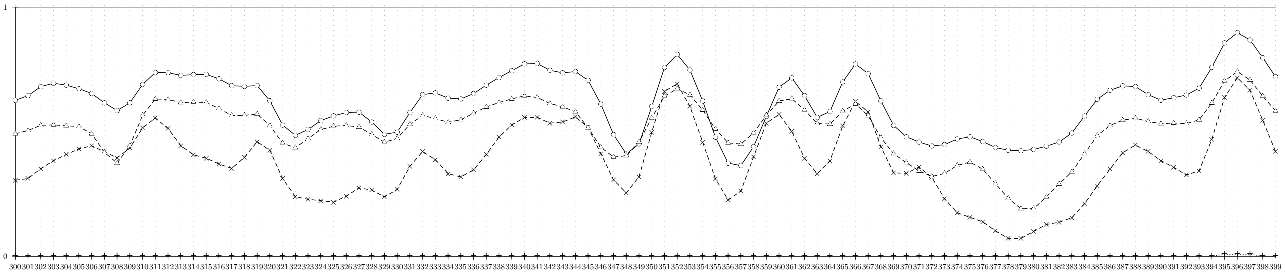

human	CCAGGTATATATATTTCAGATACATTCCCCTAGAGGTTCTGATGCAACTCCCCCAGTTCAGAACAACCATACGTCACGGTCCATGAACATATTTAAGACC
chimp	CCAGGTATATATATTTCAGATACATTCCCCTAGAGGTTCTGATGCAACTCCCCCAGTTCAGAACAACCATACGTCACAGTCCATGAACATATTTAAGACC
colobus	--
baboon	GCAGGTATATATATTTCAAATACATTCCCCTAGTGGTTCTGATGCAACTCCCCCAGTTCAGAACCACCATACGTCAGGGTCCATGAACATATTTAAGACC
macaque	CCAGGTATATATATTTCAAATACATTCCCCTAGTGGTTCTGATGCAACTCCCCCAGTTCAGAACCACCATACATCAGGGTCCATGAACATATTTAAGACC
dusky titi	--
owl monkey	--
marmoset	CCAGGTATACATATTTCAAATACATT-CCCTAGCAGTTCCGATGCAACCCT--------------------CGTCGGGGTCCATGACACTATTTAAGACC
mouse lemur	--
galago	--TTCAGAATAACCCTGCCTCAGGGTGCACGAA-ATATTTAAGACC
rat	TCAGGAAATTGTATTCCAAATGCATC-CTTGGGTGGTTTTAATACAA-----------TGAACCCACCGTAAGCTCAAACATGTAAATACACACCAGA-C
mouse	TCAGGAAATTGTATTCCAGA---------TGGGTGGTTCTAATGCAA-----------TGGAAT-------------AGCATGTAAGCATACATAAGA--
rabbit	--
cow	TTATGAGT---TTTTTCAAATCCATTCCCTGGGTGATCCTGAGGCGACTCCCCAAAC---------CCCCGCCTCCAGGTTGATACACATTTTCAAGACC
dog	CCAGATGTATGTGTTTCAAATTTGCTTCCTGGGTGATTCTGAGA--ACCTCCCAA--------------CACCCCCAAGGCTAGGAACATCTTTAAGACC
rfbat	TTATG------TGCCTCACATGAGTTTCCGAAGTGGTTCTGAGG--CCAGCCCCACCC-----CCACCCCACTCCCAAATCTACAAACATTTTTAAGACT
hedgehog	CCAAGTGTTGTGTTTTCAATATGCC-ATCAAGCTGCCC----------------------------CCTGCCCTCAAACGTGGGAGCATATTGAAGAAC
shrew	--
armadillo	CCAGGAGTCTATAATCC-------------------CTC---------------------------CCCTCTCCCCACAACGAGGAACAACTGTAAGC--
elephant	CCTGGTGTGTATATTTCTAAAACATT--CCTAGTGATTCCGACGCACGCTCCCCAGGGAGGAACAAGCACACAAAAAGGTCTGTGAACA--TTTCAGACA
tenrec	--
monodelphis	--
platypus	--
chicken	--
xenopus	--
tetraodon	--
fugu	--
zebrafish	--

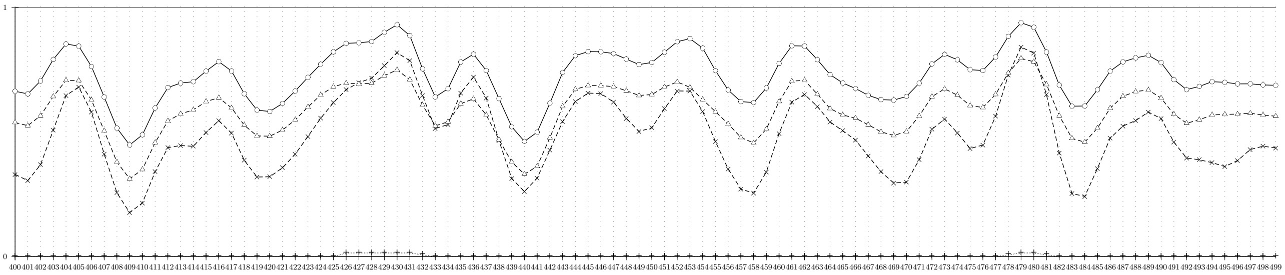

human	TCTGACACATCCTGTCAGAACTGTTATCTTCCATAGGGGATACAACACTTCCTCCTCAAGAAAGTATCTGATTCAAAGCATCCTTGCTAGCACTGAACAC
chimp	TCTGACACATCCTGTCAGAACTGTTATCTTCCATAGGGGATACAACACTTCCTCCTCAAGAAAGTATCTGATTCAAAGCATCCTTGCTAGCACTGAACAC
colobus	--
baboon	TCTGACACATCCTGTCAGAACTGTTATCTTCCATAGGGGATACAACACT-CCTTCTCAAGAAAGTATCTGATTCAAAGCATCCTGGCTAGCATTGAACAC
macaque	TCTGACACATCCTGTCAGAACTGTTATCTTCCATAGGGGATACAACACTTCCTTCTCAAGAAAGTATCTGATTCAAAGCATCCTGGCTAGCACTGAACAC
dusky titi	--
owl monkey	--
marmoset	TCTGACACATCCTGTCACAACAGTTATCTTCCACAGGGGATACAACACTTACTCCTCAACAAAGTATCTGACTCAGAACATCCCTGCTAGCACTGAACAC
mouse lemur	--
galago	TGTGACATACACTGCCAGCGACACTATCTTCCAAGGGGGCTGTAACGCTTCTTC--CAGGAAAGTATCTGATTCAAAGCATCTTTGCTAGCACTGAATAT
rat	TCTGACGCAGCATG-------------CCTC-----GAGGCTCAGCACTTTCTC--CAAGAAAGCCCTCACTTCTGCTCATCTTTGCTAG--CTGGGTAC
mouse	-CTGACACTTCATG-------------CGTC------CCGCTCAGCACGCTCTC--TCGGAAAGCGCTCACTTCTGTTCATCTTTGCTAGCACTGAGTAC
rabbit	--
cow	TCTGACACACACTGCCAGACCTGCTGTCTTCCAGAGGGGCCATAACACTTCCTC--CAGGGAAGTACCTGATTC--------TTTGCTGGTGCTGAGTAT
dog	TTTGACACATACTGCCAGACCCGCT----------------CTAACACTTCCTC--CACAAAAGTACCTGATTCAAAGCATCTCTGCTAGCAATGAATAT
rfbat	TTTGACACCTACTGCCAGGCCTGGT--CTTCCAGAGGGTT-GTAACACTTCCTC--CAGGAAAGCACTTGATTCAAAGCATCTTTCCTAGCACTAAATAT
hedgehog	ACACACACACTTCATCAGAGCTGCT-----CCATCGGGGCGCTGACCCCT------CTGGAAGATACTCGACTCAAAGCATCGTCACCGGCTTTGCACGT
shrew	--
armadillo	-----CGGGGTCTGT--------------------------GTAACACTTCCTC--CAGCAAAGCACCTGGCTCAAAGCATCTCGA--GGCACTG-----
elephant	TTTAACACATACTTCTAGAACTGCTGTCTTCCAGAGGGGCTGTAATACTT-CTC--TAAGAAAGTACCCACTTGAAAGCATCATT--TAGCATCAAATAG
tenrec	--
monodelphis	--
platypus	--
chicken	--
xenopus	--
tetraodon	--
fugu	--
zebrafish	--

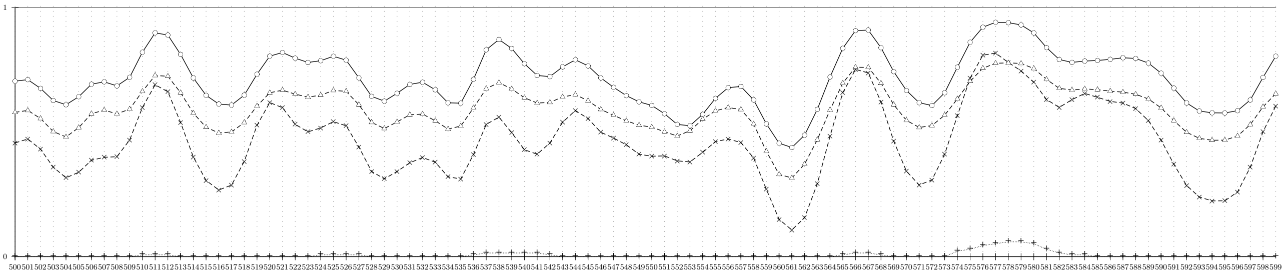

human	CATTGTTTTTAAAAGTCAATGCTATTTTAGTAGGCAAAAAAATCTGTATGTCATTATTTGCTTTACTTTAAATTAGGGTTTCTCAACCTCAGCTCTACTG
chimp	CATTGTTTTTAAAAGTCAATGCTATTTTAGTAGGCAAAAAAATCTGTATGTCATTATTTGCTTTACTTTAAATTAGGGTTTCTCAACCTCAGCTCTATTG
colobus	--
baboon	CATTGTTTTTAAAAGGCAATGCTATTTTAGTAGGCAAAAAAGTCTGTATGTCATTATTTGCATTACTTTAAATTAGGGTTTCTCAACCTCAGCTCCACTG
macaque	TATTGTTTTTAAAAGGCAATGCTATTTTAGTAGGCAAAAAAATCTGTATGTCACTATTTGCATTACTTTAAATTAGGGTTTCTCAACCTCAGCTCCACTG
dusky titi	--
owl monkey	--
marmoset	CATTGTTTTTAAAAGTCAGTGCTATTTTAGTGGGCAAAAAAATCTGTATGTCATCATTTGTATTACTTTAAATTAGGGTTTCTCAATCTCAGCTCTACTG
mouse lemur	--
galago	CAATG-TTTTAAAATTCAATGCTTTTTC--TAGGTGTAAAAACCTGTACATCATTATTTGCATAACTTTAA-----GGTTTCTCAACCTCAGCTGTGTTG
rat	TACTG----------------------TACTAAGT----------------------TCATAGTACTTTAGGTTAGGGTTTCTCAACCTCTATCCTG---
mouse	CA-TG----------------------TGGTGAGC----------------------TCAGATTACTTTAGGTGAGGGTTTCCCAGCCTCTACTCTG---
rabbit	--
cow	CA--GGTCTGAAAAGTCAAGGCCAACTTGGTAGGTGAAAAAACCTGTATCTCATGACTTGCTCTGCTTTGAATCCAGGTTTTTCAATCTCAGGACTACTG
dog	CATTGTTTTAAAAGATGCATGTTAATTTGATAGGTGAAAAAACCTGTATCTCCTGACTTATATTACTTTTAGTCAGGGTTTCTTAACCCCAGCACAATTG
rfbat	CATTATTTT--AAAGTCAGTGCTAATTTGGTAGGGAAAAAAACCTGCATCTCGTTAATT-CACTATTTTAAACCAGGGTTTCTCAACCTCAGAACTACTG
hedgehog	CACTCATCTTAAAAGTTAAT--
shrew	--
armadillo	----TTTTGAAAAAGGCAGTGCTAATTTGGTAGGTGAAATCATCTGAATCCCGT---CTGTCTGACTTTGAATCAGGGATTCTCGACCG-GGCACCACTG
elephant	CATCATTTTAAAAAGGCGATGCTAATTTATCAAATAAAAAAATTTTTTTTTTTTTATTTACATCACTTTAATCCAGGGTTTCCCGAC------AGTACCG
tenrec	--
monodelphis	--
platypus	--
chicken	--
xenopus	--
tetraodon	--
fugu	--
zebrafish	--

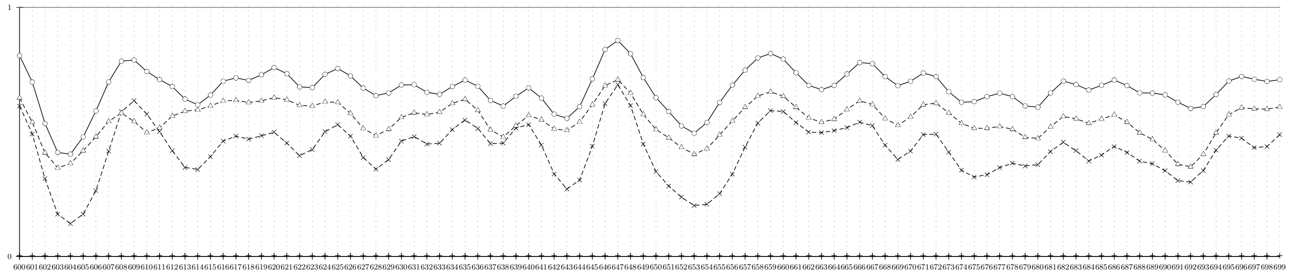

human	ACACTTGGGGCCAGGTAATTGTTATGGGGGCCATCCTGGGCATTACAGGATGTTCGGCAGCATCCCTGCTGTCTCCCCACTAGGCACCAGTTGTACCTCC
chimp	ACACTTGGGGCCAGGTAATTGTTATGGGGGCCATCCTGGGCATTACAGGATGTTCGGCAGCATCCCTGCTGTCTCCCCACTAGGCACCAGTTGTACCTCC
colobus	--
baboon	ACACTTGGGGCCAGGTAATTGTTTTGGGGGCCATCCTGGGCATTGCAGGATGTTTGGCAGCATCCCTGCTGTCTCCTCACTAGGCACCTGTTGTACCTCC
macaque	ACACTTGGGGCCAGGTAATTGTTTTGGGGGCCGTCCTGAGCATTGCAGGATGTCTGGCAGCATCCCTGCTGTCTCCTCACTAGGCACCCGTTGTACCTCC
dusky titi	--
owl monkey	--
marmoset	ACATTTGGGGCCAGACAATTGCTAT-GGGGCCGTCCTGGGCATT--AGGACATCTGGCAGCATCCCTGACGTCTATCCAC-------------TACCTTC
mouse lemur	--
galago	GCAAACGTGGT---------------GGGACTGTCCTGTGTGTGGCAGAATGTTTAGCAGCATCCCTGGTCTCTACCCACCAGATGCCAGTGGCACCTCC
rat	--------------------------GGAGCTGTCCTGA-------AGGATGTGTTCCAACATTGTTGGTCTC-ATCCACAAGGGACTAGTGGGACCATC
mouse	--------------------------GGAACTGCCCCGC-------AGGATGTGTTGTAACGTCACTGGTCTT-ATCCATAAGGGACTGTTGGTACCATG
rabbit	--
cow	ACATCTGGGGACAAATAACTGTTGTGGAGGCTGCCTTCTGCACTACAGGACGCTGAGCAGCCTCCTTGGTCTCTGCTCACCAGATGCCAGTCACATTTCT
dog	ACATTTTGGGCCAAATAATGGTTGTAGGGACTGTCTTGTGCAATGTAGGATGCTGAGCACCATCCCTGGCCTCTGCCCACTTG-TACCAGTTGTACTTCT
rfbat	ACCTTT-GGGACCAATGACTGTTGTGGGGGCTGTCCTGTGCATTGTAGGATGTGGAACAGCATTCCTGGTCTCCACCCACCAGATGCTAGTTGCACTTCC
hedgehog	---GCTGCCTGCATGCTACACGCCTGCTATAGCACC
shrew	--
armadillo	ACTGCCGTGG----------------ACTGCAGCTCTGT-------AGGCAACTGAGAAGCACCCCAGGCCTCCACCTCTGAGATGCCAGTTGCCCCTCC
elephant	ACCTTTTGGGCCAGGTAATTGTCATGGAGACTGCCCTACGCACTGTAGGGTGCCGAGCAGCAACCCTGGTCTCCACCCACTAGATGC-AGCTGCACCTC-
tenrec	--
monodelphis	--
platypus	--
chicken	--
xenopus	--
tetraodon	--
fugu	--
zebrafish	--

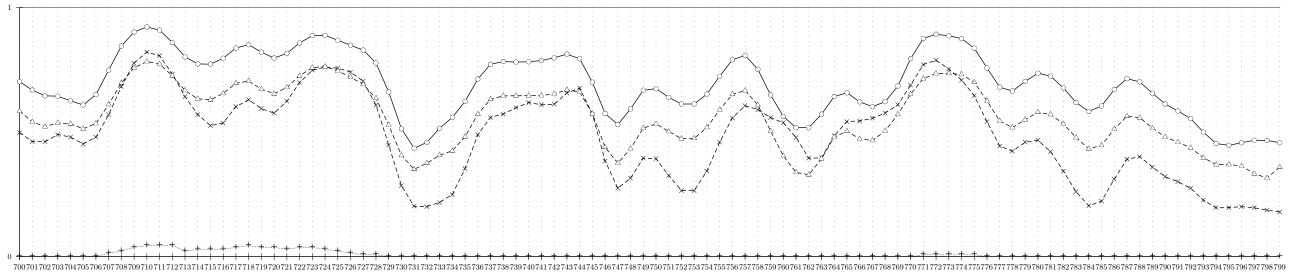

human	CCAGTTGTGACAATGCAAAATGTCTTCAGAGATTGACAAATGTCCCGTAGAAGGCAAAATTGCCCCCATTTGAGAATCACTCTTTAAAATACTTCGAAAA
chimp	CCAGTTGTGACAATGCAAAATGTCTTCAGAGATTGACAAATGTCTCGTAGAAGGCAAAATTGCCCCCAGTTGAGAATCACTCTTTAAAATACTTCGAAAA
colobus	--
baboon	CCAGTTGTGACAACCCAAAATGTCTTCAGAGATTGCCAAATGTCCCGTAGAAG-TAAAATTGCCCCCAGTTGAGAATCGCTCTTTAAAATACTTTGAAAA
macaque	CCAGTTGTGACAACCCAAAATGTCTTCAGAGTTTGCCAAATGTCCCGTAGAAGGCAAAATTGCCCCCAGTTGAGAATCGCTCTTTAAAATACTTTGAACA
dusky titi	--
owl monkey	--
marmoset	CCAGTTGTGA-----CAAAATGTCTTCAGAGATTTCCACACATACCGTAGAAGGCAAAATTGCCCCCA--------------------------------
mouse lemur	--
galago	CTGGCTGTGACAACCAAAAATGTCTTCAGACTTTGCCGAATGTCCCAGGGAAAGTATAATCACTCCTGGGTGAGAATCACTGTTTTTAATACTAAGAAAG
rat	CCA-CCATGACAATCAAAACTATCTCCAGATGTCTTCAAGTGTCCCCTAGAGGAGAAAGTCACCCTTA----AGAATCGCTGACATAAACACT----ACC
mouse	CCA-CCGTGACAATCAGAACTATCTCCAGACACCTTCAAGTGTCCCCTAGCGGAGAAAGTCACCCTGAG---AGAATCCCTGATGTAAACACT----ACC
rabbit	--AAAATAAATCTTTAAAAAAGTCCGAAC-
cow	CCGGTTATGACAACCAAAACT------------TGCCAGATGGCCCGTGGGGGACAGAACTGTGCCCAGCTGAGAA-----GCTCTAGATGCTATGTGC-
dog	CCAGTCATGACAACCAAAAATGTTTCCAGGTGCTTTCAAATGTCCCGAGGGGCACAAAATCACTCCCAGCTGAGAACCACAGTTTGAAATATTATG--C-
rfbat	CCAGTTCTGACAACCAAAAATGTCTCCAGACATTGCCAAATGTCCCATGGGGAGCAAAAGCGTCCCCTGTTGAGAATCACTGTTTTAAATATAATATGC-
hedgehog	CTCGTGGTGACAACCAGAAA-ATCTCTGGAC-------AACGCCCCAGGGTGGACGAAAGCAAGCCAAGCTGCGCACTGCT-TTCTGAGTGTTCTGGT--
shrew	--
armadillo	CAG--TGTGACAATCAAAAA-GTCTCCACACATGGCC-AATGTCCCGGAGGGGGCAAAATCGCCTCCGG--GAGAACCACTGTTTTAAGCAGTAGGCA-G
elephant	CCAGCTGATATAACCATAAAGGTCTCCAGATGTTGTCAAATGTCCCACGGGAGGCAAAATTGGCCCCGGGTGAGAACCACTGTCTT-AATACTACTGAAG
tenrec	--
monodelphis	--
platypus	--
chicken	--
xenopus	--
tetraodon	--
fugu	--
zebrafish	--

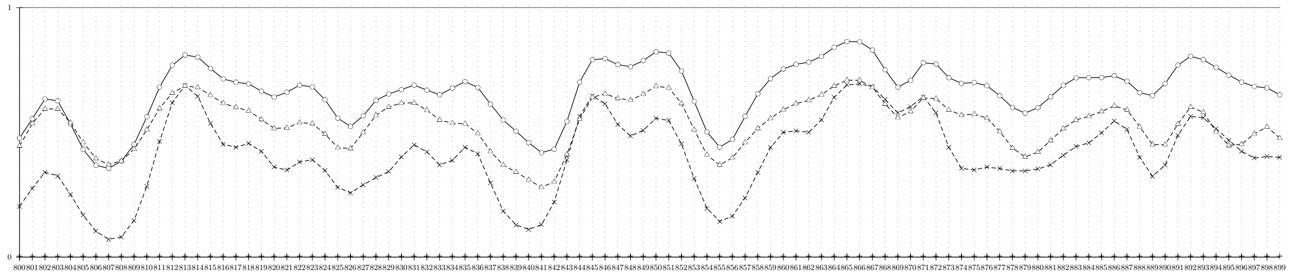

human	TAAAGTCCTTTCATGCTTATTAGCCATTTTCATGTGGACTCTGGACAACTTGTCATGTTGGGAGGGTCATAGTTTTTCTTACTGATGATTTAAAGTGTTT
chimp	TAAAGTCCTTTCATGCTTATTAGCCATTTTCATGAGGCCTCTGGACAACTTGTCATGTTGGGAGGGTCATAGTTTTTCTTACTGATGATTTAAAGTGTTT
colobus	--
baboon	TAAAGTCCTTTCATGCTTATCAGCCACTTTCATGTGGACTTTGGACAACATGTCATGTCGGGAGGGTCACAGTTTTTCTTACTGATGATTTAAGGTGTTT
macaque	TAAAGTCCTTTCATGCTTATTAGCCACTTTCATGTGGACTCTGGACAACATGTCATGTCGGGAGGGTCACAGTTTTTCTTACTGCTGATTTAAGGTGTTT
dusky titi	--
owl monkey	--
marmoset	--TGTTTCTTACTGATGATCTAAGGTGTT-
mouse lemur	--
galago	AAAAGCATTTTCATGTTTATCAGTCATGTTTATCTGAGCTTTTCACAACTTTTCAGGTTGAGAGGGTC----TTTTTCTAACCGATCATTTAAGGGGCTT
rat	TGATATTCAGCCATGCTTCTTAGCCTGTTTTAAATGGATTCT----AACTTTTCAT-TTCAGAGGGTCAG---TTTGTTCACTGACCACTGAAAGTATCT
mouse	TGATGTCCATCCGTGCTTCTTAGTCTTTTGTAAATGGATTCT----AACTTTTCAT-TTCAGGGGGTCAG---TTTGTTCACTGATCACTGAAGGTATCT
rabbit	---------------------------------ATGGACTTCCAACAACTTTACTG--CGGGAAGG-------GTTTCTAACTGCTCACTTCAGGTGCTT
cow	----------------TCATGAGCCCCT------CAGCCTTTGGACAACTTTTTATTATGGGAGGGTCACAGTTTTTCTCATGGATCACAGAAGGGGCTT
dog	------------ATGCTAACTAGCTATT------TGGTTTTC-----ACTTTTTCTACTGGGAGGGTCATAGTTTTTCTAACTGATCATTTAAGTTGCTT
rfbat	-------ATACTATGCTAATTAGCCATT------TGGCCTTTTGACAATTTTTTATATTGGAAGGGTCACAGTTTTTTT------------AAGGGGCTT
hedgehog	-------------------TAGGCCCCT-------GGCCTCTGGACGGCTTTTCTGGTTGGGAGAGGCACAG--ACTTCTACAGTTCACTGAAGGTGACT
shrew	--
armadillo	GGAAACATGATCACGTTTATTTGCCACTGTCAT-T-GCACCACCAGAAT-------------------ATGGTCCCTTTCGGTGACCGTTTAAGGTGCTT
elephant	TCAAACATTTTCATGTCTATTACACATTTTTAT-TTGCTATTTGACAGCCTTTTGGATTTGGGAGGCCAGAGTTTTTCTAATTGAC-GTTTAAAGTGTTT
tenrec	--
monodelphis	--
platypus	--
chicken	--
xenopus	--
tetraodon	--
fugu	--
zebrafish	--

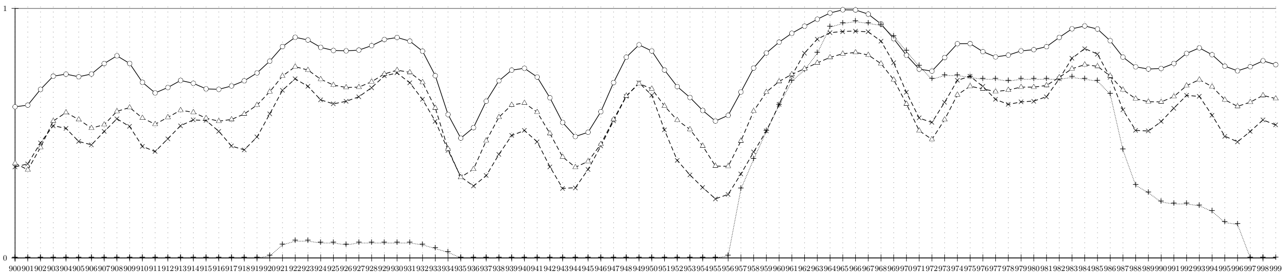

human	CTGTATTAAAGGCAGTGATCTTTTTAGCAAGTTTTAAAATATCAATAAGATGCTTGCAAAAGGCAGAATATGGAACATGACACAGAACAACATTTTGCTA
chimp	CTGTATTAAAGGCAGTGATCTTTTTAGCAAGTTTTAAAATATCAATAAGATGCTTGCAAAAGGCAGAATATGGAACATGACACAAAACAACATTTTGCTA
colobus	--
baboon	CTGTGTTAAAGGCAGTGATCCTTTTAGCAAGTTTTCAAATATGGATGAGATGCTTGCAAAAGGCAGAATATGGAACATGACACAAAACAACCTTTTGCTA
macaque	CTGTGTTAAAGGCAGTGATCCTTTTAGCAAGTTTTCAAATATGGATGAGATGCTTGCAAAAGGCAGAATATGGAACATGACACAAAACAACCTTTTGCTA
dusky titi	--
owl monkey	--
marmoset	------------------TCCTTTCAGCAAGTTTTACAATATTGCTCAGATGCTCACAAAAGGCAGAATATGGAACATGACACAAAACAACATTTTGCTA
mouse lemur	--
galago	CTGTAGTA-----AGTGATCCTTTTAGCAAGTCTTCAAATTTCTGTAAGATTTTTGTAAGAGGCAGAATATGGAACATGACACAAAATAGTATTCTATTA
rat	GCATATAAAATCC-ATGATCCTTTTACCAAGTTTAAAAATGTCTCTTAGATAACTGAAAAGGGCAGAATATGCAACATGACTC-AAATAACACTTGGGCA
mouse	--ATATACAATTC-GTGATCCTTTTACCAAGTTT-TAAATGTTTCTTAGATATCTAAAAAAGGCAGAATATGCAACATGACTC-AAATAACACTTGGGCA
rabbit	CTGTATTCAAGTCAGTGGCCCTTTCAGCAAATTT-TAAGTTTCTATGA------------------AATATGGA----------AAATGACATTTTGCTA
cow	CTGTGTCAAAGACAGCGATCCTTTCAGCAAGTTTTCAGATTTCCGCAAGATGCTTATGTAAGGCAGAATATGGAAGATGACACAAAATGACATTTTGCTA
dog	TTGTATTAAAGACAGCAATTCCTTCAGCCAGTTCTAAAATTTCTACCAGATGCTTGTAAAAGGCAGAATATGTAAGGTAATTCAAAATAATGTTTTGCTA
rfbat	GGGTATTAAAAACAGTGATCCTTTCAGCAAGTTTTTAAATTTCTATAATATGCTTGTAAAAGACAGAATGTACAAGATAACACAAAACAACATTTCGCTA
hedgehog	TGTCATTAAAGAC------TTTTTCAATGAGTTTTAAATTTTC---
shrew	--
armadillo	CTGTATTAAAGGTGATTCCCCTTTCAGCAAGCTTTGCGGTTTCTCTAAGATGCTTGG----------------------ACACAAAATAACATTTTACTA
elephant	TTGTACTAAGGACAGGGATCCTTTCAGCAAGTTTTCAAATCTCTATAAAATGCTCAGAAAAGGCAGAATAGGCAACACGACACAA---AACATTTTGCTA
tenrec	--
monodelphis	--
platypus	--
chicken	--
xenopus	--
tetraodon	--
fugu	--
zebrafish	--

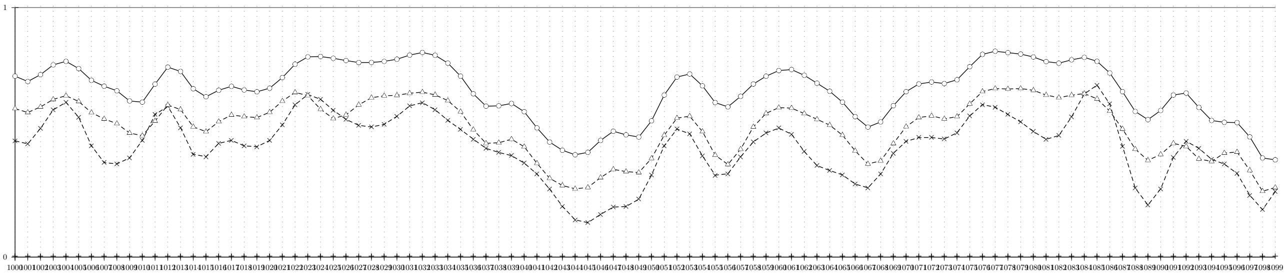

human	GATATGTGAATGAAGTATTATTTATAATTTACAGCTCACAATGATTACTTTGGGTTGCTGTGAAATGTTGACCAGCAGCTTTTCTGCTGCTGTAATAAAA
chimp	GATATGTGAATGAAGTATTATTTATAATTTACAGCTCACAATGATTACTTTGGGTTGCTGTGAAATGTTGACCAGCAGCTTTTCTGCTGCTGTAATAAAA
colobus	--
baboon	GCTATGTGAATGAAGTATT---TATAATTGACAGCCCATGATGATCACTGTGGGTTGCTGTGAAATGCTGACCATCAGCTTTTCTGCTGCTGTAATAAAA
macaque	GCTATGTGAATGAAGTATTATTTATAATTGACAGCCCACGATGATCACTTTGGGTTGCTGTGAAATGTTGACCATCAGCTTTTCTGCTGCTGTAATAAAA
dusky titi	--
owl monkey	--
marmoset	AATATGTGAATGAAGCATTATTTATAATTTACAGCTCACAATTATTACTTCGGGTTGCTGTGAACTGTGGACCAGCAGCTTTGCTGCTGCTGTAAC-ACA
mouse lemur	--
galago	GATGTGTAAGTTAACTATTATTCATAACT-ACACCTCACACTTACTACACCAAGTTACTGTGAAATATTGACCAATAACTTTCCTGCAACGCTAATAGAG
rat	TGTCTGT----AAAGTATCACTTGTAAGTTACAGTCCGAAATACTGCAGCTGAGTCCCCATGGACTGCAGGCTGACAGCTTTTCTCT--CAGTAGCAAAA
mouse	GGTTTGTAAAGAAAGTATCATTTGCAATTTACAGTCCAAACTGCTGCAGCTGAGGTCATA------------TGACAGCTTTTCCCTTATAGTCGCAAAG
rabbit	GGTGTGACAAGGAAGAAGTATTTGTAACTTACAGCTCCCAGTCACCCCACTGGGCAACTGCGCAACGTCAACCAGC-GCTTTTATCG--CTGTTGC----
cow	GGTGTGTACGGGAAGAA-GGGCTGTCATTTGCAGCTCGCAGTCACCCCAGTGGCTTCCTGTGGAATGGGGGGTAGCAGGTCTTCTCCTCTTGTAAGAA-G
dog	AGTGTGTAAATGAAAAA-CAGCTGTAATTTACAGCTTTCAATTAATCCAA-GGCTTCCCGTGAAATAGTG---AGCAA-----CTCCTGGTGTGATAATA
rfbat	GGTGTGCAAATGAAGGA-CAGTTGTCATTTATAGCTCACAATGACTCCCATGGCTTCCTATGAAAGGGTGACCAGCAGCTTTTCTCCTGCTGTGAGAG-A
hedgehog	--
shrew	--
armadillo	GATGTACAAACGAGA---TAATTGTAATTCAGAGCTCACAACTGTTACATAAGCTTACTGT-------TGAC---CAGCTTTTCTCCTGTAATGACAGGC
elephant	GATGTATAAAAGAAGTATTATTTGTAATTTACACCTCGCAATTATCGTACTGGCTTTCTGGGAGATGCTGGCCAACAGCCTGTCTCCTGCTGT-ATAACA
tenrec	--
monodelphis	--
platypus	--
chicken	--
xenopus	--
tetraodon	--
fugu	--
zebrafish	--

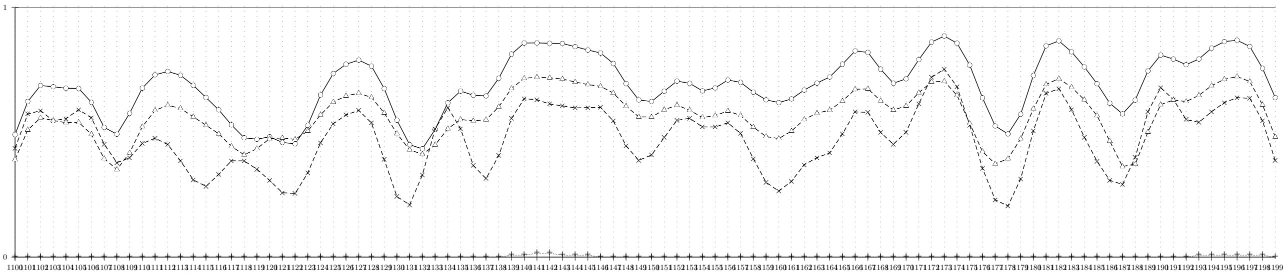

human	CAGCAGCCATGTCTGGACATCACTGGCTGCTTCAGCTATTCTTTAACCTTCTAGTCCCAGGATATGCTCATCAACAAGAACAAGTTTCTGCTTAGGGAAG
chimp	CAGCAGCCATGTCTGGACATCACTGGCTGCTTCAGCTATTCTTTAACCTTCTAGCCCCAGGATATGCTCATCAACAAGAACAAGTTTCTGCTTAGGGAAG
colobus	--
baboon	CAGCAGCCACGTCTGGACATCACTGGCTGCTTCAGCTATTCCTTAACCTTCTAGTCTCGGGATATGCTCATCAACAAGAACAAATTTCTGCTTAAGGAAG
macaque	CAGCAGCCACGTCTGGACGTCACTGGCTGCTTCAGCTATTCCTTAACCTTCTAGTCTCGGGGTATGCTCATCAACAAGAACAAGTTTCTGCTTAAGGAAG
dusky titi	--
owl monkey	--
marmoset	CAGCGGCCATGTCTGGACATCACCGGCTGCTTCAGCTATTCTTTAACCTGCCAGTCCCAGCATATGCTCATCAACAAGAACAAGTTTCTGCTTAAGGAAG
mouse lemur	--
galago	----------GCCTGAACACCAGGGGTTGCTCTCCTTACTCTTTAGCCTTCTAGTC-CGGGATTTGCTTATCACCAAGAACAAATTTCAGCTT----AAA
rat	GGATCAGGCTGCTTGA------------GCTCTAGCTCTTCCTTAACCCCGCAGCCCCAAAACTAACTCCCCACCAATCCAAAGTGTAAGCCT----AAG
mouse	CGGCCAGGCTGCTTGA------------GTTCCCGCTCTTCTTTAACCTTCCAGCCC-AAACTAACTCCCCACCAATGCCAAGTTTAAGCCTAAGTAAG
rabbit	----TGGGATG--GAA------------GCAGCCGCCAGTCTTCAGCCTCCCGGGCCCAGGCTTTGCTCACCACCCCGAGCAAGCCTGGGCGTAAGAAAG
cow	TGGCAGCTGTAAC---------------------------CTT--------------CAGGAT-CGCTCACCACCA---CCAAGTTCTTGCTTAAGAAAC
dog	TGGAAGC---CTTAAGAAA-
rfbat	AGGCAGCCCTGCCTCAGCCTAGGTGGCCG-----------CTT--------------CAGGAT-TGCTTGTCACCA---CCAAGTTTCTGCTGAAGAAAT
hedgehog	--
shrew	--
armadillo	TGGCAGCTTGGCCTGAACTTAAGTGACTGCTTTAGCTCTTCTTTAACTTTCTAGTTCACAGGT------------------AAGTTTCTGCTCAAGA--A
elephant	TGGCAGCCTTGCCTGCCCGTACACGGCTGTCCTAGCAATTCTT-AACCTTCTAGCCCCAGAATTTCCTCACTACCACAGGCAAGTTTTGGCTTCAGAAAC
tenrec	--
monodelphis	---AAG
platypus	--
chicken	--
xenopus	--
tetraodon	--
fugu	--
zebrafish	--

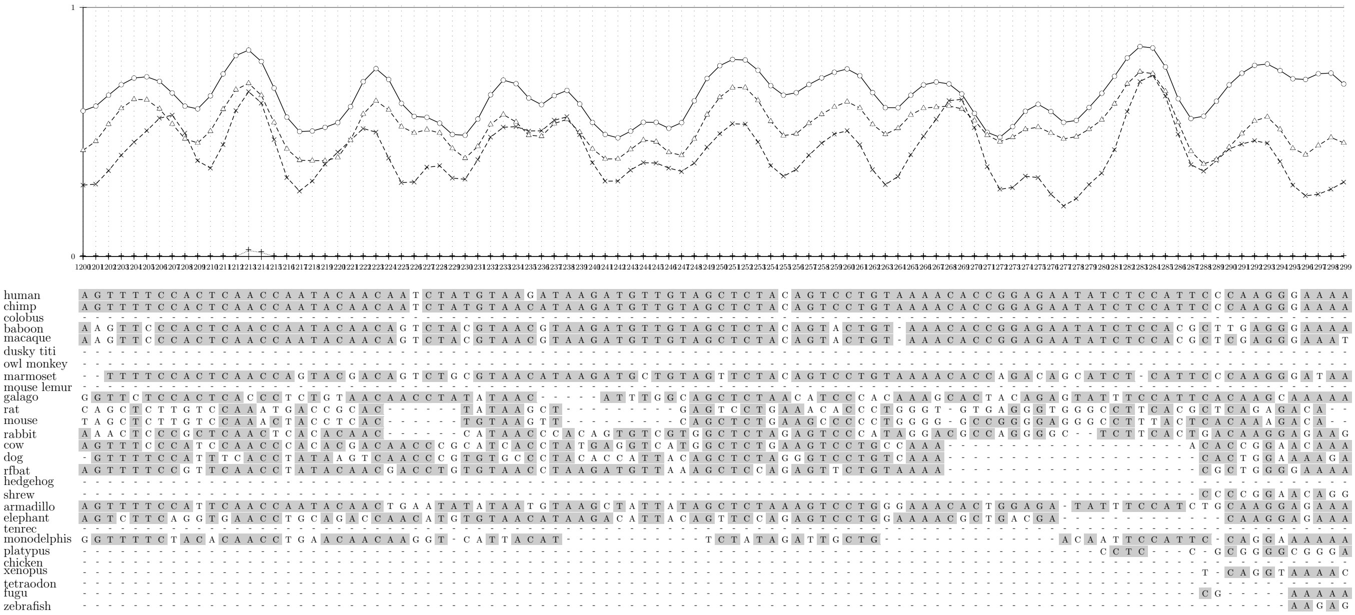

1
0
human
chimp
colobus
baboon
macaque
dusky titi
owl monkey
marmoset
mouse lemur
galago
rat
mouse
rabbit
cow
dog
rfbat
hedgehog
shrew
armadillo
elephant
tenrec
monodelphis
platypus
chicken
xenopus
tetraodon
fugu
zebrafish

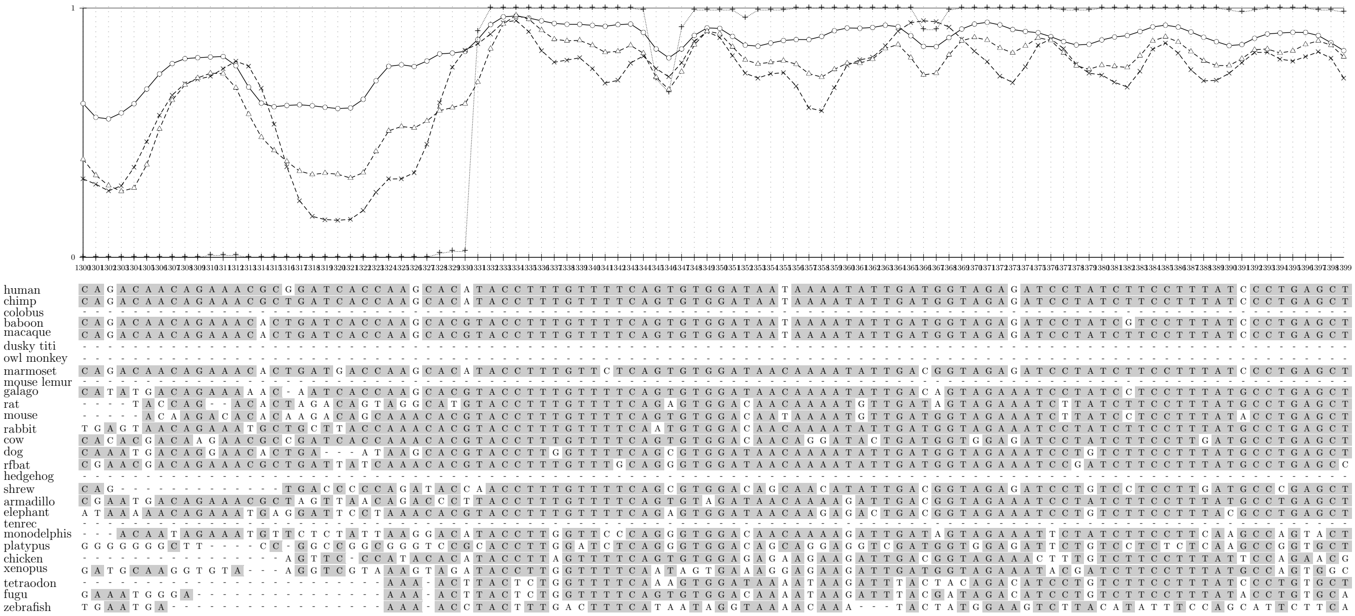

1
0
human
chimp
colobus
baboon
macaque
dusky titi
owl monkey
marmoset
mouse lemur
galago
rat
mouse
rabbit
cow
dog
rfbat
hedgehog
shrew
armadillo
elephant
tenrec
monodelphis
platypus
chicken
xenopus
tetraodon
fugu
zebrafish

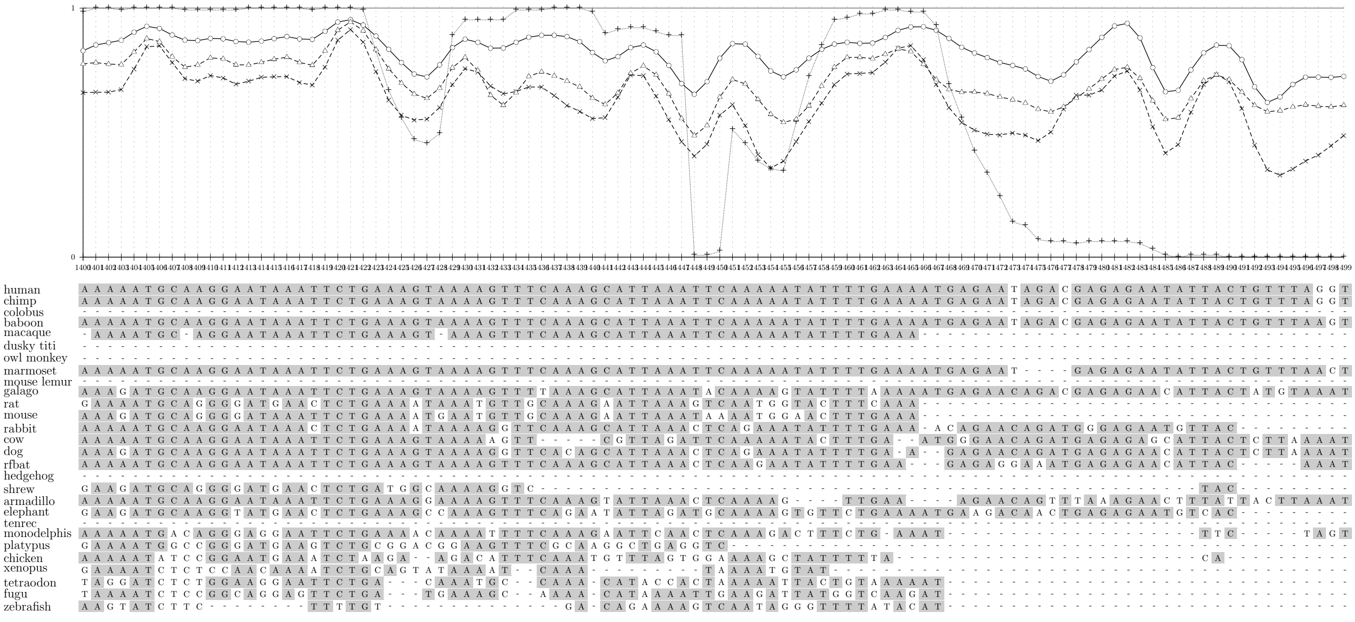

1
0
human
chimp
colobus
baboon
macaque
dusky titi
owl monkey
marmoset
mouse lemur
galago
rat
mouse
rabbit
cow
dog
rfbat
hedgehog
shrew
armadillo
elephant
tenrec
monodelphis
platypus
chicken
xenopus
tetraodon
fugu
zebrafish

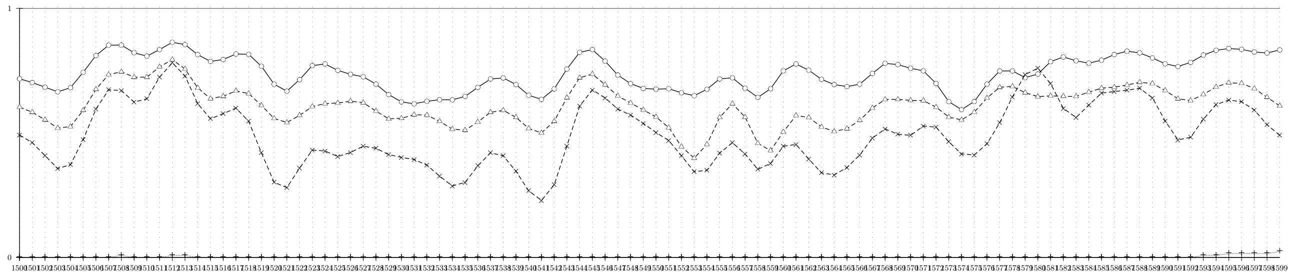

human	GGTACAGCAAACAATGCCATTTACTTTTTCAAGTTTTTTTTTTTTTAACTATACAAGTTTCCGGTAGAAATAACCACTTCCATGACATAAGAAATCAAGAA
chimp	GGTACAGCAAACAATGCCATTTACTTTTTCAAGTTTTTTTTTTTTTAA-TATACAAGTTTCCGGTAGAAATAACCACTTCCATGACATAAGAAATCAAGAA
colobus	--
baboon	AGTACAGCAAACAATGCCTTTTACCTTTTCAAGTTTTTTTTTTTTTAACTACACAAGTTTCCAGTAGACATAACCACTTCCATGACATAAGAAATCAAGAA
macaque	--
dusky titi	--
owl monkey	--
marmoset	GTTACAGCAAACAATGCTATTCACCTTTTTAG--TTGTTTTTTTTAACTATACCAGTTCTCAGTAGGAATAACCACTTCCGTGACATAAGAAATCAAGAA
mouse lemur	--
galago	GGTACAACAAACAACTCCACTTGCCTTTTTAAACTTTTCTTTTTTAGCTATACAAATTTGCAGTAG----------------GACATAAGAAATCAAGAA
rat	TGTAGAGCAAATAACGCCAC------CCCG----ATTTCTGCACCAATTATACAAGTTTTCAACAGTAA--ACCGGCTCCATGGCACAGGGAGTCAAGG-
mouse	TGTAGAGCAAATAATGCCAC-----ACCTG----ATTTCTGTGCCAGGTTCACAG--TTTCAACAGTAA--ACTGGTTCCGTGGCACAGGGACTCAAGA-
rabbit	TATTTAGCAAGTAATGT---------CTTT----TTTTCAGTTTTAATTATACAA--GTTCA--------------------------------------
cow	GGTACGGCAAACAATGCCATTGACTTTTTTAGGTTTGTCTTTTTTAATTACACAAGTTCACAGCAGGAAAAACTATTTTTGTGACATAGGAGATAAA---
dog	GGCGCAGCACACAATGCCATTTTCTTCTTTAGGTTTGTCTTTCTTAATTATGCAAGTTTACAGTAGGAATAACTACTT------CATAGGAAATCAG---
rfbat	GGTATAGCAAACAATGCCATCTACTTTTTCAAGTTTGTCTTTTTTAATTATACAAGTT--CAGTGGAAATGACCACTTTTGTGACAAAGGAAATCAA-A-
hedgehog	--
shrew	----------------------------------CGTTTTACTCTAATTTTATTTTTACACAGTTGGAATGATT--------------------------
armadillo	GGTACAGCAAACAATGGCATTTACTTTTTTAAGTTTTTTTGCTTTA-CTATATTAATTCTCACTTA-----------------AC---------------
elephant	-----AGCAATCAATGGC--ATACTTTTTA----AAGTCTTTTTTAATTATACGAATTCACA-------------TTTTTTTTTTAT-------------
tenrec	--
monodelphis	GATGAATTAGACAAAAGCATTCA---------------TTTCTTT-ATAAAATTAATTCGCT-------------TTCTTGT------------------
platypus	--
chicken	-CTCC-----TCCATGCCATTGACTTCTCT------------------CCCAAAAAACTGCAATAA----------------GAGTTAAAGGATG--A-A
xenopus	--
tetraodon	--
fugu	--
zebrafish	--

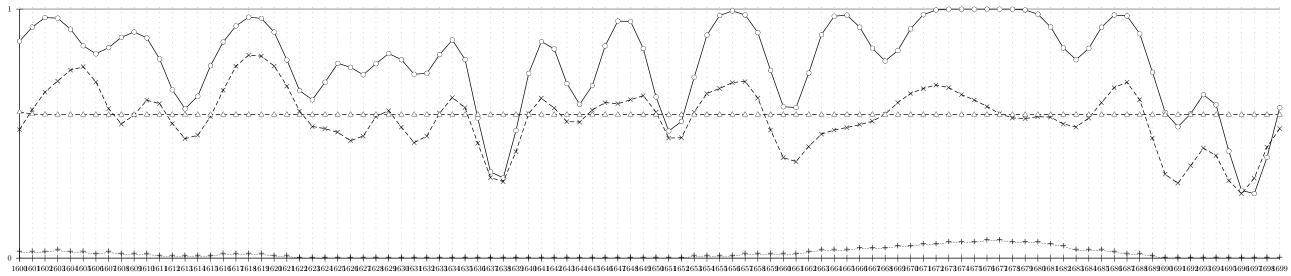

human	GCCAGGCAGAGTGGCTCATGCCTGTAAGCCCAGCACTACAGGAGGCCAAGGTGGGAGGATCCCTTGAGGCCAGGAGTTCAAGACCAGCCTGGGCAATATA
chimp	GCCAGGCAGAGTGGCTCATGCCTGTAAGCCCAGCACTACAGGAGGCCAAGGTGGGAGGATCCCTTGAGGCCAGGAGTTCAAGACCAGCCTGGGCAATATA
colobus	--
baboon	GCCAGGCACAGTGGCTCATGCCTGTAAACCCAGCACTATGGGAGGCCAAGGCGGGAGGATCCCTTGAGGCCAGGAGTTCAAGAACAGCCTGGGCAATATA
macaque	--
dusky titi	--
owl monkey	--
marmoset	GCCAGGC--AGTGTCTCATGCCTGTAAGCCCAGCACTTTGGGAGTCCAAGGTGGGAGGATTCCTTGAGGCC-----TTCAAGACCAGCCTTGGCAGTGTA
mouse lemur	--
galago	ACCAGGCACAGTGACTCATGCCTATAATCCTAACACTCTGGGAGACCAAGGCAGGAGGATCACTTGAGCCCAGGAGTTCAAGACCAGCCTGAACAAGT--
rat	--
mouse	--
rabbit	--
cow	--
dog	--
rfbat	--
hedgehog	--
shrew	--
armadillo	--
elephant	--
tenrec	--
monodelphis	--
platypus	--
chicken	ACCAGTGACAGCAAGTAATGCTGCCAAACC--
xenopus	--
tetraodon	--
fugu	--
zebrafish	--

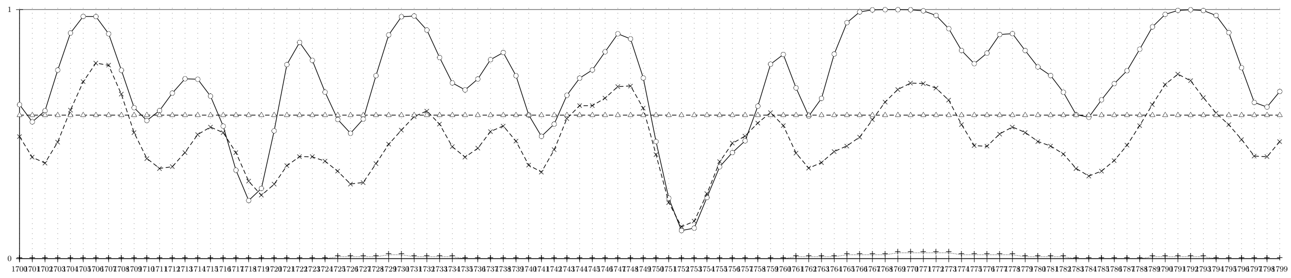

human	GCAAGACCCTGTCTCCAAAAAAAAATTTAAAAATTAGCTGGGTATGGTGGTGCGCACCTGTAGTCTTACTGTAGTCTTAGGTACCTAAGAGGCTGAGGTG
chimp	GCAAGACCCTGTCTCTAAAAAAAAATTTAAAAATTAGCTGGGTATGGTGGTGCACACCTGTAGTCTTACTGTAGTCTTAGGTACCTAAGAGGCTGAGGTG
colobus	--
baboon	GCAAGACCCTGTCTCTAAAAAAAAATTTAAAAATGATCTGGGTATGGTGGTGTGCACATGTACTCTTACTGTAGTCTTAGCTACCTAGGAGGCTGAGGTG
macaque	--
dusky titi	--
owl monkey	--
marmoset	GTAAGACCCCATCTCT-ATGAAAAGTTTAAAAATTAGCTGTTTATGGTGGTGCACACTTGTAGTCTTA---TAGTCTTAGCTACCTAGGAGGCTGAGGTG
mouse lemur	--
galago	GTGAGACCCTGTTTCT-ACTAAAAATAGAAAAATTAGCTGGGCATTGTGGCCGGTGCCTG-----------TAGTTTTAGCTAGTT-GGAGGCTGAGCAG
rat	--
mouse	--
rabbit	--
cow	--
dog	--
rfbat	--
hedgehog	--
shrew	--
armadillo	--
elephant	--
tenrec	--
monodelphis	--
platypus	--
chicken	--
xenopus	--
tetraodon	--
fugu	--
zebrafish	--

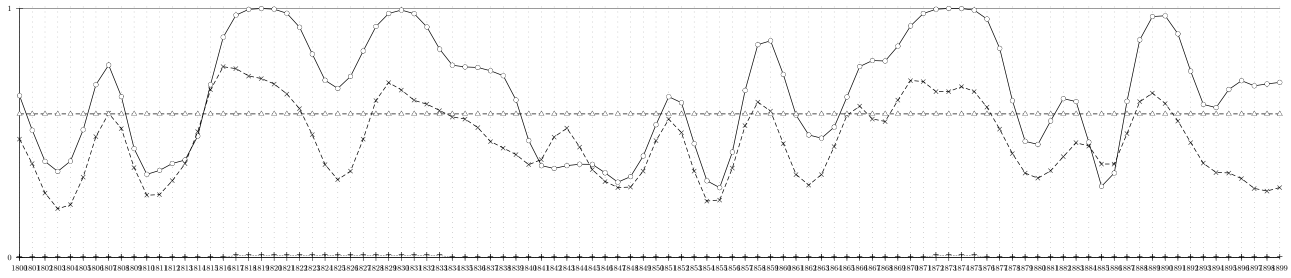

```
human        GCAGGATCACCAGTACCCAGGAGTTTGAGGTTGCAGTAAGCCATGATCGCACCACTGCACTCCAGCCTAGGCAACAGAGCAAGACGCTGTCTCCAAAAAA
chimp        GCAGGATCACCAGTACCCAGGAGTTTGAGGTTGCAGTAAGCCATGATCGCACCACTGCACTCCAGCCTAGGCAACAGAGCAAGACGCTGTCTCCAAAAAA
colobus      ----------------------------------------------------------------------------------------------------
baboon       GCAGGATCAGTAGTACCCAGGAGTTTGAGGTTGCAGTAAGCTATGATTGCACCACCACACTCCAACCTAGGCAACAGAGAAAGACAGTGTCTCCAAAAAA
macaque      ----------------------------------------------------------------------------------------------------
dusky titi   ----------------------------------------------------------------------------------------------------
owl monkey   ----------------------------------------------------------------------------------------------------
marmoset     GCAAGATCACTAGCACCCAGGAGTTTGAGGTTGCAGTAAGTCACGATCGCACCACTGCACTCCAACC-AGGC-ACAGAGCAAAACCCTGTCTCCAAACAA
mouse lemur  ----------------------------------------------------------------------------------------------------
galago       GAGAATTCACCGGAGCCCAGGAGTCTAAGGTTGCTGTGAGCTACACTAATGCCTTAGCACTGTAGCCTGGGCAACAGAGTGAGAC--TGTCTCAGAAA--
rat          ----------------------------------------------------------------------------------------------------
mouse        ----------------------------------------------------------------------------------------------------
rabbit       ----------------------------------------------------------------------------------------------------
cow          ----------------------------------------------------------------------------------------------------
dog          ----------------------------------------------------------------------------------------------------
rfbat        ----------------------------------------------------------------------------------------------------
hedgehog     ----------------------------------------------------------------------------------------------------
shrew        ----------------------------------------------------------------------------------------------------
armadillo    ----------------------------------------------------------------------------------------------------
elephant     ----------------------------------------------------------------------------------------------------
tenrec       ----------------------------------------------------------------------------------------------------
monodelphis  ----------------------------------------------------------------------------------------------------
platypus     ----------------------------------------------------------------------------------------------------
chicken      ----------------------------------------------------------------------------------------------------
xenopus      ----------------------------------------------------------------------------------------------------
tetraodon    ----------------------------------------------------------------------------------------------------
fugu         ----------------------------------------------------------------------------------------------------
zebrafish    ----------------------------------------------------------------------------------------------------
```

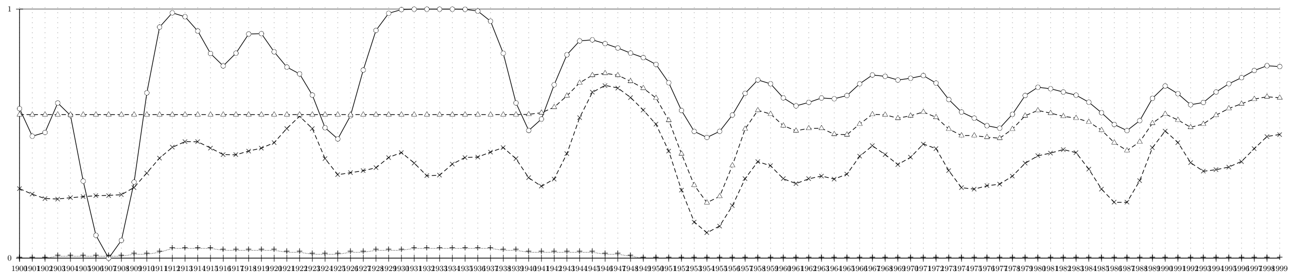

```
human        ACAAAACAAAACAAAACAAAAAGGAAGAAAGAAAAGAAATCAAGAGCACCTACAAGCGAACCAATGACAGAACTTTTTAAGAAGTTCAGTAGGTCTTTAG
chimp        AAAAAAAACAA--AAACAAAAAGGAAGAAAGAAAAGAAATCAAGAGCACCTACAAGCGAACCAATGACAGAACTTTTTAAGAAGCTCAGTAGGTCTTTAG
colobus      ----------------------------------------------------------------------------------------------------
baboon       ACAAAACAAAACAAAACAAAAAGGAGGAAAGAAAAGAAATCAAGAGCACCTAAAGGTGAACCAATGACAGAACTTTTTAAGAAGTTCAGTAGGTCTTTAG
macaque      ----------------------------------------------------------------------------------------------------
dusky titi   ----------------------------------------------------------------------------------------------------
owl monkey   ----------------------------------------------------------------------------------------------------
marmoset     AC-----------AAACAAAACGGAAGAAAG--AAGAAATGAAAAGCACCTAATGGTGAACCAGTGACAGAACTCTTAAAGAAGTTCAGTAGGTCTTTAG
mouse lemur  ----------------------------------------------------------------------------------------------------
galago       -------------AAAAAAAAAGGGGG------------------GTACCTAAAAGTG-CCTAATGACAGAACTTTTAAAGAAGTTTGGTA-CTTTTTAG
rat          ----------------------------------------------CATCTCAGGGTGAAATGACCACGGAA--------------------------AT
mouse        ----------------------------------------------CATCTCAAGGTAAAACAATCACAGAACTTTCAGGAAGGG-TGGTAGTTGCCCAG
rabbit       ----------------------------------------------TACTTAAGCATGAAGCAAAATCAATACTGACTGAAAG-----------------
cow          -----------------------------------------------ATCTAAAAGCAATACAATGAGTGAACCACTCAAGTT---CAACAGTTCTTTGG
dog          -----------------------------------------------ATCTAAAGGTAAAACAATAACAGAACCATTAAAGTTGTTCATTAGTTCTGT--
rfbat        -----------------------------------------------ATCTACAGCTAAAATAATGACAGGAAGATTAAAGTTGTTCCATAGCTCTTTAG
hedgehog     ----------------------------------------------------------------------------------------------------
shrew        ----------------------------------------------------------------------------------------------------
armadillo    --------------------------------------------AGTACATAAAGTAGAAACAATGACAGAATTTTTTAAAACGCTCAGTCATTCTTTAG
elephant     --------------------------------------------AGTACCTACGGCTAAAAAAATTACAACACTTTTTGAGACGTCCCATAGCCCTTTAG
tenrec       ----------------------------------------------------------------------------------------------------
monodelphis  --------------------------------------------AGTACCAAGGACTAAGAACC------------------------------------
platypus     ----------------------------------------------------------------------------------------------------
chicken      ----------------------------------------------------------------------------------------------------
xenopus      ----------------------------------------------------------------------------------------------------
tetraodon    ----------------------------------------------------------------------------------------------------
fugu         ----------------------------------------------------------------------------------------------------
zebrafish    ----------------------------------------------------------------------------------------------------
```

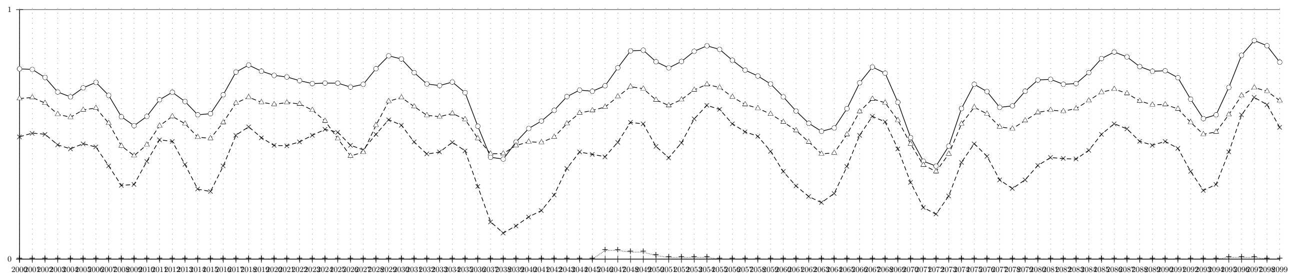

```
human        TTCCATTTTACTTTTCTTTTCTCATATTAAAATTAACTAAATACTATTTGAAAGAGAATGTACGGTATAACAAACTAATACAATTTCTCTCTTGCTTAGA
chimp        TTCCATTTTACTTTTCTTTTCTCATATTAAAATTAATTAAATGCTATTTGAAAGAGAATGTACGGTATAACAAACTAATACAATTTCTCTCTTGCTTAGA
colobus      ----------------------------------------------------------------------------------------------------
baboon       TTCCATTTTGCTTTTCTTTTCTCATATTAAAATTAACTAAATACTATTTGAAAGAGAATGTATGGTATAACAAACTAATAAAATTTCTCGC----TTAGA
macaque      -----------------------------------------------------------------------------------------------TTAGA
dusky titi   ----------------------------------------------------------------------------------------------------
owl monkey   ----------------------------------------------------------------------------------------------------
marmoset     CTCCATTGTACTT---TTTTCTCACATTGAAATTAATTAAATACTATTTGAAAGAGAATGTACAGCGTAACAGACTAGTAAAATTTCTCTCTTGCTTAGA
mouse lemur  ----------------------------------------------------------------------------------------------------
galago       TTCTGTTTTACTTTCTGTTTCTCACAGTAAAATTAACAGAACACTGTTTGCAAGAGAATGCATACTATAATAAACTAAGAGAATTTCTTCC----TTAGA
rat          CCCCACTTCCTGTTTCCTTACATGAAGTAAAGGCAACATGGAGCTG------GGAGGATA----------------------------------------
mouse        CCCCATTTAGCGTTTCCTTGCATGAAGTAACAGTCACCTGGTGCTATTTGGTGGGGGTG----------------------------------------
rabbit       ---------------------------CAAAATCAA-----TACTGACTGAAAGAGAATGTGTGTTGTAACAGCTTTCTAAAG---CTCTATTGG-----
cow          TTCTATCTTCCCTTTCTTTTTTCACATTAAAGTTAA-----TACTATTTGAGAGAGCACATATGGTATTA--GTTTTCCAAAGTTTCACTCTTCATTAGA
dog          ---TATTTCACTTTCCTTTTCTCACATTAAAATAAA-----AAATATCTGAAAAAGAATGTATGGTATTATGATTTTGCTAAATGTCTCTCTTTGTTAGA
rfbat        TTCTGTTTTAGTTTCCATTTCTCACATAAAAATTAA-----TGCTGTTTGAAAGAGAATGTATGTTATTATAGCTTTCCAAAGTTT--CTCTTTGTTAGA
hedgehog     ----------------------------------------------------------------------------------------------------
shrew        ----------------------------------------------------------------------------------------------------
armadillo    TTCCATTTTACTTTACTTTCCTTACATGAAAATTAATTATATATTGTTTGAAAGAGAATGTGTAGTATTCAGTTTTTCTAAAACTCCTCTCTCTGATATA
elephant     TTCTGTTTTACTCCCATTTTCTTATAT----------------------------GATTGAGTGTTATTCCACTTTTCTAAAATTTCTCTCTGTA-----
tenrec       ----------------------------------------------------------------------------------------------------
monodelphis  ----------------------------------------------------------------------------------------------------
platypus     ----------------------------------------------------------------------------------------------------
chicken      ----------------------------------------------------------------------------------------------------
xenopus      ----------------------------------------------------------------------------------------------------
tetraodon    ----------------------------------------------------------------------------------------------------
fugu         ----------------------------------------------------------------------------------------------------
zebrafish    ----------------------------------------------------------------------------------------------------
```

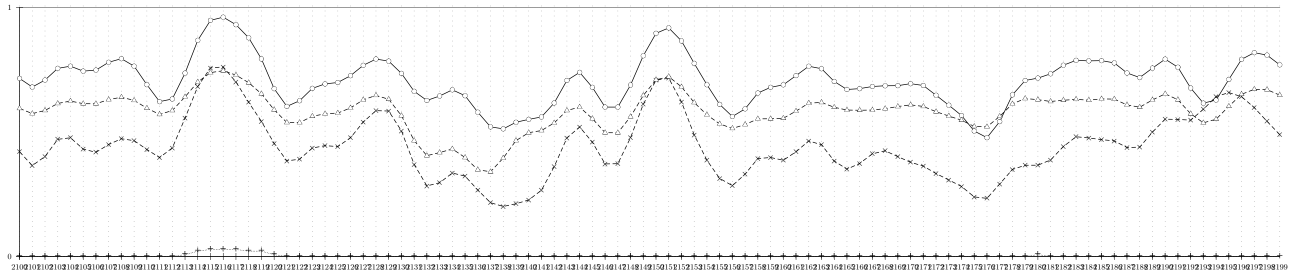

human	CTTGCTGAGATGTGTTTAAAGAGATGGTCATGATTTTTTAAGTTGGCAGTAGAATTTTGGGTGATTTAGTTTGTTTACTTTTTGCTTTCTCTTCCTACTT
chimp	CTTGCTGAGATGTGTTTAAAGAGATGGTCATGATTTTTTAAGTTGGCAGTAGAATTTTGGGTGATTTAGTTTGTTTACTTTTTGCTTTCTCTTCCTACTT
colobus	--
baboon	CTTGCTGAGATATGTTTAAAGAGAAGGTCATCATTTTTTAAGTTGGCAGTAGAATTCTGGTTGATTTAGTTTGTTGACTTTTTGCTTTCTCTTCCTACTT
macaque	CTTGCTGAGATATGTTTAAAGAGAAGGTCATCATTTTTTAAGTTGGCAGTAGAATTTTGGTTGATTTAGTTTGTTGACTTTTTGCTTTCTCTTCCTACTT
dusky titi	--
owl monkey	--
marmoset	CTTGCTGAGATGTGTTTAAAGAGATGATCATCATTTTACAGGCTGACATTAGAATGTTGGGTAATTTAGTTTATTTACTTTCTGTTTTCTCTTCCTACTT
mouse lemur	--
galago	CTTGCTCAGA----------GGTATGGTCATATTTTT---GGCTGGTGGTAGATTTTTGGGTGACTTTGCTTGCTTTTTCTTTGCTTTTTCTTCGTATTT
rat	--
mouse	--
rabbit	----------------TATAGAG---------TTTGCTCATGGTAGGAGC-GACTTCTGTGTGACTTGGTGTGCTC-----TGGCTTGCTCTGTGTACCT
cow	CCAGTTGAGATGTATTTAAAATGACAGTTGTCACTTTATTGACTGGCCGTAGAATT-----GGA-----------------CTGATTTATTTGC-----T
dog	ATTGCTGAGTTACATTTAAA-TGATGGTCATCATTTTATTGACTGGTGGTAGAATTCAGAATGA-----------------TTGCTTTCTTTTCAAACTT
rfbat	CTTGCTAAGATGTATTTAAAACGATGGTCATCATTTTGTTGGATGGTGGTAGAATTTGGGGTGATAT-GCTTGT-------TTGCTTTCTTTTCATACTT
hedgehog	--
shrew	--
armadillo	TTAGCCGAAATATGTTTAAAATAATGTTCATCAA-GTATTGACTGGTGGTAGAAGTTGGGGT------GCTTATTTGCTTTTTGCATTTTCTTTGTACTT
elephant	--
tenrec	--
monodelphis	--
platypus	--
chicken	--
xenopus	--
tetraodon	--
fugu	--
zebrafish	--

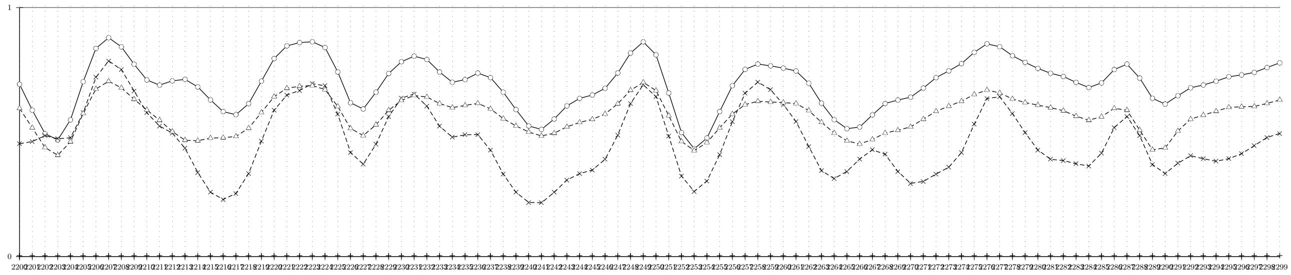

human	TTCAATCCCCAAGGAAATGGCCACCAGAGCTTGACACTGTTATCATTATTAATGCAGTAGTGACAGTGGGCAGAAGCAGCAAAACAATTACAAATTTCCT
chimp	TTCAATCCCCAAGGAAATGGCCACCAGAGCTTGACACTGT-ATCATTATTAATGCAGTGGTGACAGTGGGCAGAAGCAGCAAAACAATTACAAATTTCCT
colobus	--
baboon	TTCAATCCCCAGGGAAATGGCCACCAGCGCTTGACACT---ATCATTATTAATGCAGTGGTGACAGTGGGCAGAAGCAGCAAAGCAATTACAATTTTCCT
macaque	TTCAATCCCCAGGGAAATAGCCACCAGCGCTTGACACT---ATCATTATTAATGCAGTGGTGACAGTGGGCAGAAGCAGCAAAGCAATTACAATTTTCCT
dusky titi	--
owl monkey	--
marmoset	TTCAATCCCCAGGGAAACAGCCACCAGGGCTTGACACT---ATC---ATTAACACAGTG------GTGGGCAGAAGCAGCAAAGCAATTACGAGTTTCCT
mouse lemur	--
galago	TTTGATCCCCAGGGGAACAGCCACCAAAGCTTGACACTGG-ATCATAACTAATACGCTGGT-ACAGAGAGTAGA---ATCAAAGCAATTCTAATTCTAC-
rat	--
mouse	--
rabbit	TCCGACCCTGGGGGAGACGGCCACCAGAAGGCGGTGCTGT-GTCCCT-CTAGCACA-CGGCGGCCCCAGACTGAAGCATCTGAGATGTTCCAGTGTTACT
cow	TTATCTCCCCAAGGACTCAGTCTCCATAGCTTGATGCTCT-GGCATTACTAACATACTGGTGACACCGGTAAAAAGCAACAAACCCATTACAATTTCTCT
dog	CTGGATCCCCAAGGAATCAGCCACCACAGCTTGATGCCAT-ACCATTGCTAATGTACTGATGATACAGGGCAGAAACATCAGAGCAATTATAATTTCACC
rfbat	CTAGGTCCCCAGGTAATCAGCCACCACAGCTTGATTCTGT-ACCCTCACTAACAGACTGGTGATGCCAGGCA-CGGCATCAAAGCAATTACAATTTCACA
hedgehog	--
shrew	--
armadillo	TTCTGTCCCCAAGTAAGTAACCATC--CGCTTGCTGC----TTTATTACTAATGTGCTGGC-------------------------ATAATAATTTTACT
elephant	--
tenrec	--
monodelphis	--
platypus	--
chicken	--
xenopus	--
tetraodon	--
fugu	--
zebrafish	--

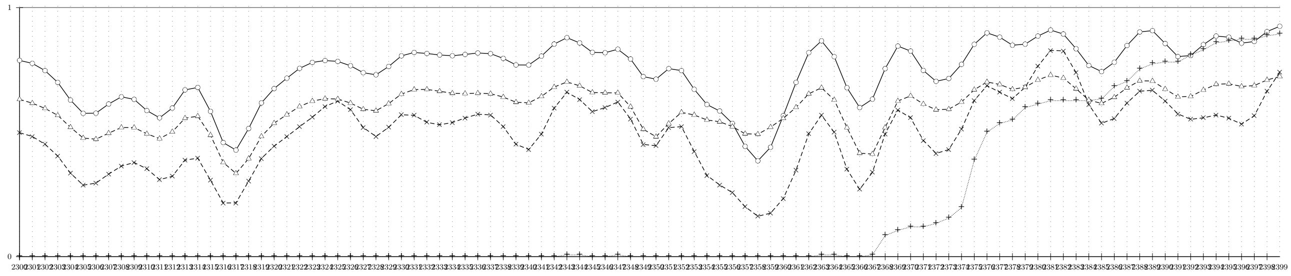

human	TGCTCCACACATGCTTACTGGGCACACAGAATTTCTACAAAAGCAAATTCATTATTTGTGAAAAAGGAAAAATCAGAAAATACCAGACAAACCAAAAGAA
chimp	TGCTCCACACATGCTTACTGAGCACACAGAATTTCTACAAAAGCAAATTCATTATTTGTGAAAAAGGAAAAATCAGAAAATACCAGACAAACCAAAAGAA
colobus	--
baboon	TGCTCCACACAGGCTTACTGGGCACACAGAATTTCTACAAAAGCAAATTCATTATTTGTGAAAAAGGAAAACTCAGAAAATACCAGACAAACCAAAAGAA
macaque	TGCTCCACACAGGCTTACTGGGCACACAGAATTTCTACAAAAGCAAATTCATTATTTGTGAAAAAGGAAAAATCAGAAAATACCAGACAAACCAAAAGAA
dusky titi	--
owl monkey	--
marmoset	TGCTCCACACACGCTTACTGAGCACACAGAATCTCTACAAAAGCACATTCATTATTTGCAAAAAAGGCAAAATCAGAAAATACCAGACAAACCAAAGGAA
mouse lemur	--
galago	TGCTGCACTCATGCTTGT--GGCACACACAATTTCTACACAAGCAAATTGCTTAC--------AAAGAAAAATCAGAATACACCAAACAAACCCAAAGAA
rat	--
mouse	--
rabbit	TGGTCTGCACATCTTTAGAGGCTTCACATAATTTCTACAAAAACAAATTTCTTATTCTTAGAAAAAGTAAA-TCAGAAAATACCA----AAGCAAAAGAA
cow	TGCTCTATACATTCTTCACGAGCACATATAATTTCTTCAAAGGCAAATTCATTATTCAAAAGAAAAAAAAAATCAGAAAATACCGGATA-----------
dog	TGCTCCAGAGGTGCTCCATGGGCACACAT----TCTACAAAAGCAAATTCATTCCCC---AAAAATGAAAAATTGGAAAATACC----------------
rfbat	TGCTTAACAC-----------GCACACCTAATTTGTATAAAAGCAAATTCATTGTTC----TAAAAACAAAGCCTGAAAATACTAGACAAACCAAAAGAA
hedgehog	--
shrew	--
armadillo	TTATCCGCATAGGCTAACTGGGCATGCATTACTTTT--AGCAGCAGG------------------------------ATATACCACACAAAACAAAAAAA
elephant	--
tenrec	--
monodelphis	--
platypus	--
chicken	--
xenopus	--
tetraodon	--
fugu	--
zebrafish	--

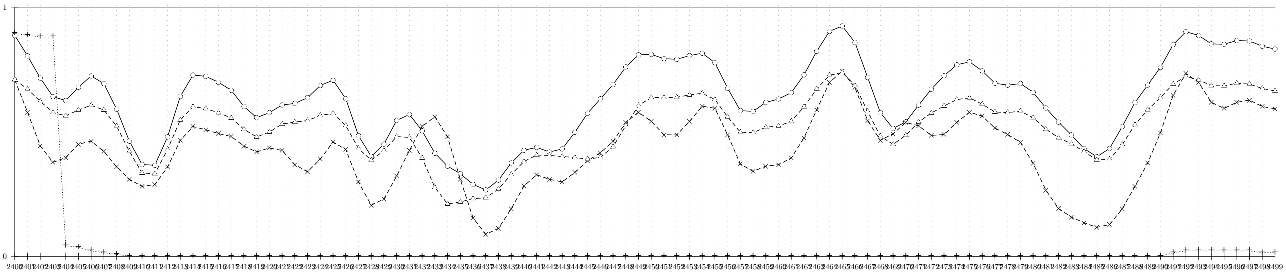

human	TAAGTCAAAGCTTCAGTACCGAGAAGTTAACACTGTTAACATCATCTGAGTTAACTATTACCCTTCTGATGTATTCATGCTACTCTATTTGAGTAAAATG
chimp	TAAGTCAAAGCTTCAGTACCGAGAAGTTAACACTGTTAACATCATCTGAGTTAACTATTACCCTTCTGATGTATTCATGCTACTCTATTTGAGTAAAATG
colobus	--
baboon	TAAGTCAAGGCCTCAGTACCGAGAAGTTAACACTGTTAACATTGTCTGAGTTAACTATTAACTTTCTGATGTATTCATGCTGTTCTATTTGAGTAAAATG
macaque	TAAGTCAAGGCCTCAGTACCGAGAAGTTAACACTGTTAACATTGTCTGAGTTAACTATTAACTTTCTGATGTATTCATGCTGTTCTATTTGAGTAAAATG
dusky titi	--
owl monkey	--
marmoset	TAAGTCAAAGCCTCACTACCGAAAAGTTAGCACCGTCCACATCACCTGAGTTAAGTACTAACCTTCTGAGGTATTCATGTTAT--TGTTTGAGTAAAATG
mouse lemur	--
galago	TAGAACTAAAAATCATTA---ATAACTCTACTCCACAGAGGTGACCTCAGTTAACTGGTAATCTTCTTACATGTCCATGCTGTCCACTGTCAGTAAAAT-
rat	--
mouse	--
rabbit	TAAATCAAAGCCCCACGATGCAGAGGTGGGCCCTGCTGCCCT---GTCAGAGAACTGCCAGGCCTCTGACGCAGCCAGGCCACTCACCTCCAGTGAGAAG
cow	-GAACCATAACACCACCACCCAAAAGTTCATAATGTCTGGTTCCC---AGTTGACTGTTAACCTTCTGGTATGTCCACACTGTTA-CATTTAGTAAAATG
dog	--------ACTGTTACTAAATAACAGTGT-TAACCTCTAGATTAC------TAGCTGTGAGCCTTCTGATATATCCATACTGTTCTCTTTTAGTAAAATG
rfbat	TGAAACATAACTCCACTACCCAGAGGT---TAACTTCCAGGTTACC--AGTTAACTGCTAACCTTCTGCTGTATCTATACTGTTAATTTTTAGTAAAATA
hedgehog	--
shrew	--
armadillo	TAAACCATAATCCCACTACTCAGAAGTTAACACTATTAGCAT---GTGGGTTAACTGTTAACCTTCTGGTGTATCCAAACTGTGAATTTTTAGTCAAATG
elephant	--
tenrec	--
monodelphis	--
platypus	--
chicken	--
xenopus	--
tetraodon	--
fugu	--
zebrafish	--

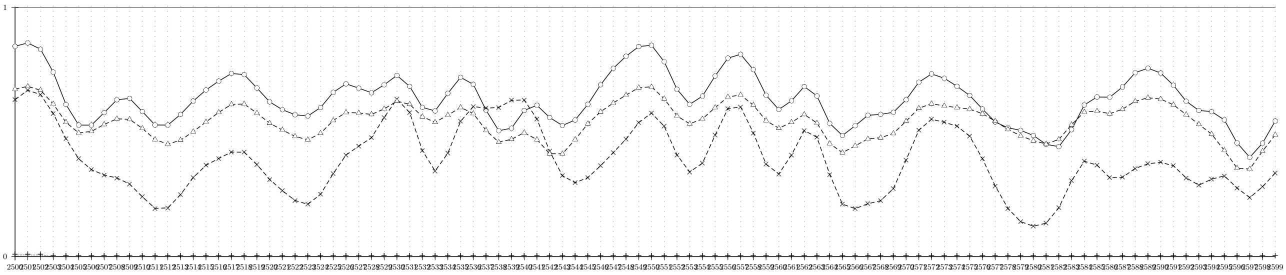

human	AGATCATACTTTATAGTTTTATAGTTTACACCCAATCATATATCACTAAAAAACTTTGAAATCATTAAGTGCAGCATGTTTTTTTTCTTTTTGAGACAGG
chimp	AGATCATACTTTATAGTTTTATAGTTTACACCCAATCATATATCACTAAAAAACTTTGAAATCATTAAGTGCAGCATGTTTTTTTTCTTTTTGAGACAGG
colobus	--
baboon	AGATCGTACTTTACAGTTTTATAGTTTACACCTGATCATGTATCACTAAAAAACTTTGAAACCATTAAGTACAGCAT-TTTTTTTTCTTTTTGAGACAGG
macaque	AGATCGTACTTTACAGTTTTATAGTTTACACCTGATCATGTATCACTAAAAAACTTTGAAACCATTAAGTACAGCAT-TTTTTTTTCTTTTTGAGACAGG
dusky titi	--
owl monkey	--
marmoset	AGATCGTACTTTACAGTTTTATACTTTACA-CTGATCATCTAACACTAAAAAACTCTGAAATCATTAAGTACAGCAT-TTTTTTTCCTTTTTGAGACCAG
mouse lemur	--
galago	---------TTTACAGTTTTATATTTTACACCTATTCACGTAACATTAAAAAATTCTGACATCACTGAATACAACAT-CTTTATTCCATCCTATGACAAG
rat	--
mouse	--
rabbit	GGATCACCCTTCTGCAGCTGGCAGGCTACG-CCAATCACGCATCACAAGGAAACCCGGATGCCAGCAGCGACATCGC-CCCA------------------
cow	GGATCATATTTACCAGTTTTAT-AGTTACACCTAATCCTATACCCTTAAAAAA-TCTGGTATCAGTAAGTATA-----TTTCTATCCTTTTTGATACCTG
dog	GGGTA---CTTGACAGCTTTAC-GTTTATACCTAATTGCATACTGTTTAAAAG-GCTGATGTCAGGAAGCATA-----TGTCCATCATTTTTAATAGCTG
rfbat	GGATCATACAGAACTGTTTTGTTGTTTATACCCAATCATATATTGTTAAAAAG-TCTGATGTCAACCAGTATA-----TTCCCTTCTTTTTTAACAGGTG
hedgehog	--
shrew	--
armadillo	GGATAGTG---TACAGTTTCATAGTTGTTCCCAGTCATATATCATTAAAAAC-TCTGATGGCAATAAATGTA-------TT------------------
elephant	--
tenrec	--
monodelphis	--
platypus	--
chicken	--
xenopus	--
tetraodon	--
fugu	--
zebrafish	--

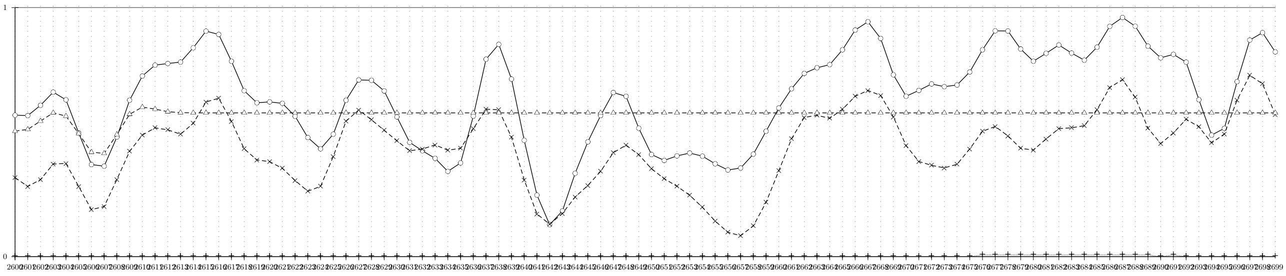

human	GCCTCCCTCTGTCCTCCAGGCTAGAGTGCAGTGGCGCAATCTCCACTTACTGCAGCCTTGACATCCCAGGCTCAAGCAATTCTCCCACCTCAGCTTCCCA
chimp	GCCTCCCCCTGTCCTCCAGGCTAGAGTGCAGTGGCGCAATCTCCACTTACTGCAGCCTTGACATCCCAGGCTCAAGCAATTCTCCCACCTCAGCTTCCCA
colobus	--
baboon	GCCTCCCTCTGTCCTCCAAGCTAGAGTGCAGTGGTGCAATCTTGACTTACTGCAGCCTTGACATTCCAGGCTCAAGCAATTCTCCCACCTCAGCCTCCCA
macaque	GCCTCCCTCTGTCCTCCAAGCTAGAGTGCAGTGGTGCAATCTCGACTTACTGCAGCCTTGACATTCCAGGCTCAAGCAATTCTCCCACCTCAGCCTCCCA
dusky titi	--
owl monkey	--
marmoset	GCCTCC--CTGTCCTCCAGGCTAGAATGTAGCGGCACAATCCTGACTTACCACAGCCTTGACATTCCAGGTTCAAGCAATTCTCCCACCTCAGCCGCCCA
mouse lemur	--
galago	TTTGCTCAATCTCTTCCAGACAATTATG-AGGAGTCCAATTTTCAGCTATCGTGAAAAAAAAATTCC-----CCAACAATACTCACACCTTAGCTTCCCA
rat	--
mouse	--
rabbit	--
cow	CATTCCTCCT--
dog	CATTCCATCT--
rfbat	CATTCTACCT--
hedgehog	--
shrew	--
armadillo	--
elephant	--
tenrec	--
monodelphis	--
platypus	--
chicken	--
xenopus	--
tetraodon	--
fugu	--
zebrafish	--

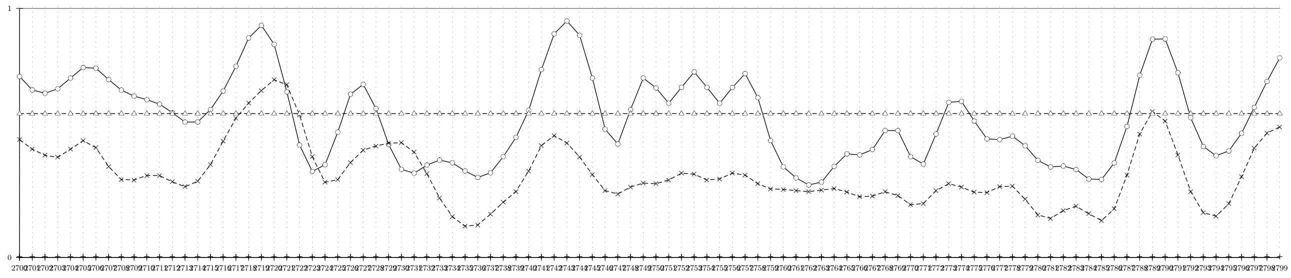

human	AGTAGCTGAGACAACAGGTACACGCCAACACACCCAGCTAACTTTTGTTTTGTTTGTTTGTTTGTTTGTTTGTTTTGGTAGAGACAGAATCTCACTATGT
chimp	AGTAGCTGAGACAACAGGTACACGCCAACACACCCAGCTAACTTTTGTTTTGTTTGTTTGTTTGTTTGTTTGTTTTGGTAGAGACAGAATCTCACTATGT
colobus	--
baboon	AGTAGCTGAGACAATAGGTACACGCCAACACGCCCAGTTAACTTTTG---------------TATTTTTTTCTTCTGGTAGAGATAGAATCTCACTATGT
macaque	AGTAGCTGAGACAATAGGTACACGCCAACACGCCCAGTTAACTTTTG---------------TATTTTTTTCTTCTGGTAGAGACAGAATCTCACTATGT
dusky titi	--
owl monkey	--
marmoset	AGTAGCTAAGACAAC----ACACACCAACATGCCCAGCTAACTTTTT---------------------TTTTTTTTTGTAGAAACAGAATCTCACTATGT
mouse lemur	--
galago	TGGAACTGATAAATCAAG--CATCTTAAAAGGTTTTTTTTTCTTTTTCTTTCTTTCTTTCTGTCTCTCTTATTTTTTTTTTTAATTTTATCTTTTCTTAT
rat	--
mouse	--
rabbit	--
cow	--
dog	--
rfbat	--
hedgehog	--
shrew	--
armadillo	--
elephant	--
tenrec	--
monodelphis	--
platypus	--
chicken	--
xenopus	--
tetraodon	--
fugu	--
zebrafish	--

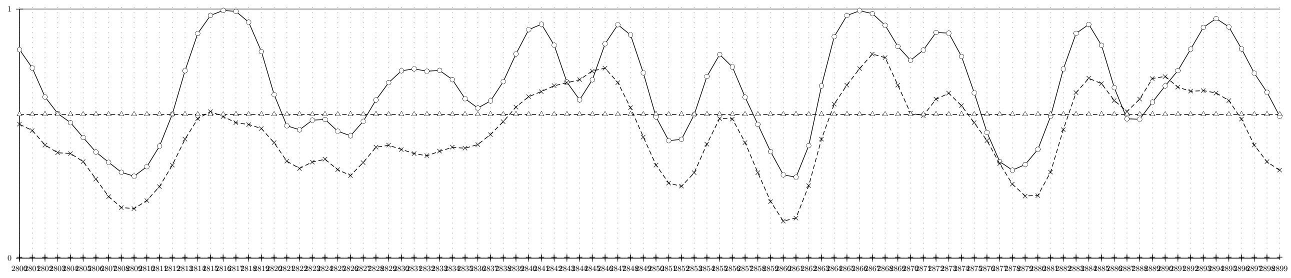

human	TGTCCAGGATGGTCTCAAGAACTCCCGGGCTCAAGCAATCCTCCAGCCTCCAACTCCCAAAATGCTGGGATTACAGGTGTGAGCCACGACGGCCAGCCTC
chimp	TGTCCAGGATGGTCTCAAGAACTCCCGGGCTCAAGCAATCCTCCAGCCTCCAACTCCCAAAATGCTGGGATTACAGGTGTGAGCCACGACGGCCAGCCTC
colobus	--
baboon	TGCCCAGGACGGTCTCAAGAATTCCTGGGTTCAAGCAATCCTCCCGCCTCCACCTCCCAAAATGCTGGGATTACAAGCGTGAGCCACCACGGCCAGCCTC
macaque	TGCCCAGGACGGTCTCAAGAATTCCTGGGTTCAAGCAATCCTCCCGCCTCCACCTCCCAAAATGCTGGGATTACAGGCGTGAGCCACCACGGCCAGCCTC
dusky titi	--
owl monkey	--
marmoset	TGCCCAGGATGGTCTCAAGAATTCCTTCGCTCAAGCAACCCTCCCGCCTCCACCTCCTAGAGTGCTGGGATTACAGGTGTGAGCCACCACGGCCAGCCTC
mouse lemur	--
galago	TGCTCCCAGCAACCTCA--AACTACTGGGCTCCAGTGATCCTCCTGCCTCAGCTTCCTAAGTAGCTGGGAATACAGGAACCTGCCACTATGACCAGCT--
rat	--
mouse	--
rabbit	--
cow	--
dog	--
rfbat	--
hedgehog	--
shrew	--
armadillo	--
elephant	--
tenrec	--
monodelphis	--
platypus	--
chicken	--
xenopus	--
tetraodon	--
fugu	--
zebrafish	--

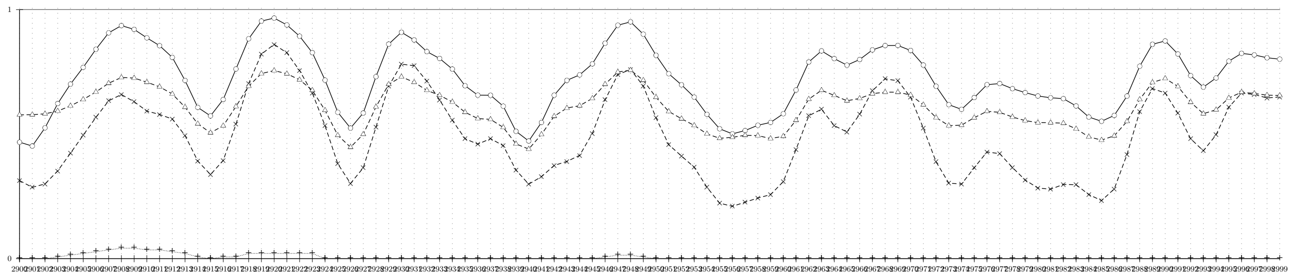

human	CAGAGTATTTTTAATCGCTGCATTCCATCTTATGACCAGTCCCCAATTTGCTCAGCCAATCCCTTACTGGTGGATAGTTAGAAGACCCATTTCCAGCTAA
chimp	CAGAGTATTTTTAATCGCTGCATTCCATCTTATGACCAGTCCCCAATTTGCTCAGCCAGTCCCTTACTGGTGGATAGTTACAAGACCCATTTCCAGCTAA
colobus	--
baboon	TAGAGTATTTTTAATCGCTGCATTCCGTCTTATGACCAGTCCCCTATTTGCTCAGCCAATCTCTTAATGGTGGATAGTTAGAAGTCCCATTTCCAGCTAA
macaque	TAGAGTATTTTTAATCGCTGCATTCCGTCTTATGACCAGTCCCCTATTTGCTCAGCCAATCTCTTACTGGTGGATAGTTAGAAGTCCCATTTCCAGCTAA
dusky titi	--
owl monkey	--
marmoset	TATAATATTTTTAATTACTGCATTCCATCTTATGACCAGTTTTTAATTTGCTCAGCTAATCCCTTACTGGTGGACAGTTAGAAGTCCCATTTCTAGCTAA
mouse lemur	--
galago	---AGTTTTTTT----------TTCTATTTT---------------TTTGTAGAGATGGGGTCTGGCTCTTG------------------CTCAGGCTAG
rat	--
mouse	--
rabbit	----------------GCTGCACTTCCTCTTATGGCTGGCGCTCAGTTTGCTCAACTAATCCCTTACTGGTATACAGTTAGGAGCCCAATTTTCAGCGAA
cow	-------TTTTTGATACCTGCATTCCTCCTTATGACCAGTGCTCAATTTGCTCAGTC----CCTTGCTGGTGGGTAGTAAGGAGTCCGATTTTCAGCTAA
dog	-------TTTTTAATAGCTGCATTCCATCTTACGATTAGCCCTCAGTTTGCACAATCAATGCCTTGCTGGTGGACAGTTAGGAGTTCAATTTTCAGCTAA
rfbat	-------TTTTTAACAGGTGCATTCTACCTTATGACCAGACCTCAATTTGCTCGGTCGATACCTTGCTGGTGGACAGTTAG----CTAATTTTCAGCTAA
hedgehog	--
shrew	--
armadillo	-----TATTTCTAAGAGCTGCATTCCCTCTTACGACTAGCTCTCAATTTGCTCAACCAATCCCTTGCTGGTGCATTAGGAAATGCTCAATTTTCGGTTAA
elephant	---TGGTGGAATTCTTATTTTTAGCTCA
tenrec	--
monodelphis	--
platypus	--
chicken	--
xenopus	--
tetraodon	--
fugu	--
zebrafish	--

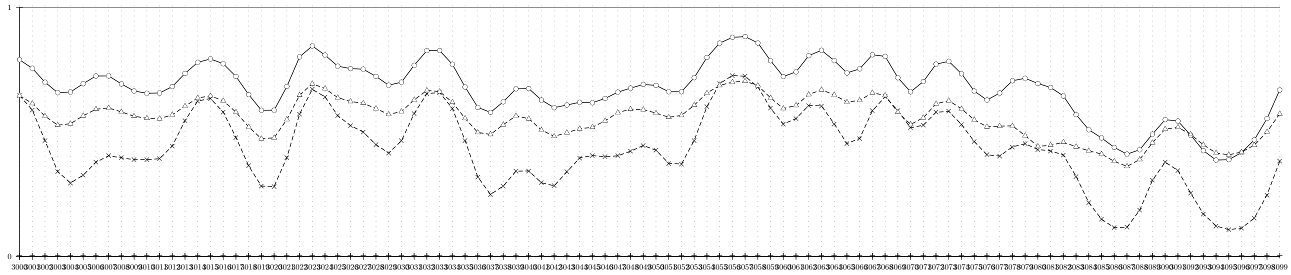

human	TATAAATATTATTCCCTAACAAAACCTCTACTCACATCTTTAATTATTTCACTGGGATAAATGACTAGACATGGAATTGCAGGATCAAGTATCTTAAAAG
chimp	TATAAATATTATTCCCTAACAAAACCTCTACTCACATCTTTAATTATTTCACTGGGATAAATGACTAGACATGGAATTGCAGGATCAAGTATCTTAAAAG
colobus	--
baboon	TATAAATATTATTCCCTAACAAAACTTCTACTCACATCTGTAATTATTTCACTGGGATAAATGACTAGACATGGAATTGCAGGATCAAGTATCTTAAAAG
macaque	TATAAATATTATTCCCTAACAAAACTTCTACTCACATCTGTAATTATTTCACTGGGATAAATGACTAGACATGGAATTGCAGGATCAAGTATCTTAAAAG
dusky titi	--
owl monkey	--
marmoset	TATAAAGAATATTCCCTAATAAAACCTCTACTCACATCCTTAATTATTTCACCGGGATAATTGACTAGACATGGAACTGCTGGATCAAGGATCTTAACAG
mouse lemur	--
galago	TCTTGA----ACTCCTGACCTTAACTGATCCTCCCACTTTGGCCTCCCAGAGTGG----------------TAGAATTACAGGCCTGAGCATGCCCAGAT
rat	--
mouse	--
rabbit	CATGAAAAATCTTCCCTAACAAAACCTCTTCGCACACTCTTAATTCTTTCCTTAGTACAAG-GACCAGACGTGGGACTGCTG-CTCAAACAGCCTAGGGG
cow	TAGAAAAAGTATCCCCTAACAAAACCTCTACTCAACACCTTAATTATTCCCTTAGGATAAACGGCTGGACGTGGAATGGCTGG-----------------
dog	TATGAAAAATATCCCCTAAGAAAACCTCTACTCACATCCTTAATTCTTCCCTTAGGATAAATGACTAGACATGGAAAGGCCAGAATGTG-----------
rfbat	TATGGAAAACAGCCCCTAACCAAACCTCTAGTCACATCCTTCATTATTTCCTCAGGATAAATGACCAGACATGGGATGGCTGGCTCAGT----TCAAAAG
hedgehog	--
shrew	--
armadillo	TATGAAAAATATCCTCTAACCCAATCTCCATTCACATCCTTAGGTGTTTCCTTGGGATAAGTGACTGGACCGGGCACTGCTGGGTCCCACACCTGGGAAA
elephant	TATGAGAAACATGGCCTTCCCAAATCTCTATTCACATTCTTACTTATTTCCTTAG-------------AGGTGGAACTCCTAGGTCAAAC----------
tenrec	--
monodelphis	--
platypus	--
chicken	--
xenopus	--
tetraodon	--
fugu	--
zebrafish	--

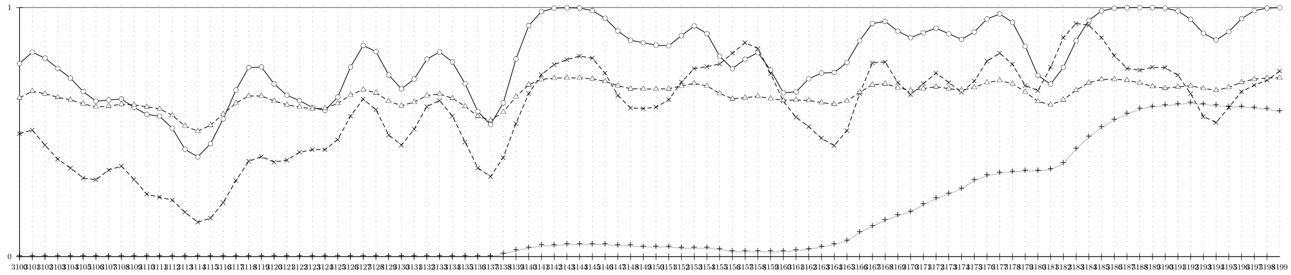

human	TTTTTTTATCTTTTTTCTTTATCTATTGTGCAACCCACTTCAGTGCCAGCAGCCTACGGGAACAGAGGCCAGGCAATGGTTCAGGGCTACAGTTTGCTCT
chimp	TTTTTTTATCTTTTTTCTTTATCTATTGTGCAACCCACTTCAGTGCCAGCAGCCTACGGGCACAGAGGCCAGGCAATGGTTCGGGGCTACAGTTTGCTCT
colobus	--
baboon	TTGTTTTATCTTTTTTCTTTATCCACTGTGCAACCCACTTCAGTGCCAGCAGCCTATGGGCACAGAGGCCAGGCAATGGTTCGGGGCTACAGTTTGCTCT
macaque	TTGTTTTATCTTTTTTCTTTATCCATTGTGCAACCCACTTCAGTGCCAGCAGCCTACGGGCACAGAGGCCAGGCAATGGTTCGGGGCTACAGTTTGCTCT
dusky titi	--
owl monkey	--
marmoset	TT-TTTTATCTTTCTTCCTTATCCA-TGTGCAACCCCAGTCAGTGCCAGCAGCCTACGGGCACACAGGCCAGGCAATGGTTCGGGGCTACAGTTTGCTCT
mouse lemur	--
galago	TTTCTTTTTTCCTCATCTTTTTCCACTGTACGACCCACTTCAGTGCCAGCAACCTATGGGTAGAGAGGCCTGGCCATGGTTCGGGGC--CAGTTTGCTCT
rat	--
mouse	--
rabbit	TTTTTCCATTTTTTCCTTTTTCCTACTGTGGAACACATTTCAGTGCCAACAACCTACGG--ACAGTGGCCAGGCAATGGTGTGGGGCTAC-GTTGGCTCT
cow	--
dog	--
rfbat	TTT---
hedgehog	--
shrew	--
armadillo	TTTT--
elephant	--
tenrec	--
monodelphis	--
platypus	--
chicken	--
xenopus	--
tetraodon	--
fugu	--
zebrafish	--

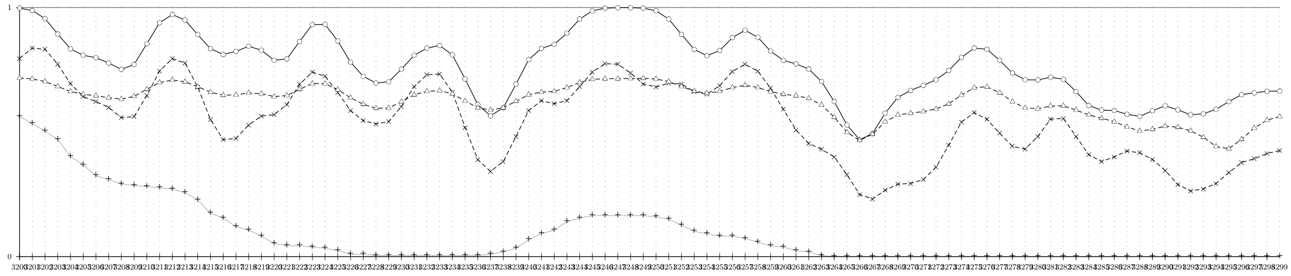

human	GGCATGGGGTACTATGAGAGGTCCAGGGGGACGGGCCCATCATAAATCCAAACCCATGCATAAGCACCTTAAAAGTTTTGATGTGTACAGCCAAACTTCC
chimp	GGCATGGGGTACTATGAGAGGTCCAGGGGGACGGGCCCATCATAAATCCAAACCCATGCATAAGCACCTTAAAAGTTTTGATGTGTACGGCCAAACTTCC
colobus	--
baboon	GGCATGGGGTACTATGAGAGGCCCAGGGGGACGGGCCCATCATAAATCCAAACCCATGCATAAGCATCTTAAAAGTTTTGATGCGTACAGCCAAAC-TCC
macaque	GGCATGGGGTACTATGAGAGGCCCAGGGGGACGGGCCCATCATAAATCCAAACCCATGCATAAGCATCTTAAAAGTTTTAATGCGTACAGCCAAAC-TCC
dusky titi	--
owl monkey	--
marmoset	GGCATGGGGTACTATGAGAGGCCCAGGGGGACAGGCCCATCATAAATCCAAACCCATGCATGAGCATCTTAAACGTTTTGATGTGTACGGCCAAACTTCC
mouse lemur	--
galago	GGCACGGGGCACTATGACAGGCCCAGAAGTACAGGCCTATCAGAAAT-CAAACCCGTGCGTAAGCATCTTGAA-GCTTTGATGTACACGGCCAAACTGCC
rat	--
mouse	--
rabbit	GGCATGAGATACTATAAGAGACCCAGGGGAACAGGTTTGCCATAAATCCAAACTCATGCATAAACAGCCTAGGGGTTTTGACG-----------------
cow	---CAGCTCAGCTGCC
dog	--TGTGTTTAAAGTTCTGATGCATACGGCCTAAATGCC
rfbat	--CAGTTCAAAAGTTTCAACGTATCCACATCCGATGGC
hedgehog	--
shrew	--
armadillo	---GCTG-----------------
elephant	--CGTTTTAAAAGTTTTGATG--TTCAGCCAAACTATC
tenrec	--
monodelphis	--
platypus	--
chicken	--
xenopus	--
tetraodon	--
fugu	--
zebrafish	--

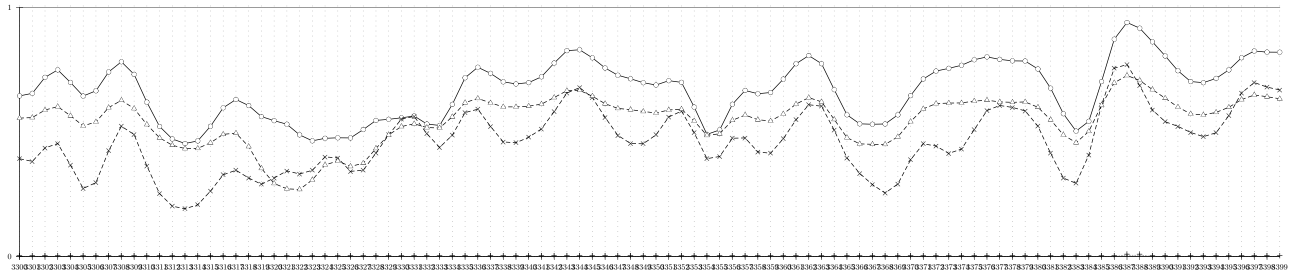

```
human        CAGCAGAAACATTAAACTGTAGTATGTTCCAGCCCCCTTCAACAGTGTAAATGGTACCAACTGTCACTGAATAAAAGGCCAGACAGCTGAAATGGCCCAC
chimp        CAGCAGAAACATTAAACTGTAGTACGTTCCAGCCCCCTTCAACAGTGTAAATGGTACCAACTGTCATTGAATAAAAGGCCAGACAGCTGAAATGGCCCAC
colobus      ----------------------------------------------------------------------------------------------------
baboon       CAGCAGAAACATTAAACTGTAGTATGTTCCAGTCCCCTTCAACAGTGAAAATGGTACCAACTGTCCTGGAATAAAAGGCCAGATGGCTGAAATGACCCAG
macaque      CAGCAGAAACATTAAACTGTAGCACGTTCCAGTCCCCTTCAACAGTGAAAATGGTACCAACTGTCCTGGAATAAAAGGCCAGATGGCTGAAATGACCCAG
dusky titi   ----------------------------------------------------------------------------------------------------
owl monkey   ----------------------------------------------------------------------------------------------------
marmoset     CAGCAGCAACATTCAACTGTAGCACATTCCAGTCCCCTTCAACAGTGTAAATGGTACCACCTGTCCTTGAATAAAAGGCCAGACAGCTGAAATGACCCAG
mouse lemur  ----------------------------------------------------------------------------------------------------
galago       CAGCACAAACATCATGCTACAGTACACTCT-GCGTCCTTCAACAGTGTAAATGGTACCCGCTGTCTTTATATAAAAGGCCAGATGGCTGAGATGACCCAG
rat          ----------------------------------------------------------------------------------------------------
mouse        ----------------------------------------------------------------------------------------------------
rabbit       ---CAGAAAGCCCGCACTGCCGGACGTGCTG--CCCCTTCGGCAGTGTAAACGGAGCTCACTGTCCTTCAGCAGCAGGCCAGGCGGCTGAGAGGGCCCGG
cow          CTGCTGAAACTTTA-CCAGAAGTACATCCTTGC-TCCTTTAGCAGTGTGGACGACACCAAATGTCCTTTAATAAAA-GCCAGACA-CTGAGAAGACGCAG
dog          TTGC-AAAACACCA-ATTGAAGTGCAGCCCTG--TCTGTTAGCAGTGTAGGTGACCCCAAAAGTCTTTTAATGAAA-GTCAGACAGCTGAGATGAGCCAG
rfbat        CGGC-AGGACACTA-ACTGAGGTGCATCCCTGCACCCTCTAACAGTGTAGATGAGCCCAAATGTCCTTTCATAAAC-GGCCAATGGCTGTGACGACCCAG
hedgehog     ----------------------------------------------------------------------------------------------------
shrew        ----------------------------------------------------------------------------------------------------
armadillo    ----------------CAGTTGGCCAAACTG---CCCTCTAGAAACACAACTGAAGCCCACTCTTACT---------CCCACAT-------------CAT
elephant     TTCCAGAAACACTATCC-----------------------------------------------------------------------------------
tenrec       ----------------------------------------------------------------------------------------------------
monodelphis  ----------------------------------------------------------------------------------------------------
platypus     ----------------------------------------------------------------------------------------------------
chicken      ----------------------------------------------------------------------------------------------------
xenopus      ----------------------------------------------------------------------------------------------------
tetraodon    ----------------------------------------------------------------------------------------------------
fugu         ----------------------------------------------------------------------------------------------------
zebrafish    ----------------------------------------------------------------------------------------------------
```

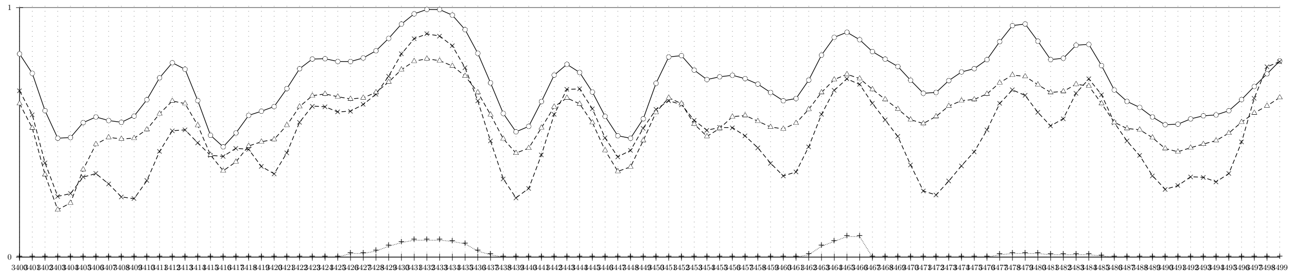

```
human        GGGAATTTGAAGAAACTACTGGCCCTGGCAGCAGCAAGATATCCTGAACCTAGTGCCTGGTAACAAGTAGGAAAAAAAAAAATCTGTCCGCTGCATTATG
chimp        GGGAATTTGAAGAAACTACTGGCCCTGGCAGCAGCAAGATATCCTGAACCTAGTGCCTGGTAACAAGTAGGAAAAAAAAAAATCTGTCTGCTGCATTATG
colobus      ----------------------------------------------------------------------------------------------------
baboon       GGGAGTCTTAAGAAACTACTGGCTCTGGCAGCAGCAAGACATCCTGAACCTAGTTCCTAGTAACAAGTAGG--AAAAAAAAATCTGTCTGCTGCACTATG
macaque      GGGAGTCTTAAGAAACTACTGGCCCTGGCAGCAGCAAGACACCCTGAACCTAGTTCCTAGTAACAAGTAGG--AAAAAAAAATCTGTCTGCCGCATTATG
dusky titi   ----------------------------------------------------------------------------------------------------
owl monkey   ----------------------------------------------------------------------------------------------------
marmoset     GGGGATTTTAAAAAACAACTGGCCCTGGCAGCAGCAAGGCATCCTGAA-CTAGTTCCCGGTAACAAGCAGG-AAAAAAAAAATCTGTCTGTGGCATCATG
mouse lemur  ----------------------------------------------------------------------------------------------------
galago       GGGAATTTTAAGAAACCGCTAGCCCTG---GCAGCAAGACATCCTGACTCTAGTTCCTGCTCACAAGTAGGAAAAAAAAAAACCTGTCTGCTGCATCATG
rat          ----------------------------------------------------GTACCTGTTA-----CAGCAAAAAGAAAAGTC--CCTAATGCATTATG
mouse        --------------------------------------------------------TTGTTA-----CAGCAAAAAGAAAAGTC--ACTAATGTATTAAG
rabbit       GGAGCTGCGAGGAAACCTCCAGCCCCGGCAGCAGCAGGATGTCCT-ACCCTGGTTCCTGGCAACCAGCAGTGGAAACA-----CTGTCTGCTGTGT----
cow          GGGAATTCTGAGAAATGCCCAGCCCTGGAAGCAG-AGGGCATCCCACGCA------CTGGCCACAAGCAGG------A----CCTGGGCACGACACCACG
dog          GGGAATTCTGCGAACT-CCTAGCCCTGGCAGCAGCAGCAGTTCCCACAGG--G---CTGGTCCCAAGCAGG------A--------------AAGGCACG
rfbat        GGATATCCTGAGAAACTCCGAGCCCT-GCGGCAGCAGGACACCCCACATG--GGACCTGGCAACAAGCAGG------A-----CTGTCCACTGCACCAGG
hedgehog     ----------------------------------------------------------------------------------------------------
shrew        ----------------------------------------------------------------------------------------------------
armadillo    GGTCCTTCCATGAAACCCCTGCCTCTGGCAGCAGCAGGAAACCACGCCC--A--ACCTGGCAACAAGCTGTGGGGTCA------------CCGCA-----
elephant     --------TGAGTAGATCCTCACCCATCAGCA---------TCCCACAC--AGGACTCCGCAGCAAGCGGGAAAAACA----TCTGCCCATTGTACC-CG
tenrec       ----------------------------------------------------------------------------------------------------
monodelphis  ----------------------------------------------------------------------------------------------------
platypus     ----------------------------------------------------------------------------------------------------
chicken      ----------------------------------------------------------------------------------------------------
xenopus      ----------------------------------------------------------------------------------------------------
tetraodon    ----------------------------------------------------------------------------------------------------
fugu         ----------------------------------------------------------------------------------------------------
zebrafish    ----------------------------------------------------------------------------------------------------
```

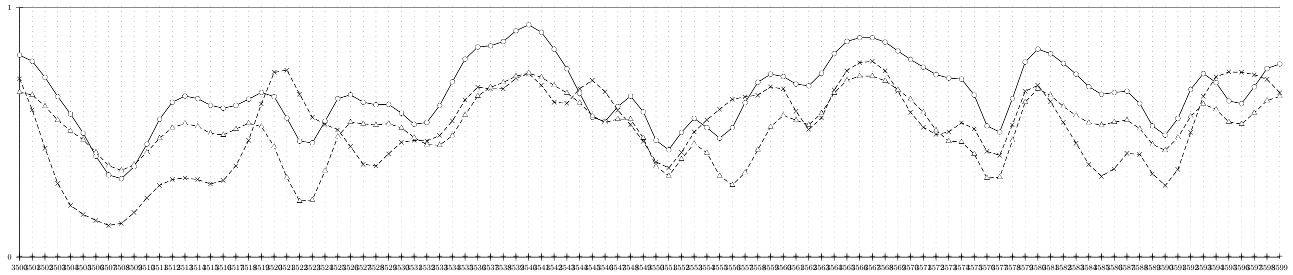

```
human        CAGAGACCACATCTCCACTCTCATGCTGCTCCACCACAGCAGCAATAGAGCGCCCCACACAGACCAGGCTGACCACATACAGGGTGACCGCCACCTGCAC
chimp        CAGAGACCGCATCTCCACTCTCATGCTGCTCCACCACAGCAGCAATGGAGCGCCCCACACAGACCAGGCTGACCACATACAGGGTGACCGCCACCTGCAC
colobus      ----------------------------------------------------------------------------------------------------
baboon       CAGAGACCACATCTCCACTCTCACGCTGC-CCACCACAGCAGCAATGGAGGGCCCCACACAGACCAGGCTGACTACAGACAGGGTGACCACCACCCGCAC
macaque      CAGAAACCACATCTCCACTCTCACGCTGC-CCACCACAGCAGCAATGGAGGGCCCCGCACAGACCAGGCTGACTACAGACAGGGTGACCACCACCCGCAC
dusky titi   ----------------------------------------------------------------------------------------------------
owl monkey   ----------------------------------------------------------------------------------------------------
marmoset     CAGAGACCACTT------------------CTACCACAGCAGTGACGGAGG--CCTGCGCAGACCAGGCTGACCACACACAGGGTGGCCACC----GCAC
mouse lemur  ----------------------------------------------------------------------------------------------------
galago       CAG------CACCCCTGTCCCCACGCTGCTCTACCACAGCAG---TGAAGACCCCCCCCACAGATCAAGCTGAC-------------ACCATC--------
rat          CAAA--CAGTCTATCTCCTCCTGTCATGCGCTGCC-CAACAGCAACGGCGCCCC---CCCAGCCCAGGCTGG-CACGTACAGTCTC-CTTCCCCTCGCAC
mouse        CAAATGCAATCTCTCTCCTCCCCTCACGCTCTGAC-CAACAGCAACAGAGCCAC---CACGGCCCAGGCTGACCACAGACAGTCTCACTTTTACTCAGAC
rabbit       ------------CTCCTCCCCAGAACTACTCC----------------------ACTCCCAGGCCAGGCGGACCACACACAGGGGC-CCTGCACCTGCAC
cow          GGAAGGAGGTGTGTCCTCCACGGGGCTAG-CCACC------------------------CGGCCCCGGCTGACCACCTCCAGGG--ACCCGTACCCGCAG
dog          CAATGAAGTTAT---CTCCACGGTGCTAC-GCGCA-------------------GCGCACGGACCAGGCTAATA---TACAGGGTGATCAGCACCTACAC
rfbat        CAAAGAGAGCATCTTCTCTACGGGGCTGC-CTGCA-------------------CTGCACAGGCCAGGCGGGCAGCAGACAGGGTGACCACCGCCCACGC
hedgehog     ----------------------------------------------------------------------------------------------------
shrew        ----------------------------------------------------------------------------------------------------
armadillo    ---------------------------------------------------------C--AAAGCAGGCTGACCACCAACGGTGACGC------------
elephant     CAATGAGCATGTCCCTTCTCCGGAGCTTT---TCCACGGCAGCAACAGAGTGGCCTGCACAGACCAGACTGAC-ACCTACATGGTGATCACCACCCACAC
tenrec       ----------------------------------------------------------------------------------------------------
monodelphis  ----------------------------------------------------------------------------------------------------
platypus     ----------------------------------------------------------------------------------------------------
chicken      ----------------------------------------------------------------------------------------------------
xenopus      ----------------------------------------------------------------------------------------------------
tetraodon    ----------------------------------------------------------------------------------------------------
fugu         ----------------------------------------------------------------------------------------------------
zebrafish    ----------------------------------------------------------------------------------------------------
```

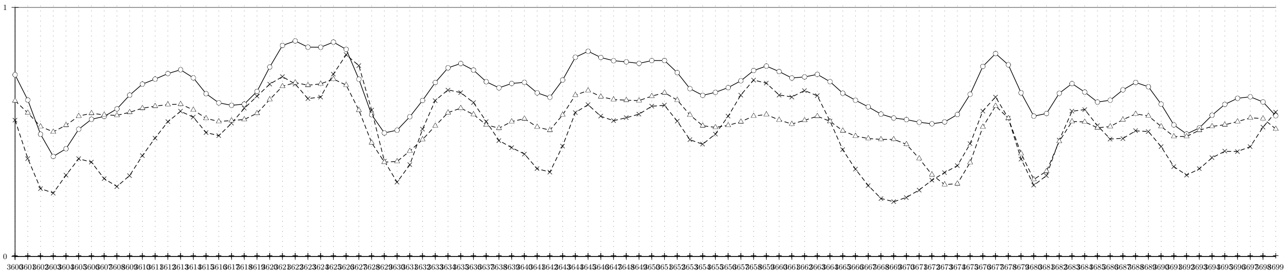

```
human        TCTCCTCCTAAATCGTCCCGGCAGTCACGTTCCAGGGGCAGTCCAGATCTCCCATCCTGGGAGGGAGAAGAAGGGCTGGAGACCCAAACACCCTGAGTAC
chimp        TCTCCTCCTAAATCGTCCCGGCAGTCACATTCCAGGGGCAGTCCAGATCTCCCATCCTGGGAGGGAGAAGAAGGGCTGGAGACCCAAACACCCTGGGTAC
colobus      ----------------------------------------------------------------------------------------------------
baboon       TCTTCTCCTAAATGGCCCCGGCAGTCACGCTCCAGGGGCAGTTCAGATCTCCCACCCTGGGAAGGGGAAGAAGGACTGGAGACCCAAACGCCCTGAGTAC
macaque      TCTTCTCCTAAATGGCCCCGGCAGTCACGTTCCAGGGGCAGTTCAGATCTCCCACC-TGGGAGGGGGAAGAAGGACTGGAGACCCAAACGCCCTGAGTAC
dusky titi   ----------------------------------------------------------------------------------------------------
owl monkey   ----------------------------------------------------------------------------------------------------
marmoset     TCTTCTCCTAAATGGGGCCGG-AGTCACGTTCCTGGGGCAGTTCAGATCTCCCACCCTGGGAGGGAGGAAAAGTGCTGGAGACCCAACCACCCTGGGTAC
mouse lemur  ----------------------------------------------------------------------------------------------------
galago       ------CTTAAATGGCCCCAGCAGTCACGTTTTGGAGGGAGCATAGGTCCCCCACCCTGGGAAGGGGTAAGAAAGGTGGAGACCTGACCACACTGACCAC
rat          CC----------------------TCACCTGCAGCATGAAGCTCAGTTCCCCTGTCCCGGCTG--AGACAGAAGAGTTAGTCACCAGCCACTTAGGCTCC
mouse        CC----------------------TCACCCGTAGCACGAAGCTCAGTTCCCCTGCCCTGGCTG--AGGCAGAAGGGTTGGTCACCAACCACTTAGGCTAT
rabbit       CC----------------------TCACGTCCCGGAGGAAGCTCAG-ACCCCTGCCTCCTTCG----------GAGTGGACAGCTGACCACCTAGGCCAC
cow          CTGCTCGT-GAGTGGTCTCAGCAGCC-TGCCCTGGAGGGGACCCAGGTCT-CCATGCTGGGAGGGGGGGAAACGGTTG---------------CAG----
dog          CTTGTTGTTAAATGGTCCTGGCAATC---ATCTGGAGAGAGCTTAGGTCTTCCACCCTGGGAGGTGAGAGACAGGCTGGACACCCGACCACATCAG----
rfbat        CCTGCTCT-TAGTGGTCCTGACAGCCATGTTCTGGAGGCAG-GCAGGTCTCCCACCCTGGGAGGGAGGAGAAGGGCTGGAGCCCCGACCACACTGG----
hedgehog     ----------------------------------------------------------------------------------------------------
shrew        ----------------------------------------------ATCACCTAACTAGGGAAGGGGGAGAAGGACTGGAAACCCAA-------------
armadillo    ------------------------------------------------------------------------------------------------CTAC
elephant     CT-TCTCCCAAACGGT-CTGGCAGTCACCTTCTGGAGGAAGTTCAG-----------------------GGCTGTCTGGTCCCCTAGATGTTCTAG----
tenrec       ----------------------------------------------------------------------------------------------------
monodelphis  ----------------------------------------------------------------------------------------------------
platypus     ----------------------------------------------------------------------------------------------------
chicken      ----------------------------------------------------------------------------------------------------
xenopus      ----------------------------------------------------------------------------------------------------
tetraodon    ----------------------------------------------------------------------------------------------------
fugu         ----------------------------------------------------------------------------------------------------
zebrafish    ----------------------------------------------------------------------------------------------------
```

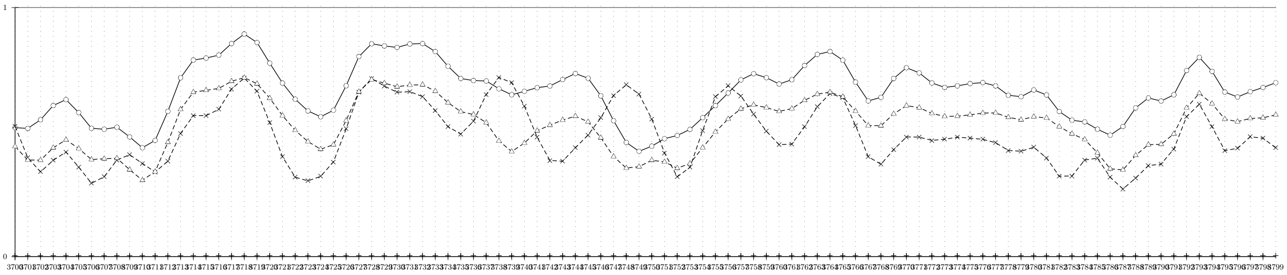

```
human        AGCACCCTGCTACTCCTCCTCAATAACTCCCAGAGTTCCGGCCAGGACATGGGACTGCTCCTCACCTCCAGACAGGCCAGGATTCCAGGGAATCTTACCG
chimp        AGCACCCTGCTACTCCTCCTCAATAACTCCCAGAGTTCCGGCCAGGACATGGGACTGCTCCTCACCTCCAGACAGGCCAGGATTCCAGGGAATCTTACTG
colobus      ----------------------------------------------------------------------------------------------------
baboon       AGTACCCTGCTACTCCTCCTCAATAGCTCCCAGAGCTCCGGCCAGTCCACAGGACTGCTCCTCACCTCCAGACAGGCCAGGATTCCAGGGAATCTTACTG
macaque      AGTACCCTGCTACTCCTCCTCAACAGCTCCCAGAGCTCCGGCCAGTCCACGGGACTGCTCTTCACCTCCAGACAGGCCAGGATTCCAGGGAATCTTACTG
dusky titi   ----------------------------------------------------------------------------------------------------
owl monkey   ----------------------------------------------------------------------------------------------------
marmoset     CACACCCTGCTACTCCTCCTCAATAACTCCCAGAGTTCCGGCCA-------GGACTGCTCCTCACCTCGAGACAGGTCAGGATTCCAGGGAATCTCACTG
mouse lemur  ----------------------------------------------------------------------------------------------------
galago       CACACCC-GACACTCCTCCTCAATAGTTCTCCGAGTTCCCACCAGGATGCGGGACCGCTCCCCACCTCCAGATGG----CGTTACCCAGGGATCTT----
rat          AGTACATGGGGATTCCCC--TAAGGACTCCC------------AGGACATAAGGGT-------------------------------ATGACTCTGGCTG
mouse        AGCACATGGGGACTCTCC--CAAGGACTCCCAGTGCTCTT-CTAGGACATAAGGGT-------------------------------AAGATTCTGGCTG
rabbit       GGCACCATGCGCCTCCTC--CTCCAACTCCCAGAGTTC-----TGAGCGTAACTGC-------------------------------GGGGATCTTGCTG
cow          -GCCCCATGCTCTTCCTC--CCACGAGTCTCAGGGCCCCAGCTGAGATGG-CAGCCACTCCTCACCCACAGACAGGGCTTGACCCCGGGGG-TCTCACTG
dog          -GCACCACGTCATTCCTC--CAACAACTCCCAGAGCTCCAGCAAGGAG-------CTCACCCCACCTGCAGACAGAGCTGGATTCTAGGGGGTCATACAG
rfbat        -ACACGCTGACATTCCTC--CAACAGCTC-CAGAGCTACAGCCAGGACAC-GGACCACTCGTCACCTGCAGGC----------TCTGTGGA-TCTCACCG
hedgehog     ----------------------------------------------------------------------------------------------------
shrew        ----------------------------------------------------------------------------------------------------
armadillo    GGTGCTC-ACCGCCC--------CACCTCCAAGAGGTCTG-CCAGGACGTGGCACCGCT---CACCTCCTGCCAGGGCATGACTCCGGTGCCTCTTCCCA
elephant     -GGCCCACACTCTTCTCCCTCAGCAGCTCCC-GAGCTCCGGTCAGGACACGGACCTGCCCCTCCACTTGAGACCCGGCATGATCTCACTGGCTCTTCCTG
tenrec       ----------------------------------------------------------------------------------------------------
monodelphis  ----------------------------------------------------------------------------------------------------
platypus     ----------------------------------------------------------------------------------------------------
chicken      ----------------------------------------------------------------------------------------------------
xenopus      ----------------------------------------------------------------------------------------------------
tetraodon    ----------------------------------------------------------------------------------------------------
fugu         ----------------------------------------------------------------------------------------------------
zebrafish    ----------------------------------------------------------------------------------------------------
```

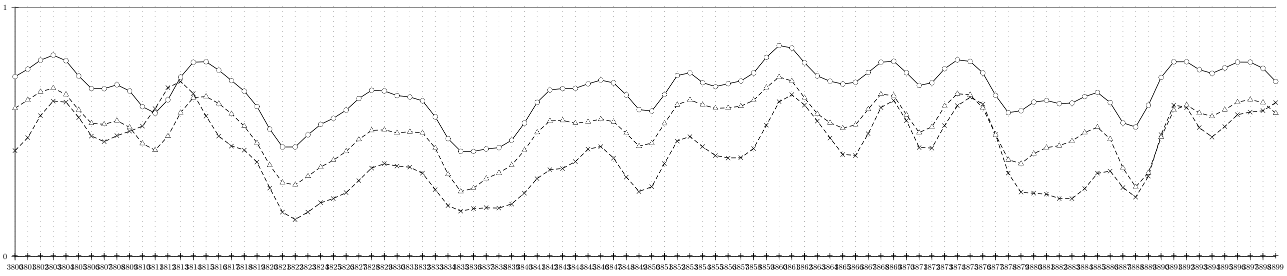

```
human        AGCAACTGCCATGTGCTGAGCTAAGTGCTGCCATTTATCATCTTAGTGAATCTCTTGATAAAATGCAAGGGGCCCTGGCAGATTAAATGATGCCAAGCCT
chimp        AGCAACTGCCATGTGCTGAGCTAAGTGCTGCCATTTATCATCTTAGTGAATCTCTTGATAAAATGCAAGGGGCCCT------------------------
colobus      ----------------------------------------------------------------------------------------------------
baboon       AGCAACTGCCATGTGCTGAGCTAAGTGCTGCCATTGATCATCTTACTGAATCTCTTGATAAAACGCAAGGGGCCCTAGCAGATTGAATGATGCCAAGCCT
macaque      AGCAACTGCCATGTGCTGAGCTAAGTGCTGCCATTGATCATCTTACTGAATCTCTTGATAAAACGTAAGGGGCCCTAGCAGATTAAATGATGCCAAGCCT
dusky titi   ----------------------------------------------------------------------------------------------------
owl monkey   ----------------------------------------------------------------------------------------------------
marmoset     AGCAACTGCCATGTGCTGAGCTAAGTGCTGCCATTGATCATCTTACTGAATCTCTTGGTAAAATGCAAGGAGCCCTAACAGATTAAACAATCCCAAGCCT
mouse lemur  ----------------------------------------------------------------------------------------------------
galago       AGTAACCGTCTCATGCTAAGCCAGGTACTGTCATTTATTGTCTCACTGACTCTCTTGATAAAACGCAAGTGGCCCTAACAGATTAAAGGATGCTCAGCCT
rat          A-CAACTGCCACATGCTACCATAGG--------------------------------ATAAAACACTAATGCTCCTAACAATGTA------GACAAGCTT
mouse        AGCGACTGCTATATGCTACCACAGG-ACAGTCA-TTACTATCTTTCTGAAGCTCTTAATAAAACACTAATGTTCCTAATAACT--------GGCAAGCCT
rabbit       AGCAACTGTCCCGTGCCAAGGCAGGGGCTGCCACTCACCAAGCTCCTGAACCTCCCGCCCAAATGCAAGTGGCCCTAATGGGTCACATGACGCCAAGCCT
cow          GACAACGACCCTCT--------CTGCGCTGCCATCCATGGTCTTGCGAAAA-----------AAGCTGGTGGCCCTAATGGATGAAACCATGCCAAGCCT
dog          AGCAATTGCCATGTGCTAAGTGTGGCGCTACTATTCCCTGTCTTACTGAAT--------AATATGCAAGTGCTCTCAACTAACTCAACTAAGCCCAGCCT
rfbat        AACAACCGCCATGTGCTAGGCCTAGGGCTGTGACTTACCACCTTACTGAGT--------AAAATGCAAGTGGCCATAAAAGATTAAATTATGCC-AGCCC
hedgehog     -----------------------------------------------------------AAAGTGTAAGTGATCCTAAAAGATAAAATGGTGCCAAGCTT
shrew        ----------------------------------------------------------------------------------------------------
armadillo    GACAACCACCAAGCACTAAGCTCATTTGGACACCGGGCGAATCACGGGAGTCTCCGGACAAAATGCCAATGGCCCTAAGAGGTTAAACTCTGCCAAGCCT
elephant     AACACCTGCCATGTGACACGCACAATGGTGCCTTTTAC--TCTTACTAGTTAC--------------------------AGAATCCATTATGCCCGCACC
tenrec       ----------------------------------------------------------------------------------------------------
monodelphis  -------------------------------------------------------TGATGAA--GGGAATGTTCCG--CGGATTAAGTTTTGCTTATTCT
platypus     ----------------------------------------------------------------------------------------------------
chicken      ----------------------------------------------------------------------------------------------------
xenopus      ----------------------------------------------------------------------------------------------------
tetraodon    ----------------------------------------------------------------------------------------------------
fugu         ----------------------------------------------------------------------------------------------------
zebrafish    ----------------------------------------------------------------------------------------------------
```

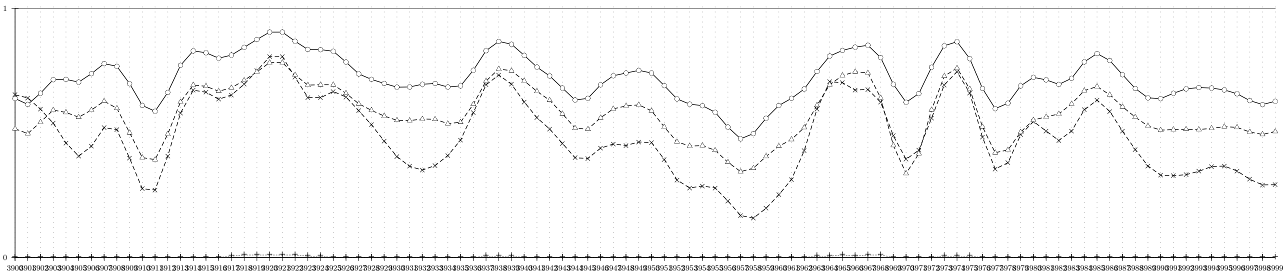

human	CACTCTAGGGACACAGCCACCAAAGGTGGTCAAGTTGGAATTCTCTGAAGCCCTGTTACTGATGTTCTCGTAACACAGGTCTAAGCCACCAAAGAAACT
chimp	--
colobus	--
baboon	CACTCTAGGGACACAGCCACCAGAAGGTGGTCAAGCTGGAATTCTCTGAAGCCCTGTTACTGCTATTCTCATAACATGGGGCTAAGCCACCAAAG-AACT
macaque	CACTCTAGGGACACAGCCACCAGAAGGTGGTCAAGCTGGAATTCTCTGAAGCCCTGTTACTGATATTCTCATAACACGGGGCTAAGCCACCAAAG-AACT
dusky titi	--
owl monkey	--
marmoset	CACTCTAGGGTAACAGCCACCAGAAGGTGGTCAAGTTGGAATTCTCTGAAGCCCTGTTACTGATATTCTCATAACACAGGGCTAAGCCACCAAAGAAACT
mouse lemur	--
galago	CACTCTGGGTGGACAGC--------------TTGGCTAGAATTCTCTGAAGGCCTGCGACTGATACTCTGGCAACACAGGGCTGAACTACCAAAGTAATT
rat	TGTTCTAAGGAGGCAGTCACCAGAAGGTGGTCAAACTGAAGCTCCCTGGTGCCTTC------GTATCCTGATAACATGGAGTACAGCCATTAATGTATCT
mouse	TGCTCCAAGGAGGCAGTCACCAG-AGGTGGTCAAAGTGAAGCTCCCT--TACATTCTG--TAATATTCTAATAACATGGAGTACAGCCATCAATTTATCT
rabbit	CTCTCCAGAGAT---GGCCCCAGGAGGCGGCCAGGCTGGAACTCCCTGGAGCCCAGTGACGGACATTCTGGCACCACATGGCACAGCCACCGAGGCGGTG
cow	CACTCTAGGGAGACCGGCACCAG------------CCGGAATTCTCTGAAGCTCGATTTCTGATATCCTGCTCACACCAGGCA------TTAAA------
dog	GGCTCTAGGGTGACAGCCACCAGAAGGTGGTCCAGTTGGAATTCCTTGAAGCTCTATAAGTGATATTCTGTTAACATTTGGTAAAGCTATCGAAGTAACT
rfbat	TGCTGGAGGGAGACAGCCAACAG---GTGACCCAGTTGGAATTCCTTGGAGCGCTATTACTGATATTCTGGTGACACTAGGCAAAGCCACCAAAGTAATT
hedgehog	--TTTTTCGAGTCTGACTGGCGCTATTCTGCTAACACCAGGCAGAGCCAGAACA------
shrew	--
armadillo	CGCTCTAGAG--GCAGCCACCAGAAGGGGGCCAAGCTGGGACTCCGTGACGCCCTGGGACTAA-ATGCTGGTAACACT-----GAGCTGCCAAGGGAATT
elephant	CATTCTGGGGGT-CAGACACCAGCAGGCGGTCAAGCTGGAATTCTGTGAAGCCTTATATCTGCTATTCTG-TAACGCTGGGCAGAGGCACCACAGTAACT
tenrec	--
monodelphis	CAATAAAGGAAAAAAGACAACAGGATAGTACCCAGTTGGAAAGTTTAAAAGGTCTGTCTTA---ATTCTAATAACACAGCATG-----------------
platypus	--
chicken	--
xenopus	--
tetraodon	--
fugu	--
zebrafish	--

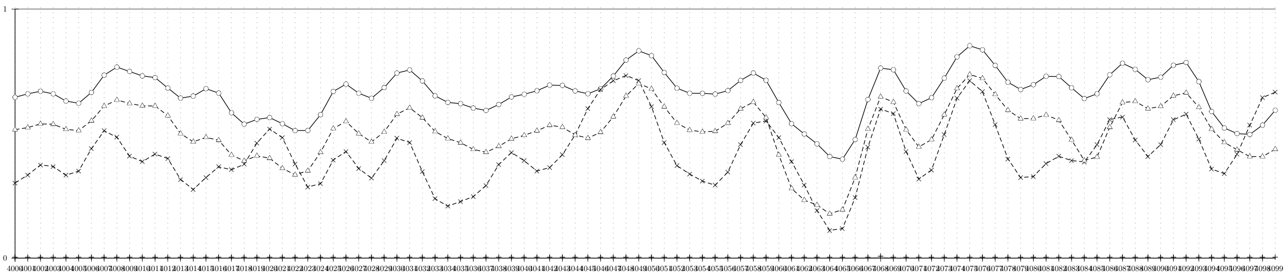

human	AACTTTGTTTTTTAAAACGACATCTCCCAAGGAAATCCAGGTAGCCAAGGCTATGAGTCTGAGTAGGTTCCATAGCCATAAAGCCTCCTTTGTTTGCCCG
chimp	--
colobus	--
baboon	AACTTCATTTTTTAAAACTACAGCTCCCAAGGAAATCCAGGTAGCCAGGGCTATGAGTCTGAGTATGTTCCAGAGCCATGAAGCCTCCTTTGTTTGCCCG
macaque	AACTTCATTTTTTAAAACTACAGCTCCCAAGGAAATCCAGGTAGCCAGGGCTATGAGTCTGAGTATGTTCCAGAGCCATAAAGCCTCCTTTGTTTGCCCG
dusky titi	--
owl monkey	--
marmoset	AACTTTATTTTTTAAAACAACAGTTCCCAAGGAAAGCCAGGTAGCCAGGGC--TGAGTCTGAGTATGATCCAGAGCCATAAAGCCTCCTTCGTTTGCTCA
mouse lemur	--
galago	AACTTTATTT----AAAATGCAATCCCTTCG-----CCAGGTAGCCAGGGTTATGAACCTGGGCACATTCTGGAGCCACACAGTCTCCTCTGTTTGCTTG
rat	GTCTATGTTAC--AAAAATACAAGTACCAAAGAG--------AATCAGGGCTGTGAATCTGTGTGCTTTCGAAAGCCATAAAGCTTCCT-----------
mouse	GTCTATGTTAG--AAAAATATAAATACTAAGAAA--------AATCGGAGCTATGAATCTGCGTGCTTTCAAAAGCCATGAAGCCTCCT-----------
rabbit	AGCCGTATT---TTACAACACACTAGCCACGGAAGGCCAGGCAGCTGG----------------------------------GCCGCCTCTGTTCACCCT
cow	-------------AAAAACACAACTACTAAGAGCAGCTCGGTAGCCAGGGCTATGAATCTGCGTCCTTTCTGGAACTGTAAAGCCTCCCTTATTTTCGTG
dog	AACTTTATTTTTTGTAACTATAATTACAAAGGAAAAGCAGGTAGCCAGGG-TACGTATCTACCCATGTTCTGAAACCATAAAGCCCCCTTTGTTCATACG
rfbat	AATTTTATTTTTTGTAATTACTATTACTAAGGAAGGCCAGATAGCCAGGGCAATGAATCTGTGTATGCTCGGGACCCATAAAAGGCCCTTTGTTCGCACG
hedgehog	-------------GCAATTATGATCACTGGGGGGGGGGGAAGAGAGGAGGCTCCATGTCTGTGCCCATTCTGCAGCCACAGACCCACACTCATTTACCCG
shrew	--
armadillo	AACCTGCTCTTTT-AAAACAGGATGACAGCGGAGAGCTGGTGGGGCAAGGCTAAGGGTGCAGGCGCAT-CCGGGGCCACAGTGCCGCCT----------G
elephant	GTCT------------------ATGACTAAGGAAAGCCAG--GGCCAGGGCTATGAGTTTGAGCATGTTCTGGGGCCCCAGAGCCTC-TGTGTGCACACG
tenrec	--
monodelphis	-------------AACACCACC---ACTAAGGAAAGTAAAGTACTTAAGACATTTAATTTGCATTAATTCTAAGGCCACAAAGATGCCTTTGTTT-CCAA
platypus	-------------GGGA---
chicken	--
xenopus	--
tetraodon	--
fugu	--
zebrafish	--

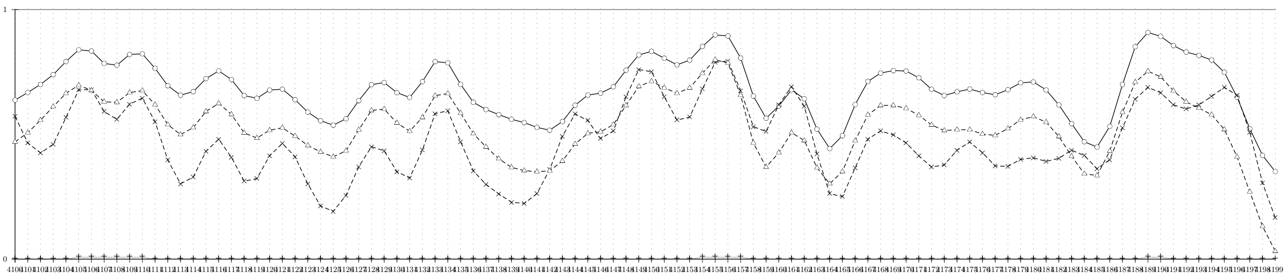

human	ACTCTGCCTCTCTGAAGTCTGCATTTATCCTCTGTGACGGCATTGTTCAGCATTCCAGACGGCATAACAGGAACAACTCTGTTCACTCTTGACCAGTGAT
chimp	--AGTGAT
colobus	--
baboon	ACCCTGCCTCTCTGAGGTCTGCATTTATCCTCTGTTACGGCCTCGCTCAGCACTCCAGACGGCATAACAGGAACACCTCTGTTCACTC-TGACCCGTGAT
macaque	ACTCTGCCTCTCTGAGGTCTGCATTTATCCTCTGTTACGGCCTTGCTCAGCACTCCAGATGGCATAACAGGA-CACCTCTGTTCACTC-TGACCCGTGAT
dusky titi	--
owl monkey	--
marmoset	A---TGCCTCTCTAGGGTCCGCATTTATCTTCTGTTACAGCATCGCTCAGCATTCCAGACGGCAGAACAGGCACACCTCTGTTCACTC-TGACCAGTGAT
mouse lemur	--
galago	AGTCTGCCTCTCTGAGGTATGCGATGGTC--CTGTTGCAGCATTGCTCAGCACTGTAGGTGGCGTAACAGGAACACGGCTGTCCTCCC----CCAGCAGT
rat	-GTCAGCCACTC------------------------AAGGCTGTGCTCAGCATTCCAGATA---------------------------------AGTGCT
mouse	-GTTTGCGTCTC------------------------AAGGTTGTGCT-GGCATCCCAGATGGCCTCAAGGGAACAC-------CAACC------AGTGCT
rabbit	GGTTTGCCTCTCTGAGGCTGGCACTTGTCCT-GGTGAGGGGCTCGCCGGGCA-TCCAGATGGCA-GACAGGAACACCTGTGTTCATTC-TGACCCGTGGG
cow	AGTCCGCGACTCTGAAGTTTGCATTTCTCCTCCGTTAGGCC--CACTCAGCAC-CCAGCTAGTGCAACAGGGACCCTTCTGTCTGCACT-GACCAGCGGC
dog	AGTCT------CTGAAGTTTGCATCCATTCTTTGTTGGGACATCGCTCAGCATTCCAGGGAACATAGCAAGAATACCTCTGTTCATGCT-GACTAGCCAC
rfbat	AGTCT------CTGAAGTTTGCATTTATCCTTTGTTGGTCCAGTGCCCAGTATTCCAGACAGCGTCACAGGAATACCTCTGTTCGTGCT-GACCA-CGGT
hedgehog	CATCT------GACAAGTTGGAGCTGAGCCCGGGTTAGGGCGCTGCCTGGCATCCCAGAGAGT-----AAGAGGACTGGTCCCGGGGCT-GGCTGGCAAG
shrew	--
armadillo	----TG-----CTCCAGCTGGCATTTCTCCTTTGTCAGGGCATCGCTCAGCCTTCCAGGTGGCTCCACAGGGGATCCTCCATTCACCCC-GCTCAGGGAC
elephant	ACTTTGCCTCTCTGAGGTTTGCACGTCTTCTCTGTTG-ACAATTGCTCAGTATTCCAGACGGCACAGCTGGAATACCTCTGCTCATTCT-GACCAGGAAT
tenrec	--
monodelphis	ATTCT------GGAAAGTATTTGTTTATTCACTGTTGGATCATCTTGTAGCATTCCAGAGAGCAAGACTATAATATCACAGTTATTT-TTGGTCAATGCC
platypus	--GGCC------
chicken	--
xenopus	---T-GCCAGTGTA
tetraodon	--
fugu	--
zebrafish	--

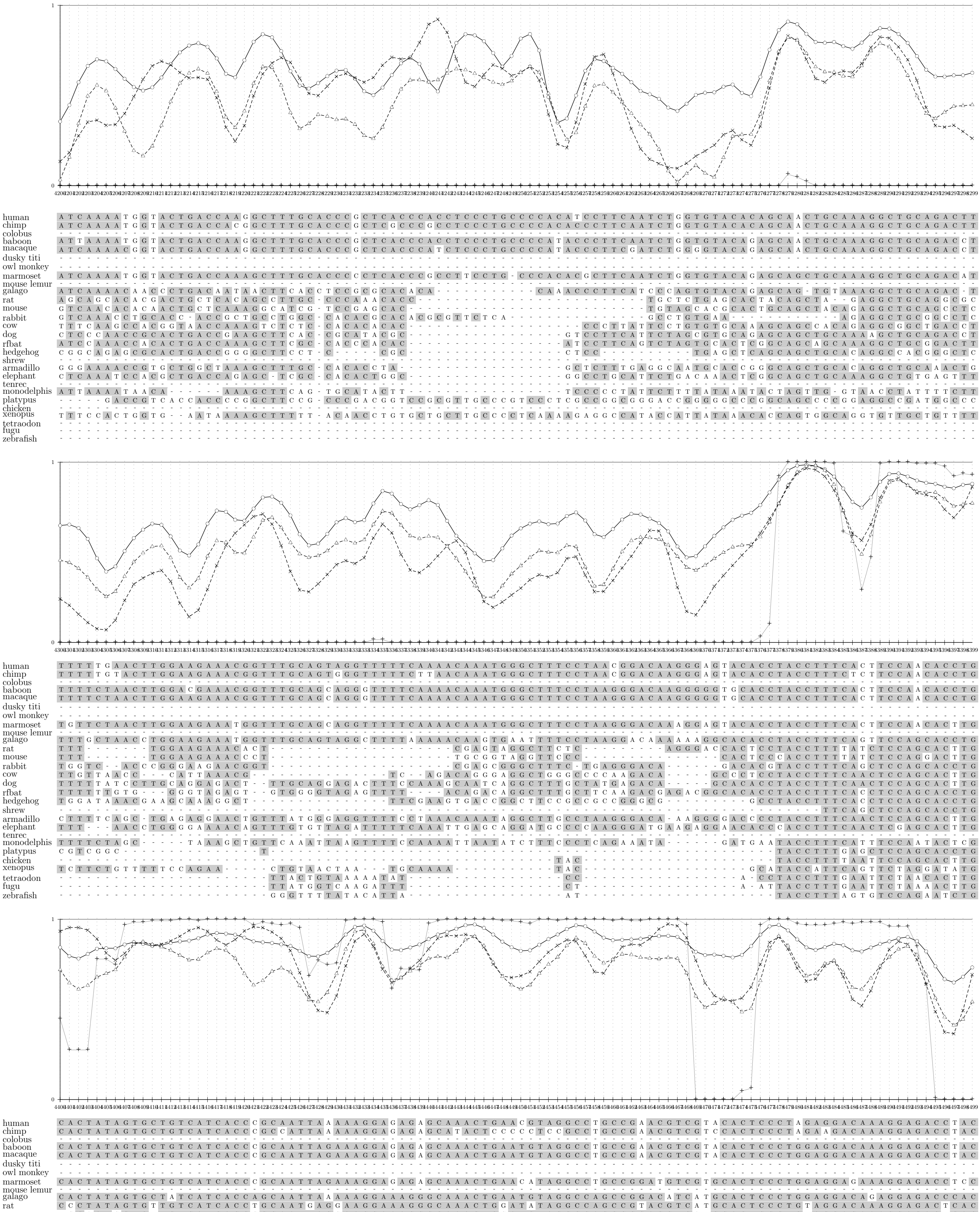
human
chimp
colobus
baboon
macaque
dusky titi
owl monkey
marmoset
mouse lemur
galago
rat
mouse
rabbit
cow
dog
rfbat
hedgehog
shrew
armadillo
elephant
tenrec
monodelphis
platypus
chicken
xenopus
tetraodon
fugu
zebrafish

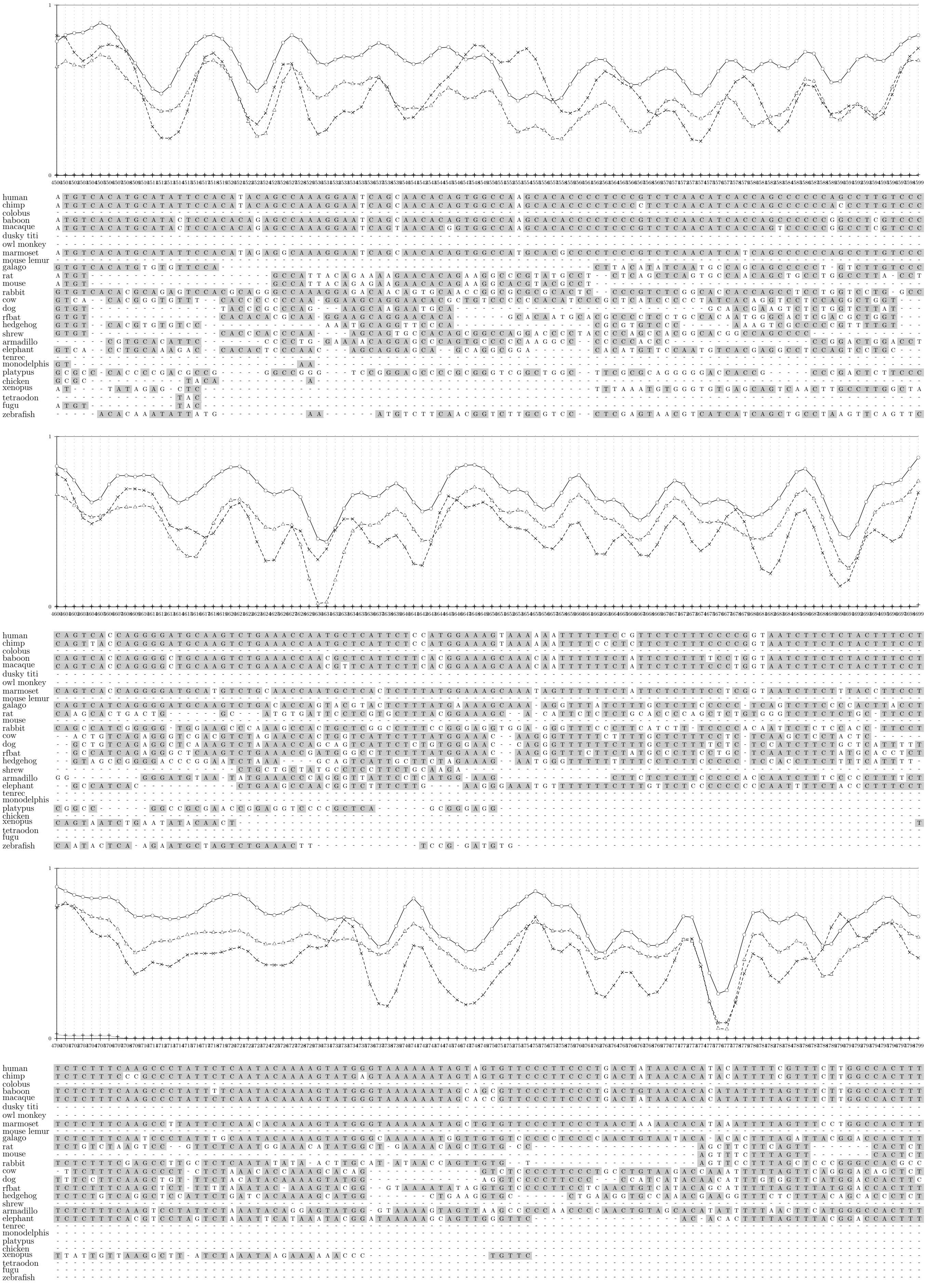
human
chimp
colobus
baboon
macaque
dusky titi
owl monkey
marmoset
mouse lemur
galago
rat
mouse
rabbit
cow
dog
rfbat
hedgehog
shrew
armadillo
elephant
tenrec
monodelphis
platypus
chicken
xenopus
tetraodon
fugu
zebrafish

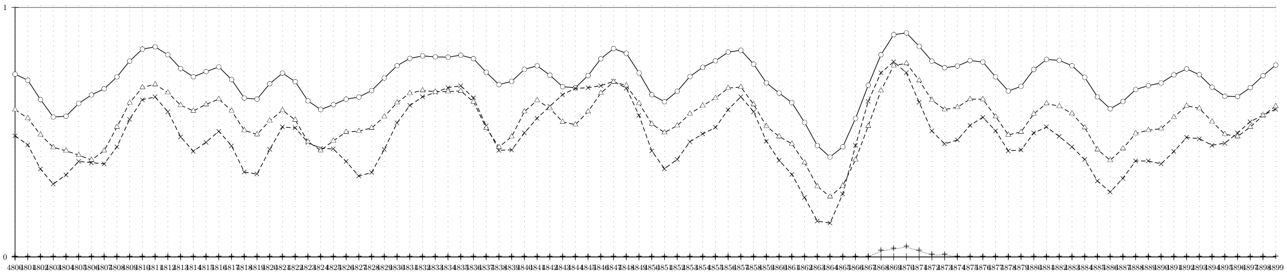

human	GGTGTAGAGCAAGACTCTCAGCCTGCCTAGATCCATCTCCTCTCGCTTGCTGATGAAAATAATCTATGTTTTTGAAAATCCAGAGATGATGAAATGGCTG
chimp	GGTGTAGAGCAAGACTCTCAGCCTGCCTAGATCCATCTCCTCTTGCTTGCTGATGAAAATAATCT--GTTTTTGAAAATCCAGAGATGATGAAATGGCTG
colobus	--
baboon	GGTGCAGAACAAGACTCTCAGCCTGCCTAGATCCATCTCCTCTCGCTTGCTAATGAAAATCATCTATGTTTTTGAAAATTCAGAGATGATGAAATGACTG
macaque	GGTGCAGAACAAGACTCTCAGCCTGCCTAGATCCATCTCCTCTCGCTTGCTAATGAAAATCATCTATGTTTTTGAAAAGTCAGAGATGATGAAATGACTG
dusky titi	--
owl monkey	--
marmoset	GGTGTACAGCAAGACTCTCAGCCTGCCTAGATCCATCTCCTCCCACTTGCTGATGAAAATAACCTATGTTTGTGAAAATCCAGAGATGATGAAATGACTG
mouse lemur	--
galago	GGCATACACCAAGATTCTCAGCCATCCAATATCCATCTTCTTTTGCTTGCTGAAGAAAAAAAATC-TGTTTTTGAAAACCCAGAGGTAATGCAAGGACTG
rat	GGGACAGGGCAAGAACCTTAGCCTGCTGGAATCCATCCCCTCTTGCTTGCTAATGCAAACAATCTATGTTTTAGAAAAGCCAGAGATAATAGAGACA-CG
mouse	AGGATAGGGAAACATCCTTTGCCTGCTGGGATCAATCCCCT-TTGCTTGCTAATGAAAACAATTATTGTTTTAGGAAAGCCAGAGATAATGGAGACA-TG
rabbit	GGTGCAGGACAAGAC--------TGCTTGGACCCATCTCCTCTCACTTGCCGATGGGAAGCATC--TGTTTTTGAAAATCCAGGGAGAAGGGAGGCG-TT
cow	GGTGTATAGCAAGATGCTTGGCCTGTCTACACCCATCTGCTCTCATGTC---ATGAAAATAGTCTCTGTTTTCGAAAATCCATAGATGATGGAAAGGCTG
dog	AGCATAGAGCAAGATTCTCAGCCCATCTAGATCCATCTATT-TCACTTCCCAGTGAAAATA-TACACTTTTTTGAAAATCCAGAGCCAATGGAAAGACCG
rfbat	GGTATAGAGCAAGATTCTCAGTC-GTCTAGATCCATCTATTCTCACTTGCCAATGAAAATAATCTACGTTTTTGAAAATCCAGAGGTAA-AGAAAGACTG
hedgehog	GCTGCATGGCGGGGTTCCTGGCCCACACAGACCCGCCTCCTCTCTCTTG-CAGTGAAAGGAGTGCTTTGTTTGGAAAAGCCTGTGAGGACGGACAGGCCG
shrew	--
armadillo	GGTACAGAGCAAGATTCTTGGTCTTCCTTGATCTGTTTATTCTCACCTGTTGATAAAAATAATCTTTTTTTTT----ATACAGAGAAAATGGAAGGACTG
elephant	GGTACAGAGCAAGGTTCTCAACCCACCTAGATCCATCTACTTTCACTTGCCAACGGAAATAACTTTTTTTTTTTTTAACTCAGAGAAAATGAAA-GGCTG
tenrec	---ACTCTCTTTTTTTGTGGCTGATTGCTAGATAAA-GCCTA
monodelphis	--
platypus	--
chicken	--
xenopus	--
tetraodon	--
fugu	--
zebrafish	--

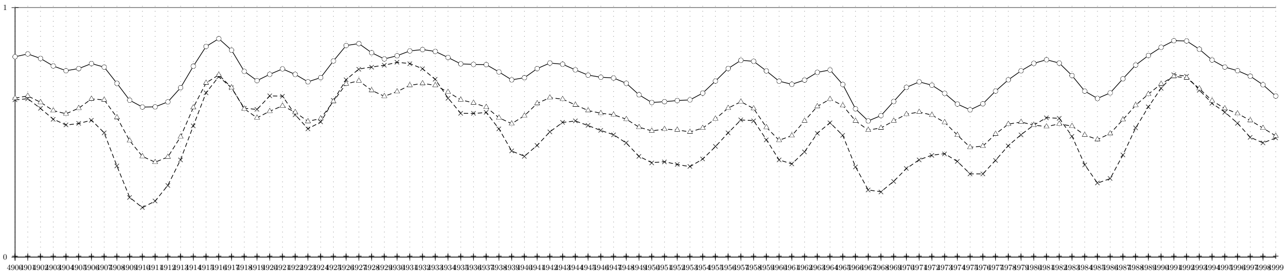

human	GTACTGCTTTTGGTGAGACACCAATGTGGTGATGTCTGGTTTTTATGAGATATTATAAATAACTTATTAGCTCATACCACAAACCAAAGCACAGATGCTG
chimp	GTACTGCTTTTGGTGAGACATCAATGTGGTGATGTCTGGTTTTTATGAGATATTATAAATAACTTATTAGCTCATACCACAAACCAAAGCACAGATGCTG
colobus	--
baboon	GTACTGCTTTGGGTGAGACATCAATGTGGCGATGTCTGGTTTTTATTAGATACTATAAATAACTTATTAGCTCATACCACAAACCAAAGCACAGATGCCG
macaque	GTACTGCTTTTGGTGAGACATCAATGTGGCGATGTCTGGTTTTTATTAGATACTATAAATAACTTATTAGCTCATACCACAAACCAAAGCACAGATGCTG
dusky titi	--
owl monkey	--
marmoset	GTACTGCTTTCAGTGAGACATCAACGTGGTGATGTCTGTTTTTTACTGGATATTATAAATAACGTATCAGCTTGTACCACAAACCAAAGCACAGATGCTG
mouse lemur	--
galago	GTCCTGCTTTTGATTAGACACCGATGTGGTGATGTCTGGTTTTCATTAGGTATTATAAAGAAGTTACTAGCTCATACCAGAAACCACAGCATAAAGGCTG
rat	GTGCTGCTTTTGATGAGAGGCCAATGTGGTGATATCTGGTTTTTATTAGGCATAGTAAATAAGTTACTACATCACACTGAAAACAAAAGTGCAGATGATG
mouse	GTGCTGCTTCTGATGAGAGGCCAATGTGGTGAGATCTGGTT---ATTAGAAATTGTAAATCAGTTATTA----ACACTGAAAAGCAAAGGACAGATGATG
rabbit	GTGCTGCTCCTGCTGAGACACCA--GTGGGGACGTGCAGCCTGTGTTGGACATCAGAAATCACTCATCA-CTCACCCCAGAGACCAGGGCACAGATGCTG
cow	GTGCTGCTTTTGATGAGACACCCATGAGGTGATGTTTGGTTTTTATTAGATGTTAGACAT-----------TCACAGCAGACA------CACAGATGAGG
dog	GTTCTACTTTAGTTGA--CACCAGTGAGGTGATGTCTGGGTTATATTAGGTACTATAAAT-----------GCACTCTGGACATCAAAGCACAGATGCTG
rfbat	GTGCTGCTTTTGATGAGATGCCAATGAGCTGATGTCTGGTTTTTACTAGATATTATAAAT-----------TTACGCCAGACACGGAAGCACAGTTGCTG
hedgehog	CTGCGGTTCTGGATGAGACACCAGCGAGGTGGTGTCTGGGTTTTATTGGGTGTCATGAGT-----------TCACCACACACACACACACACACACACAC
shrew	--
armadillo	GTGCTACTTTTGATGAGATGCCGAAG-GATGATGTCTGCTTTTTATTGGATATTGTCAATAAGTTACCAA-TCATAACAGAAACC-CAGCACAGATGCTG
elephant	GTGCCACTCTTGGCAG------AATGAGACAATGTCCGGTTTTTATTGGACATCATAAACAGGTTATCCACTTGTACCAGACACCAAAGCACAGACGCTG
tenrec	ATGTGAGTCATTTAAG------AACTAGACGATGCTTGGCCCTTTCTG--
monodelphis	--
platypus	--
chicken	--
xenopus	--
tetraodon	--
fugu	--
zebrafish	--

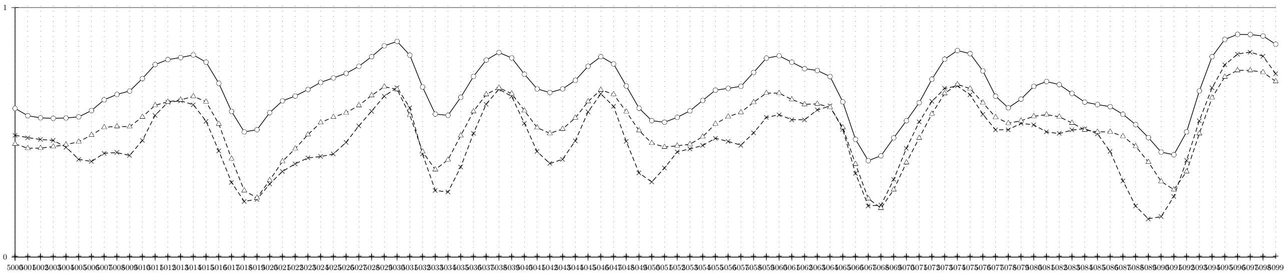

human	TCACGTTACTAACCCTGTTAGGCCTTCCCAGGGTTGTAGCCAGCACTGTCCACATGCATCAGCCACCAACCAGGGCACTGGGCACGTTTGGGGAGGGCCC
chimp	TCACGTTACTAACCCTGTTAGGCCTTCCCAGGGTTGTAGCCAGCACTGTCCACATGCATCAGCCACCAACCAGGGCACTGGGCACGTTTGGGGAGGGCCC
colobus	--
baboon	TCACGTTACCAACCCTGTTAGGCCTTCCCAGGGTTGCAGCCAGCACTGTCCACAGGCATCAGCCACCACCTAGGGCAGTGGGCACGTTTGGGGAGGGCCC
macaque	TCACGTTACCAACCCTGTTAGGCCTTCCCAGGGTTGCAGCCAGCACTGTCCACAGGCATCAGCCACCACCTAGGGCAGTGGGCACGTTTGGGGAGGGCCC
dusky titi	--
owl monkey	--
marmoset	TCACGTCACCAACCCTGTTAGGGCTTCCCTGGGTTGTAGCCAG---------CAGGCATCAGTCA-CACCCAGGGCAGTGGGCACACCTGCAGAGGGTCC
mouse lemur	--
galago	TCATGTCAC-AAACCTACAAGATCTACCTAGGGTTGTAGCCCTCACCGTCCACACGCATCAGACA-AGGCTTGGGCAGCAGGTACCCTGAAGGAGGG-CC
rat	CTATGTTACAAAG---GCATGGACATCCCAGGTC-ACAGCTTGCACTGGCTG------------ACCATGGGAGGC------------------GGGCAC
mouse	CTATGTTACGAAC---ACACGGGCATCT-GGGAT-ACAGCTCGCATTGCTTG------------ACCCAGGGAGGC------------------AGGCAC
rabbit	CGGGGCTGCAAACACTGCAGAGCCTTCCCAGGCTGGCAGCCTGCGCTGCCTGCGGGCTTCAGCCACCGGACGGGGC-----GCACCCCTGGCCAGGGCCC
cow	CCACATTACACACGCAGCATGGCCTTCCCAGGACTGCAGTCTGCACTGTCCGCGGGTGTCAGCTG-GACCCAGGAC----AGCATCCTCAACCAGGGCCC
dog	TCACATTACAAAC-CAGTGTGGCTTTTCCAGGA--------------------GAGTG----------------------GACATCCTCAAGGAGGGCCC
rfbat	TCATATTATAAACCCAGCGTGGCCTTCCCAGGATTGCAATCTGTACTGTCTACGGGCGTCAGCCA----------------------TCAGCCAAGGCCC
hedgehog	ACACACCACACACCCAGAGCGCTCTTCCCAGGACTGCAGTCTGCTCTGTCCACCAGTGTCTGTC-----CCACGACAGTGGGTGGC--CTGATGGGGCCC
shrew	--
armadillo	TTCTCTGACAAACCCAGCTTGGCCATCACAGGACGGCA-CCCGCCCTGCCCCTGGGCACCGGCCATGACCCAGGGCAGCGGGCAACCTCAGGGAGGGCCC
elephant	CTATACCATGAACCCAGCTCAGCCACGCCAGGATCCCGGCCTATACTGTTTGCAGGCCTCAGCCACAGCCCGGGACCACAGACATCCCCAGCGAGGACCC
tenrec	--
monodelphis	--
platypus	--
chicken	--
xenopus	--
tetraodon	--
fugu	--
zebrafish	--

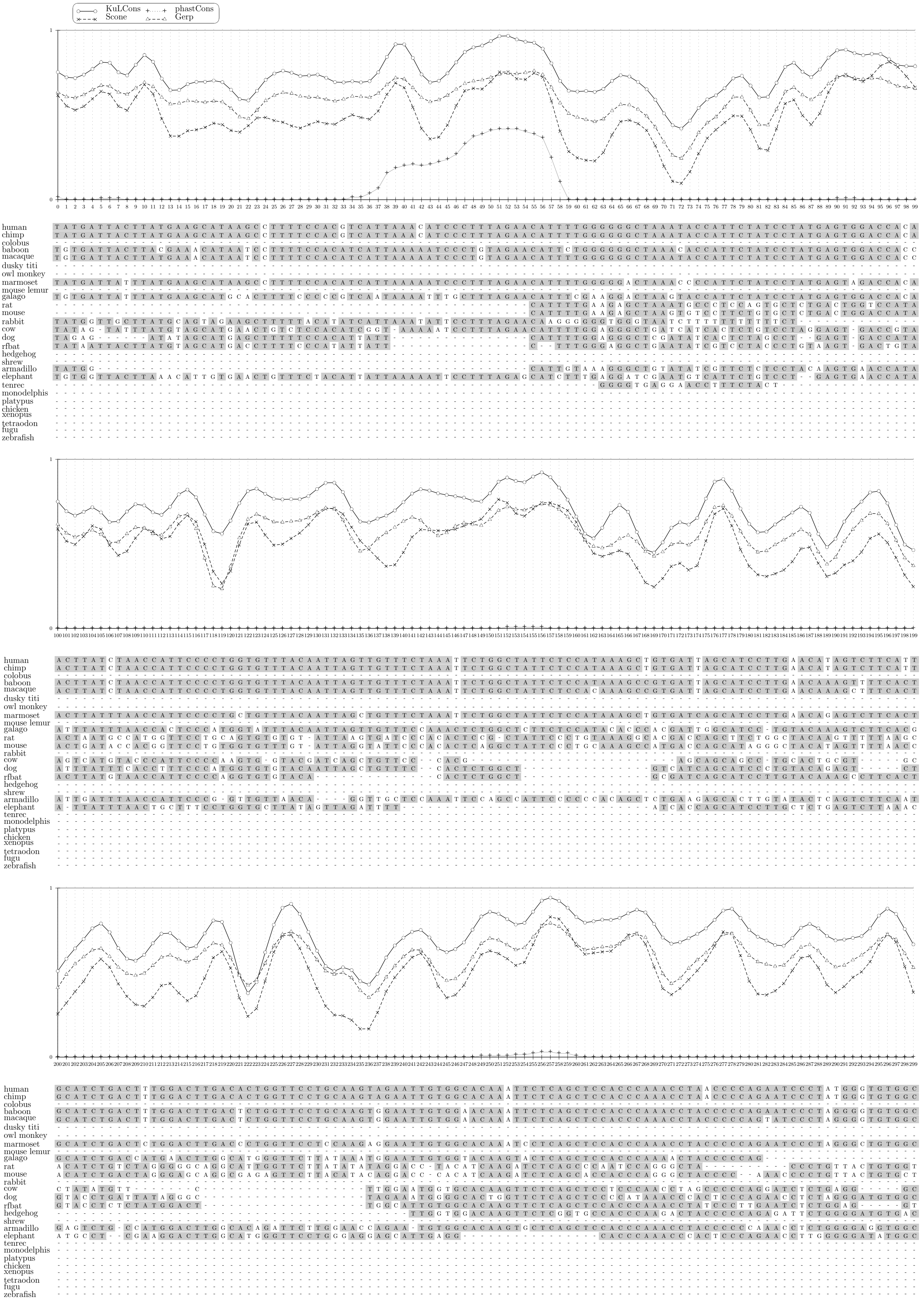
KuLCons
Scone
phastCons
Gerp
human
chimp
colobus
baboon
macaque
dusky titi
owl monkey
marmoset
mouse lemur
galago
rat
mouse
rabbit
cow
dog
rfbat
hedgehog
shrew
armadillo
elephant
tenrec
monodelphis
platypus
chicken
xenopus
tetraodon
fugu
zebrafish

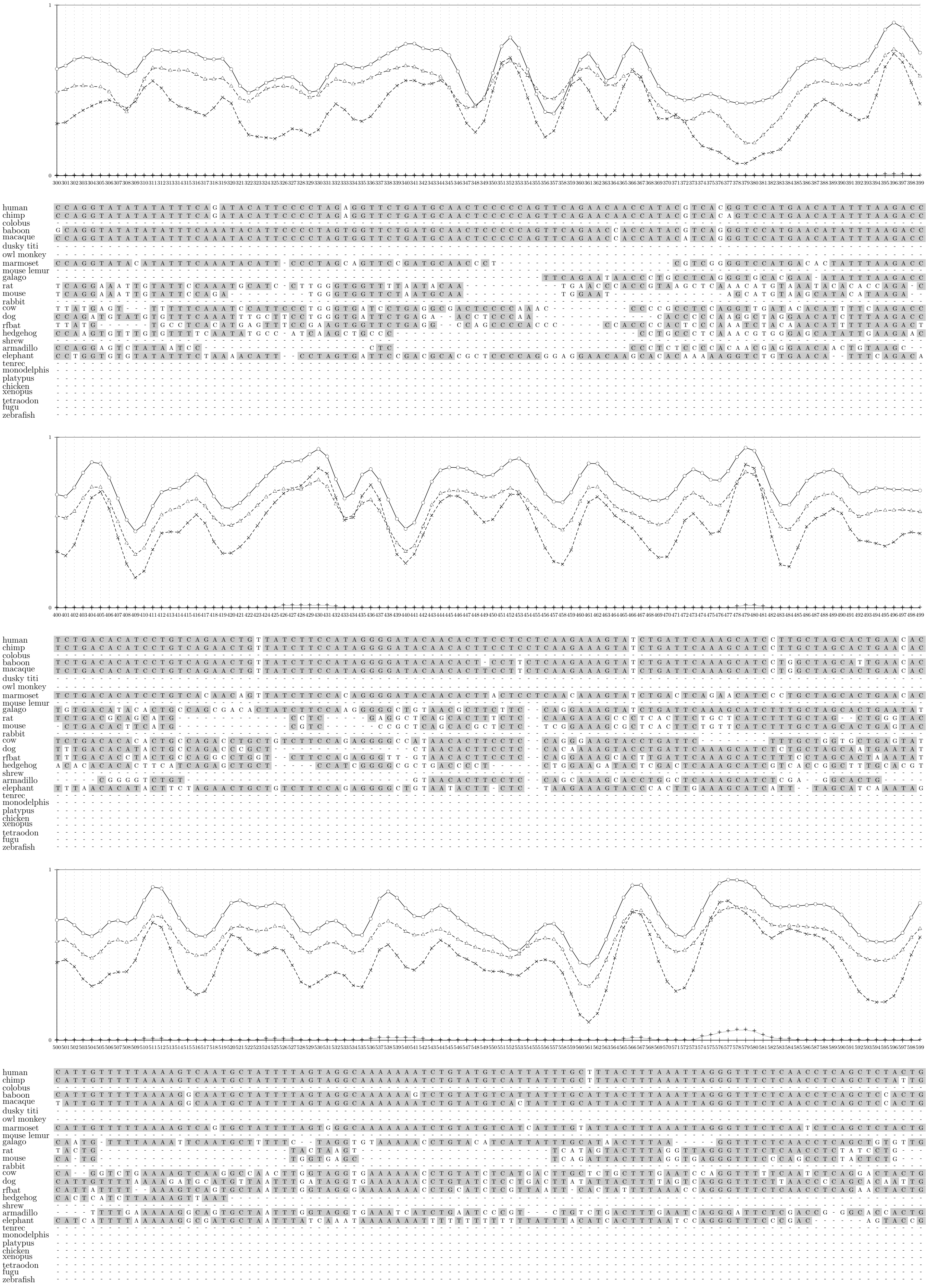

human
chimp
colobus
baboon
macaque
dusky titi
owl monkey
marmoset
mouse lemur
galago
rat
mouse
rabbit
cow
dog
rfbat
hedgehog
shrew
armadillo
elephant
tenrec
monodelphis
platypus
chicken
xenopus
tetraodon
fugu
zebrafish

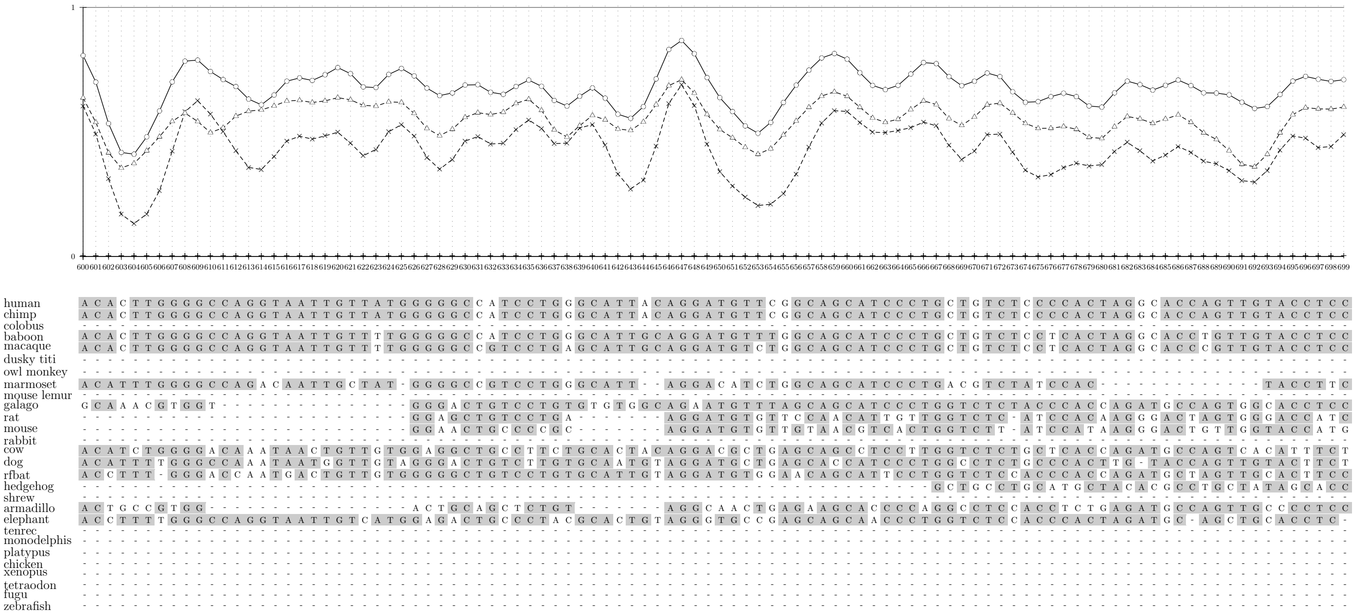
1
0
human
chimp
colobus
baboon
macaque
dusky titi
owl monkey
marmoset
mouse lemur
galago
rat
mouse
rabbit
cow
dog
rfbat
hedgehog
shrew
armadillo
elephant
tenrec
monodelphis
platypus
chicken
xenopus
tetraodon
fugu
zebrafish

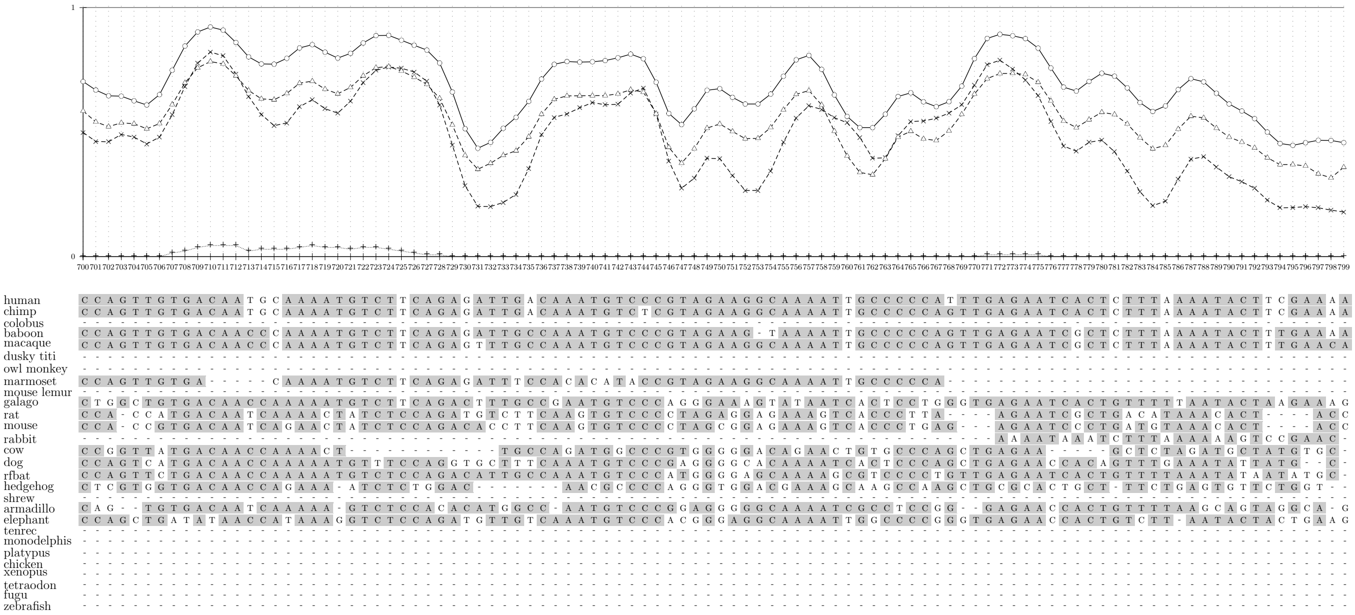
1
0
human
chimp
colobus
baboon
macaque
dusky titi
owl monkey
marmoset
mouse lemur
galago
rat
mouse
rabbit
cow
dog
rfbat
hedgehog
shrew
armadillo
elephant
tenrec
monodelphis
platypus
chicken
xenopus
tetraodon
fugu
zebrafish

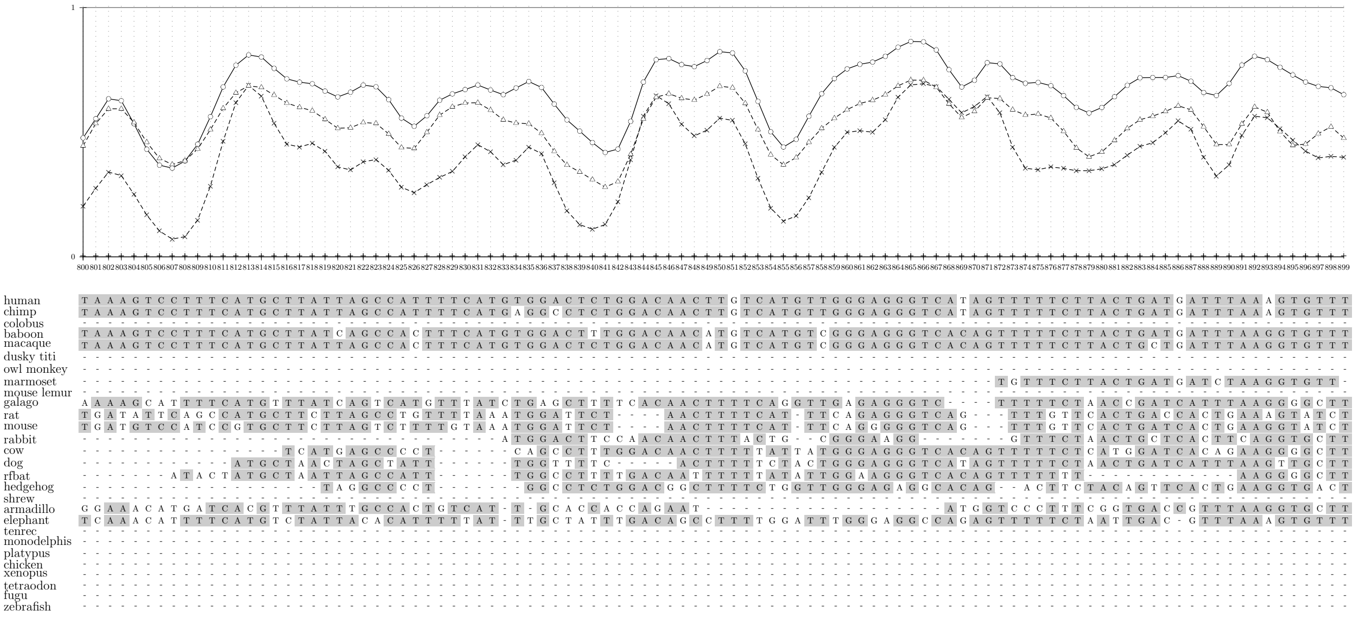
1
0
human
chimp
colobus
baboon
macaque
dusky titi
owl monkey
marmoset
mouse lemur
galago
rat
mouse
rabbit
cow
dog
rfbat
hedgehog
shrew
armadillo
elephant
tenrec
monodelphis
platypus
chicken
xenopus
tetraodon
fugu
zebrafish

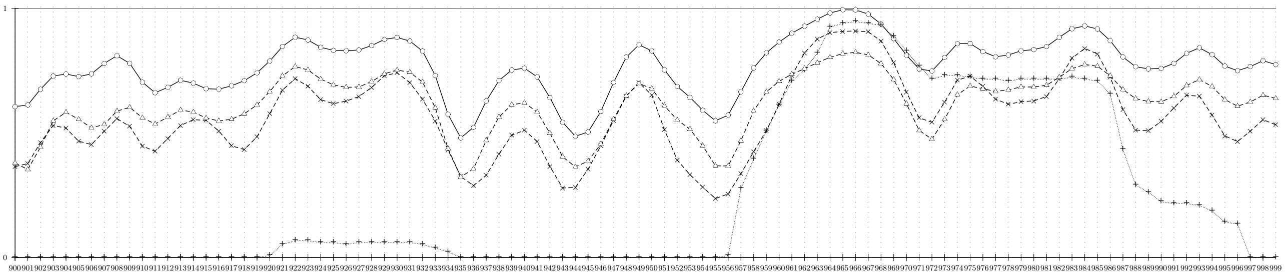

```
human        CTGTATTAAAGGCAGTGATCTTTTTAGCAAGTTTTAAAATATCAATAAGATGCTTGCAAAAGGCAGAATATGGAACATGACACAGAACAACATTTTGCTA
chimp        CTGTATTAAAGGCAGTGATCTTTTTAGCAAGTTTTAAAATATCAATAAGATGCTTGCAAAAGGCAGAATATGGAACATGACACAAAACAACATTTTGCTA
colobus      ----------------------------------------------------------------------------------------------------
baboon       CTGTGTTAAAGGCAGTGATCCTTTTAGCAAGTTTTCAAATATGGATGAGATGCTTGCAAAAGGCAGAATATGGAACATGACACAAAACAACCTTTTGCTA
macaque      CTGTGTTAAAGGCAGTGATCCTTTTAGCAAGTTTTCAAATATGGATGAGATGCTTGCAAAAGGCAGAATATGGAACATGACACAAAACAACCTTTTGCTA
dusky titi   ----------------------------------------------------------------------------------------------------
owl monkey   ----------------------------------------------------------------------------------------------------
marmoset     ------------------TCCTTTCAGCAAGTTTTACAATATTGCTCAGATGCTCACAAAAGGCAGAATATGGAACATGACACAAAACAACATTTTGCTA
mouse lemur  ----------------------------------------------------------------------------------------------------
galago       CTGTAGTA-----AGTGATCCTTTTAGCAAGTCTTCAAATTTCTGTAAGATTTTTGTAAGAGGCAGAATATGGAACATGACACAAAATAGTATTCTATTA
rat          GCATATAAAATCC-ATGATCCTTTTACCAAGTTTAAAAATGTCTCTTAGATAACTGAAAAGGGCAGAATATGCAACATGACTC-AAATAACACTTGGGCA
mouse        --ATATACAATTC-GTGATCCTTTTACCAAGTTT-TAAATGTTTCTTAGATATCTAAAAAAGGCAGAATATGCAACATGACTC-AAATAACACTTGGGCA
rabbit       CTGTATTCAAGTCAGTGGCCCTTTCAGCAAATTT-TAAGTTTCTATGA------------------AATATGGA----------AAATGACATTTTGCTA
cow          CTGTGTCAAAGACAGCGATCCTTTCAGCAAGTTTTCAGATTTCCGCAAGATGCTTATGTAAGGCAGAATATGGAAGATGACACAAAATGACATTTTGCTA
dog          TTGTATTAAAGACAGCAATTCCTTCAGCCAGTTCTAAAATTTCTACCAGATGCTTGTAAAAGGCAGAATATGTAAGGTAATTCAAAATAATGTTTTGCTA
rfbat        GGGTATTAAAAACAGTGATCCTTTCAGCAAGTTTTTAAATTTCTATAATATGCTTGTAAAAGACAGAATGTACAAGATAACACAAAACAACATTTCGCTA
hedgehog     TGTCATTAAAGAC------TTTTTCAATGAGTTTTAAATTTTC---------------------------------------------------------
shrew        ----------------------------------------------------------------------------------------------------
armadillo    CTGTATTAAAGGTGATTCCCCTTTCAGCAAGCTTTGCGGTTTCTCTAAGATGCTTGG----------------------ACACAAAATAACATTTTACTA
elephant     TTGTACTAAGGACAGGGATCCTTTCAGCAAGTTTTCAAATCTCTATAAAATGCTCAGAAAAGGCAGAATAGGCAACACGACACAA---AACATTTTGCTA
tenrec       ----------------------------------------------------------------------------------------------------
monodelphis  ----------------------------------------------------------------------------------------------------
platypus     ----------------------------------------------------------------------------------------------------
chicken      ----------------------------------------------------------------------------------------------------
xenopus      ----------------------------------------------------------------------------------------------------
tetraodon    ----------------------------------------------------------------------------------------------------
fugu         ----------------------------------------------------------------------------------------------------
zebrafish    ----------------------------------------------------------------------------------------------------
```

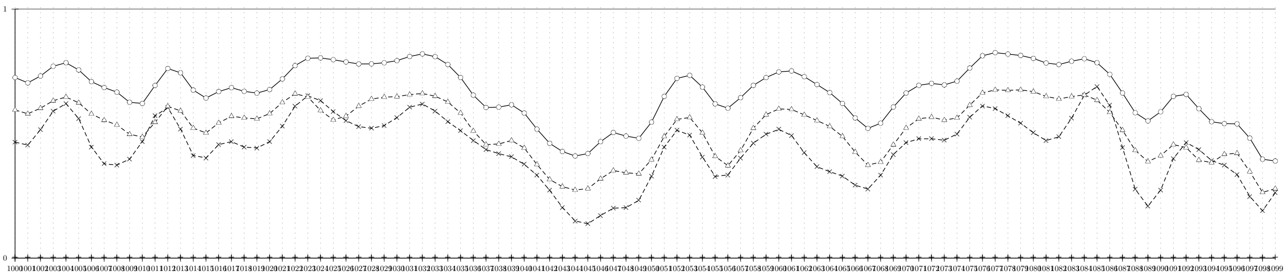

```
human        GATATGTGAATGAAGTATTATTTATAATTTACAGCTCACAATGATTACTTTGGGTTGCTGTGAAATGTTGACCAGCAGCTTTTCTGCTGCTGTAATAAAA
chimp        GATATGTGAATGAAGTATTATTTATAATTTACAGCTCACAATGATTACTTTGGGTTGCTGTGAAATGTTGACCAGCAGCTTTTCTGCTGCTGTAATAAAA
colobus      ----------------------------------------------------------------------------------------------------
baboon       GCTATGTGAATGAAGTATT---TATAATTGACAGCCCATGATGATCACTGTGGGTTGCTGTGAAATGCTGACCATCAGCTTTTCTGCTGCTGTAATAAAA
macaque      GCTATGTGAATGAAGTATTATTTATAATTGACAGCCCACGATGATCACTTTGGGTTGCTGTGAAATGTTGACCATCAGCTTTTCTGCTGCTGTAATAAAA
dusky titi   ----------------------------------------------------------------------------------------------------
owl monkey   ----------------------------------------------------------------------------------------------------
marmoset     AATATGTGAATGAAGCATTATTTATAATTTACAGCTCACAATTATTACTTCGGGTTGCTGTGAACTGTGGACCAGCAGCTTTGCTGCTGCTGTAAC-ACA
mouse lemur  ----------------------------------------------------------------------------------------------------
galago       GATGTGTAAGTTAACTATTATTCATAACT-ACACCTCACACTTACTACACCAAGTTACTGTGAAATATTGACCAATAACTTTCCTGCAACGCTAATAGAG
rat          TGTCTGT----AAAGTATCACTTGTAAGTTACAGTCCGAAATACTGCAGCTGAGTCCCCATGGACTGCAGGCTGACAGCTTTTCTCT--CAGTAGCAAAA
mouse        GGTTTGTAAAGAAAGTATCATTTGCAATTTACAGTCCAAACTGCTGCAGCTGAGGTCATA------------TGACAGCTTTTCCCTTATAGTCGCAAAG
rabbit       GGTGTGACAAGGAAGAAGTATTTGTAACTTACAGCTCCCAGTCACCCCACTGGGCAACTGCGCAACGTCAACCAGC-GCTTTTATCG--CTGTTGC----
cow          GGTGTGTACGGGAAGAA-GGGCTGTCATTTGCAGCTCGCAGTCACCCCAGTGGCTTCCTGTGGAATGGGGGGTAGCAGGTCTTCTCCTCTTGTAAGAA-G
dog          AGTGTGTAAATGAAAAA-CAGCTGTAATTTACAGCTTTCAATTAATCCAA-GGCTTCCCGTGAAATAGTG---AGCAA-----CTCCTGGTGTGATAATA
rfbat        GGTGTGCAAATGAAGGA-CAGTTGTCATTTATAGCTCACAATGACTCCCATGGCTTCCTATGAAAGGGTGACCAGCAGCTTTTCTCCTGCTGTGAGAG-A
hedgehog     ----------------------------------------------------------------------------------------------------
shrew        ----------------------------------------------------------------------------------------------------
armadillo    GATGTACAAACGAGA---TAATTGTAATTCAGAGCTCACAACTGTTACATAAGCTTACTGT-------TGAC---CAGCTTTTCTCCTGTAATGACAGGC
elephant     GATGTATAAAAGAAGTATTATTTGTAATTTACACCTCGCAATTATCGTACTGGCTTTCTGGGAGATGCTGGCCAACAGCCTGTCTCCTGCTGT-ATAACA
tenrec       ----------------------------------------------------------------------------------------------------
monodelphis  ----------------------------------------------------------------------------------------------------
platypus     ----------------------------------------------------------------------------------------------------
chicken      ----------------------------------------------------------------------------------------------------
xenopus      ----------------------------------------------------------------------------------------------------
tetraodon    ----------------------------------------------------------------------------------------------------
fugu         ----------------------------------------------------------------------------------------------------
zebrafish    ----------------------------------------------------------------------------------------------------
```

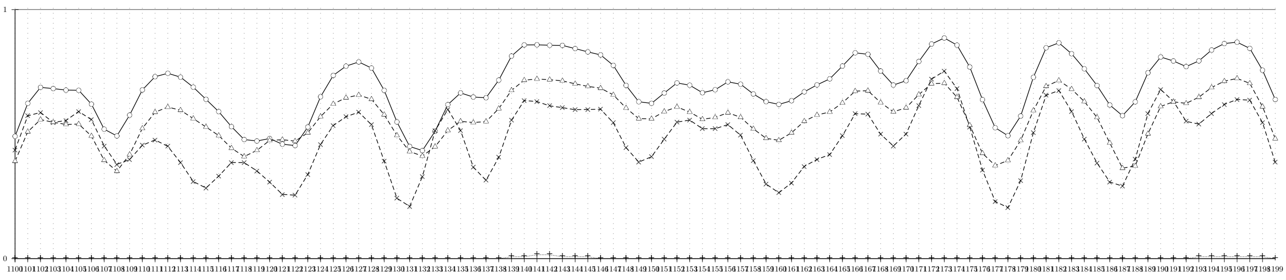

```
human        CAGCAGCCATGTCTGGACATCACTGGCTGCTTCAGCTATTCTTTAACCTTCTAGTCCCAGGATATGCTCATCAACAAGAACAAGTTTCTGCTTAGGGAAG
chimp        CAGCAGCCATGTCTGGACATCACTGGCTGCTTCAGCTATTCTTTAACCTTCTAGCCCCAGGATATGCTCATCAACAAGAACAAGTTTCTGCTTAGGGAAG
colobus      ----------------------------------------------------------------------------------------------------
baboon       CAGCAGCCACGTCTGGACATCACTGGCTGCTTCAGCTATTCCTTAACCTTCTAGTCTCGGGATATGCTCATCAACAAGAACAAATTTCTGCTTAAGGAAG
macaque      CAGCAGCCACGTCTGGACGTCACTGGCTGCTTCAGCTATTCCTTAACCTTCTAGTCTCGGGGTATGCTCATCAACAAGAACAAGTTTCTGCTTAAGGAAG
dusky titi   ----------------------------------------------------------------------------------------------------
owl monkey   ----------------------------------------------------------------------------------------------------
marmoset     CAGCGGCCATGTCTGGACATCACCGGCTGCTTCAGCTATTCTTTAACCTGCCAGTCCCAGCATATGCTCATCAACAAGAACAAGTTTCTGCTTAAGGAAG
mouse lemur  ----------------------------------------------------------------------------------------------------
galago       ----------GCCTGAACACCAGGGGTTGCTCTCCTTACTCTTTAGCCTTCTAGTC-CGGGATTTGCTTATCACCAAGAACAAATTTCAGCTT----AAA
rat          GGATCAGGCTGCTTGA------------GCTCTAGCTCTTCCTTAACCCCGCAGCCCCAAAACTAACTCCCCACCAATCCAAAGTGTAAGCCT----AAG
mouse        CGGCCAGGCTGCTTGA------------GTTCCCGCTCTTCTTTAACCTTCCAGCCCC-AAACTAACTCCCCACCAATGCCAAGTTTAAGCCTAAGTAAG
rabbit       ----TGGGATG--GAA------------GCAGCCGCCAGTCTTCAGCCTCCCGGGCCCAGGCTTTGCTCACCACCCCGAGCAAGCCTGGGCGTAAGAAAG
cow          TGGCAGCTGTAAC---------------------------CTT--------------CAGGAT-CGCTCACCACCA---CCAAGTTCTTGCTTAAGAAAC
dog          TGGAAGC-----------------------------------------------------------------------------------CTTAAGAAA-
rfbat        AGGCAGCCCTGCCTCAGCCTAGGTGGCCG-----------CTT--------------CAGGAT-TGCTTGTCACCA---CCAAGTTTCTGCTGAAGAAAT
hedgehog     ----------------------------------------------------------------------------------------------------
shrew        ----------------------------------------------------------------------------------------------------
armadillo    TGGCAGCTTGGCCTGAACTTAAGTGACTGCTTTAGCTCTTCTTTAACTTTCTAGTTCACAGGT------------------AAGTTTCTGCTCAAGA--A
elephant     TGGCAGCCTTGCCTGCCCGTACACGGCTGTCCTAGCAATTCTT-AACCTTCTAGCCCCAGAATTTCCTCACTACCACAGGCAAGTTTTGGCTTCAGAAAC
tenrec       ----------------------------------------------------------------------------------------------------
monodelphis  -------------------------------------------------------------------------------------------------AAG
platypus     ----------------------------------------------------------------------------------------------------
chicken      ----------------------------------------------------------------------------------------------------
xenopus      ----------------------------------------------------------------------------------------------------
tetraodon    ----------------------------------------------------------------------------------------------------
fugu         ----------------------------------------------------------------------------------------------------
zebrafish    ----------------------------------------------------------------------------------------------------
```

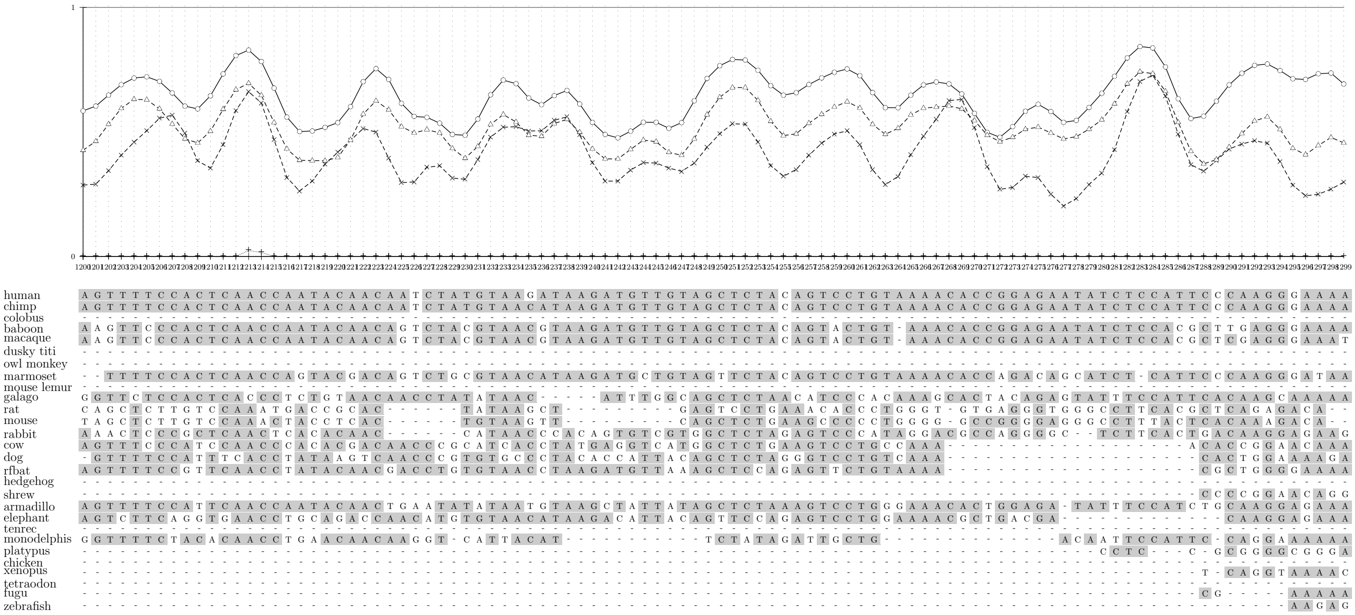
1
0
human
chimp
colobus
baboon
macaque
dusky titi
owl monkey
marmoset
mouse lemur
galago
rat
mouse
rabbit
cow
dog
rfbat
hedgehog
shrew
armadillo
elephant
tenrec
monodelphis
platypus
chicken
xenopus
tetraodon
fugu
zebrafish

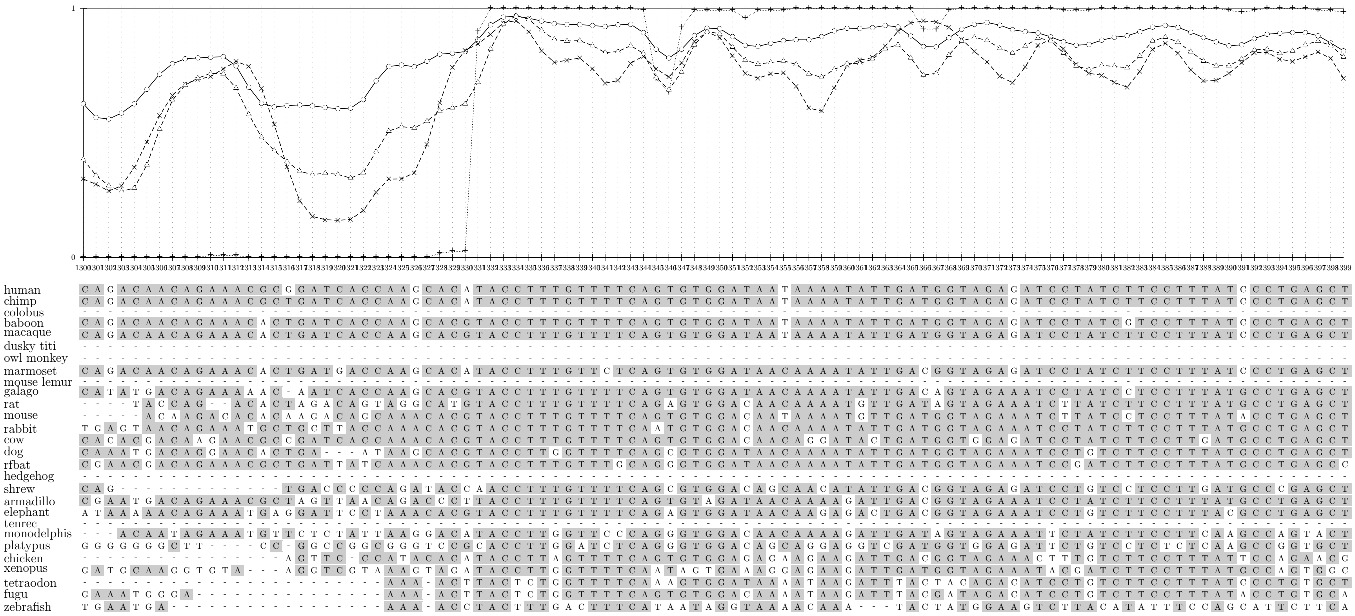
1
0
human
chimp
colobus
baboon
macaque
dusky titi
owl monkey
marmoset
mouse lemur
galago
rat
mouse
rabbit
cow
dog
rfbat
hedgehog
shrew
armadillo
elephant
tenrec
monodelphis
platypus
chicken
xenopus
tetraodon
fugu
zebrafish

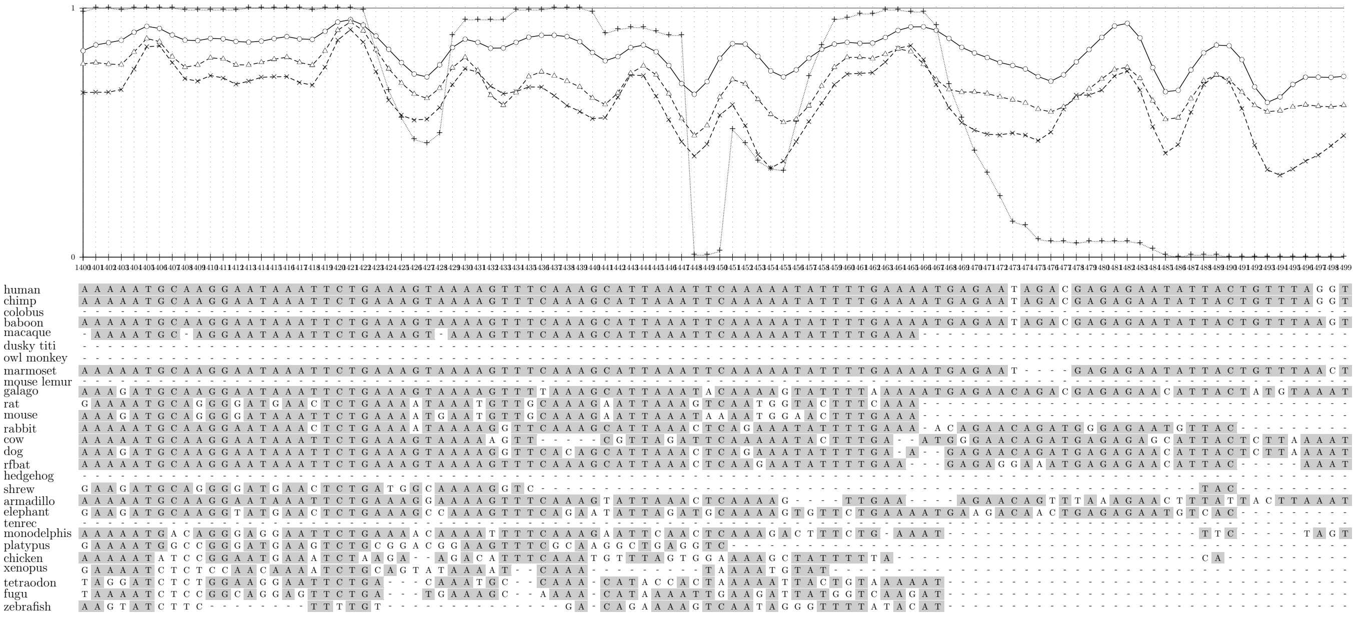
1
0
human
chimp
colobus
baboon
macaque
dusky titi
owl monkey
marmoset
mouse lemur
galago
rat
mouse
rabbit
cow
dog
rfbat
hedgehog
shrew
armadillo
elephant
tenrec
monodelphis
platypus
chicken
xenopus
tetraodon
fugu
zebrafish

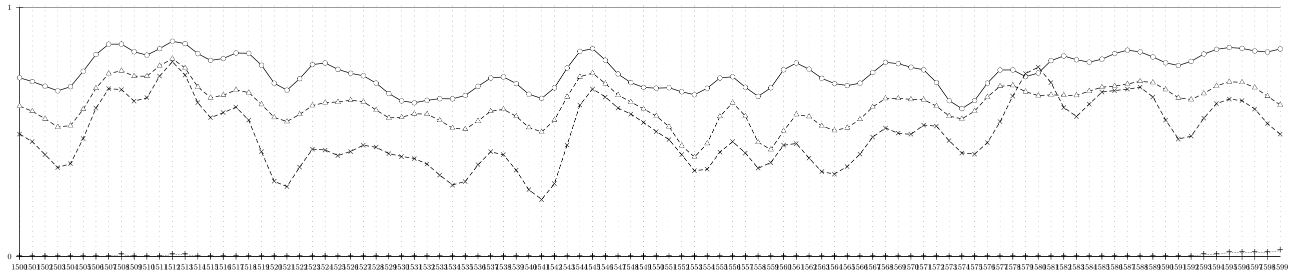

```
human        GGTACAGCAAACAATGCCATTTACTTTTTCAAGTTTTTTTTTTTTTTAACTATACAAGTTTCCGGTAGAAATAACCACTTCCATGACATAAGAAATCAAGAA
chimp        GGTACAGCAAACAATGCCATTTACTTTTTCAAGTTTTTTTTTTTTTTAA-TATACAAGTTTCCGGTAGAAATAACCACTTCCATGACATAAGAAATCAAGAA
colobus      ----------------------------------------------------------------------------------------------------
baboon       AGTACAGCAAACAATGCCTTTTACCTTTTCAAGTTTTTTTTTTTTTTAACTACACAAGTTTCCAGTAGACATAACCACTTCCATGACATAAGAAATCAAGAA
macaque      ----------------------------------------------------------------------------------------------------
dusky titi   ----------------------------------------------------------------------------------------------------
owl monkey   ----------------------------------------------------------------------------------------------------
marmoset     GTTACAGCAAACAATGCTATTCACCTTTTTTAG--TTGTTTTTTTAACTATACCAGTTCTCAGTAGGAATAACCACTTCCGTGACATAAGAAATCAAGAA
mouse lemur  ----------------------------------------------------------------------------------------------------
galago       GGTACAACAAACAACTCCACTTGCCTTTTTAAACTTTCTTTTTTTAGCTATACAAATTTGCAGTAG----------------GACATAAGAAATCAAGAA
rat          TGTAGAGCAAATAACGCCAC------CCCG----ATTTCTGCACCAATTATACAAGTTTTCAACAGTAA--ACCGGCTCCATGGCACAGGGAGTCAAGG-
mouse        TGTAGAGCAAATAATGCCAC-----ACCTG----ATTTCTGTGCCAGGTTCACAG--TTTCAACAGTAA--ACTGGTTCCGTGGCACAGGGACTCAAGA-
rabbit       TATTTAGCAAGTAATGT---------CTTT----TTTTCAGTTTTAATTATACAA--GTTCA--------------------------------------
cow          GGTACGGCAAACAATGCCATTGACTTTTTTAGGTTTGTCTTTTTTAATTACACAAGTTCACAGCAGGAAAAACTATTTTTGTGACATAGGAGATAAA---
dog          GGCGCAGCACACAATGCCATTTTCTTCTTTAGGTTTGTCTTTCTTAATTATGCAAGTTTACAGTAGGAATAACTACTT------CATAGGAAATCAG---
rfbat        GGTATAGCAAACAATGCCATCTACTTTTTCAAGTTTGTCTTTTTTAATTATACAAGTT--CAGTGGAAATGACCACTTTTGTGACAAAGGAAATCAA-A-
hedgehog     ----------------------------------------------------------------------------------------------------
shrew        ----------------------------------CGTTTACTCTAATTTTATTTTTACACAGTTGGAATGATT---------------------------
armadillo    GGTACAGCAAACAATGGCATTTACTTTTTTAAGTTTTTTGCTTTA-CTATATTAATTCTCACTTA------------------AC---------------
elephant     -----AGCAATCAATGGC--ATACTTTTTA----AAGTCTTTTTTTAATTATACGAATTCACA------------TTTTTTTTTAT--------------
tenrec       ----------------------------------------------------------------------------------------------------
monodelphis  GATGAATTAGACAAAAGCATTCA---------------TTCTTT-ATAAATTAATTCGCT---------------TTCTTGT------------------
platypus     ----------------------------------------------------------------------------------------------------
chicken      -CTC------TCCATGCCATTGACTTCTCT------------------CCCAAAAAACTGCAATAA----------------GAGTTAAAGGATG--A-A
xenopus      ----------------------------------------------------------------------------------------------------
tetraodon    ----------------------------------------------------------------------------------------------------
fugu         ----------------------------------------------------------------------------------------------------
zebrafish    ----------------------------------------------------------------------------------------------------
```

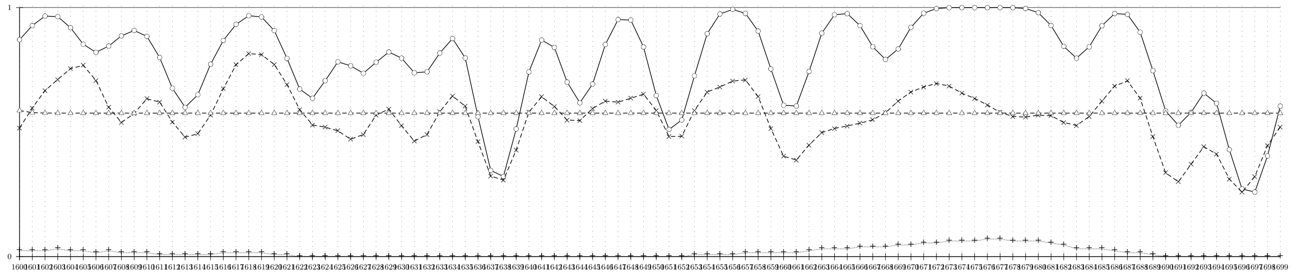

```
human        GCCAGGCAGAGTGGCTCATGCCTGTAAGCCCAGCACTACAGGAGGCCAAGGTGGGAGGATCCCTTGAGGCCAGGAGTTCAAGACCAGCCTGGGCAATATA
chimp        GCCAGGCAGAGTGGCTCATGCCTGTAAGCCCAGCACTACAGGAGGCCAAGGTGGGAGGATCCCTTGAGGCCAGGAGTTCAAGACCAGCCTGGGCAATATA
colobus      ----------------------------------------------------------------------------------------------------
baboon       GCCAGGCACAGTGGCTCATGCCTGTAAACCCAGCACTATGGGAGGCCAAGGCGGGAGGATCCCTTGAGGCCAGGAGTTCAAGAACAGCCTGGGCAATATA
macaque      ----------------------------------------------------------------------------------------------------
dusky titi   ----------------------------------------------------------------------------------------------------
owl monkey   ----------------------------------------------------------------------------------------------------
marmoset     GCCAGGC--AGTGTCTCATGCCTGTAAGCCCAGCACTTTGGGAGTCCAAGGTGGGAGGATTCCTTGAGGCC-----TTCAAGACCAGCCTTGGCAGTGTA
mouse lemur  ----------------------------------------------------------------------------------------------------
galago       ACCAGGCACAGTGACTCATGCCTATAATCCTAACACTCTGGGAGACCAAGGCAGGAGGATCACTTGAGCCCAGGAGTTCAAGACCAGCCTGAACAAGT--
rat          ----------------------------------------------------------------------------------------------------
mouse        ----------------------------------------------------------------------------------------------------
rabbit       ----------------------------------------------------------------------------------------------------
cow          ----------------------------------------------------------------------------------------------------
dog          ----------------------------------------------------------------------------------------------------
rfbat        ----------------------------------------------------------------------------------------------------
hedgehog     ----------------------------------------------------------------------------------------------------
shrew        ----------------------------------------------------------------------------------------------------
armadillo    ----------------------------------------------------------------------------------------------------
elephant     ----------------------------------------------------------------------------------------------------
tenrec       ----------------------------------------------------------------------------------------------------
monodelphis  ----------------------------------------------------------------------------------------------------
platypus     ----------------------------------------------------------------------------------------------------
chicken      ACCAGTGACAGCAAGTAATGCTGCCAAACC----------------------------------------------------------------------
xenopus      ----------------------------------------------------------------------------------------------------
tetraodon    ----------------------------------------------------------------------------------------------------
fugu         ----------------------------------------------------------------------------------------------------
zebrafish    ----------------------------------------------------------------------------------------------------
```

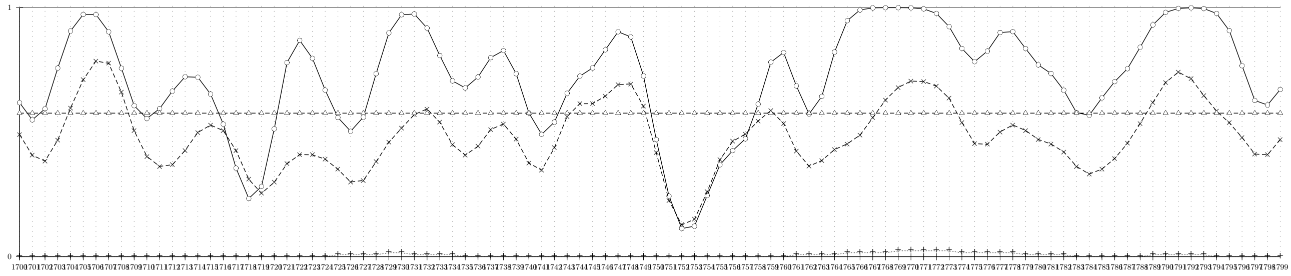

```
human        GCAAGACCCTGTCTCCAAAAAAAAATTTAAAAATTAGCTGGGTATGGTGGTGCGCACCTGTAGTCTTACTGTAGTCTTAGGTACCTAAGAGGCTGAGGTG
chimp        GCAAGACCCTGTCTCTAAAAAAAAATTTAAAAATTAGCTGGGTATGGTGGTGCACACCTGTAGTCTTACTGTAGTCTTAGGTACCTAAGAGGCTGAGGTG
colobus      ----------------------------------------------------------------------------------------------------
baboon       GCAAGACCCTGTCTCTAAAAAAAAATTTAAAAATGATCTGGGTATGGTGGTGTGCACATGTACTCTTACTGTAGTCTTAGCTACCTAGGAGGCTGAGGTG
macaque      ----------------------------------------------------------------------------------------------------
dusky titi   ----------------------------------------------------------------------------------------------------
owl monkey   ----------------------------------------------------------------------------------------------------
marmoset     GTAAGACCCCATCTCT-ATGAAAAGTTTAAAAATTAGCTGTTTATGGTGGTGCACACTTGTAGTCTTA---TAGTCTTAGCTACCTAGGAGGCTGAGGTG
mouse lemur  ----------------------------------------------------------------------------------------------------
galago       GTGAGACCCTGTTTCT-ACTAAAAATAGAAAAATTAGCTGGGCATTGTGGCCGGTGCCTG-----------TAGTTTTAGCTAGTT-GGAGGCTGAGCAG
rat          ----------------------------------------------------------------------------------------------------
mouse        ----------------------------------------------------------------------------------------------------
rabbit       ----------------------------------------------------------------------------------------------------
cow          ----------------------------------------------------------------------------------------------------
dog          ----------------------------------------------------------------------------------------------------
rfbat        ----------------------------------------------------------------------------------------------------
hedgehog     ----------------------------------------------------------------------------------------------------
shrew        ----------------------------------------------------------------------------------------------------
armadillo    ----------------------------------------------------------------------------------------------------
elephant     ----------------------------------------------------------------------------------------------------
tenrec       ----------------------------------------------------------------------------------------------------
monodelphis  ----------------------------------------------------------------------------------------------------
platypus     ----------------------------------------------------------------------------------------------------
chicken      ----------------------------------------------------------------------------------------------------
xenopus      ----------------------------------------------------------------------------------------------------
tetraodon    ----------------------------------------------------------------------------------------------------
fugu         ----------------------------------------------------------------------------------------------------
zebrafish    ----------------------------------------------------------------------------------------------------
```

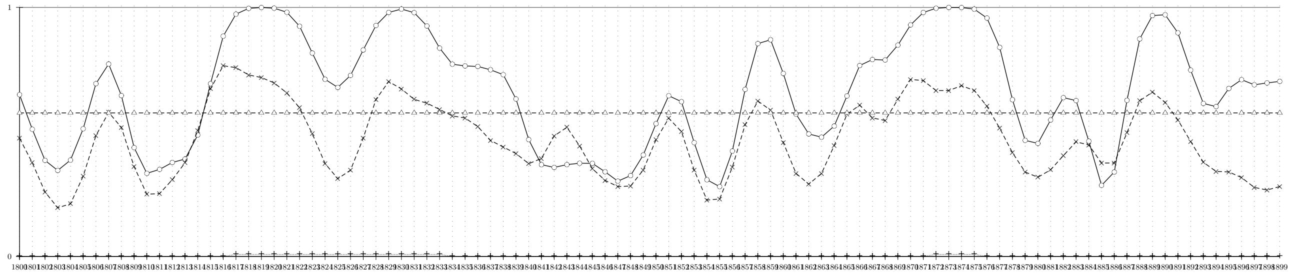

```
human        GCAGGATCACCAGTACCCAGGAGTTTGAGGTTGCAGTAAGCCATGATCGCACCACTGCACTCCAGCCTAGGCAACAGAGCAAGACGCTGTCTCCAAAAAA
chimp        GCAGGATCACCAGTACCCAGGAGTTTGAGGTTGCAGTAAGCCATGATCGCACCACTGCACTCCAGCCTAGGCAACAGAGCAAGACGCTGTCTCCAAAAAA
colobus      ----------------------------------------------------------------------------------------------------
baboon       GCAGGATCAGTAGTACCCAGGAGTTTGAGGTTGCAGTAAGCTATGATTGCACCACCACACTCCAACCTAGGCAACAGAGAAAGACAGTGTCTCCAAAAAA
macaque      ----------------------------------------------------------------------------------------------------
dusky titi   ----------------------------------------------------------------------------------------------------
owl monkey   ----------------------------------------------------------------------------------------------------
marmoset     GCAAGATCACTAGCACCCAGGAGTTTGAGGTTGCAGTAAGTCACGATCGCACCACTGCACTCCAACC-AGGC-ACAGAGCAAAACCCTGTCTCCAAACAA
mouse lemur  ----------------------------------------------------------------------------------------------------
galago       GAGAATTCACCGGAGCCCAGGAGTCTAAGGTTGCTGTGAGCTACACTAATGCCTTAGCACTGTAGCCTGGGCAACAGAGTGAGAC--TGTCTCAGAAA--
rat          ----------------------------------------------------------------------------------------------------
mouse        ----------------------------------------------------------------------------------------------------
rabbit       ----------------------------------------------------------------------------------------------------
cow          ----------------------------------------------------------------------------------------------------
dog          ----------------------------------------------------------------------------------------------------
rfbat        ----------------------------------------------------------------------------------------------------
hedgehog     ----------------------------------------------------------------------------------------------------
shrew        ----------------------------------------------------------------------------------------------------
armadillo    ----------------------------------------------------------------------------------------------------
elephant     ----------------------------------------------------------------------------------------------------
tenrec       ----------------------------------------------------------------------------------------------------
monodelphis  ----------------------------------------------------------------------------------------------------
platypus     ----------------------------------------------------------------------------------------------------
chicken      ----------------------------------------------------------------------------------------------------
xenopus      ----------------------------------------------------------------------------------------------------
tetraodon    ----------------------------------------------------------------------------------------------------
fugu         ----------------------------------------------------------------------------------------------------
zebrafish    ----------------------------------------------------------------------------------------------------
```

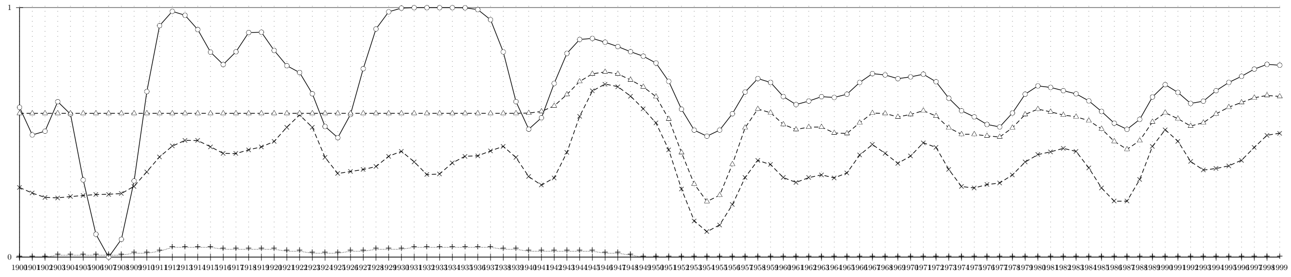

```
human        ACAAAACAAAACAAAACAAAAAGGAAGAAAGAAAAGAAATCAAGAGCACCTACAAGCGAACCAATGACAGAACTTTTTAAGAAGTTCAGTAGGTCTTTAG
chimp        AAAAAAAACAA--AAACAAAAAGGAAGAAAGAAAAGAAATCAAGAGCACCTACAAGCGAACCAATGACAGAACTTTTTAAGAAGCTCAGTAGGTCTTTAG
colobus      ----------------------------------------------------------------------------------------------------
baboon       ACAAAACAAAACAAAACAAAAAGGAGGAAAGAAAAGAAATCAAGAGCACCTAAAGGTGAACCAATGACAGAACTTTTTAAGAAGTTCAGTAGGTCTTTAG
macaque      ----------------------------------------------------------------------------------------------------
dusky titi   ----------------------------------------------------------------------------------------------------
owl monkey   ----------------------------------------------------------------------------------------------------
marmoset     AC-----------AAACAAAACGGAAGAAAG--AAGAAATGAAAAGCACCTAATGGTGAACCAGTGACAGAACTCTTAAAGAAGTTCAGTAGGTCTTTAG
mouse lemur  ----------------------------------------------------------------------------------------------------
galago       -------------AAAAAAAAAGGGGG------------------GTACCTAAAAGTG-CCTAATGACAGAACTTTTAAAGAAGTTTGGTA-CTTTTTAG
rat          ----------------------------------------------CATCTCAGGGTGAAATGACCACGGAA--------------------------AT
mouse        ----------------------------------------------CATCTCAAGGTAAAACAATCACAGAACTTTCAGGAAGGG-TGGTAGTTGCCCAG
rabbit       ----------------------------------------------TACTTAAGCATGAAGCAAAATCAATACTGACTGAAAG-----------------
cow          -----------------------------------------------ATCTAAAAGCAATACAATGAGTGAACCACTCAAGTT---CAACAGTTCTTTGG
dog          -----------------------------------------------ATCTAAAGGTAAAACAATAACAGAACCATTAAAGTTGTTCATTAGTTCTGT--
rfbat        -----------------------------------------------ATCTACAGCTAAAATAATGACAGGAAGATTAAAGTTGTTCCATAGCTCTTTAG
hedgehog     ----------------------------------------------------------------------------------------------------
shrew        ----------------------------------------------------------------------------------------------------
armadillo    --------------------------------------------AGTACATAAAGTAGAAACAATGACAGAATTTTTTAAAACGCTCAGTCATTCTTTAG
elephant     --------------------------------------------AGTACCTACGGCTAAAAAAATTACAACACTTTTTGAGACGTCCCATAGCCCTTTAG
tenrec       ----------------------------------------------------------------------------------------------------
monodelphis  --------------------------------------------AGTACCAAGGACTAAGAACC------------------------------------
platypus     ----------------------------------------------------------------------------------------------------
chicken      ----------------------------------------------------------------------------------------------------
xenopus      ----------------------------------------------------------------------------------------------------
tetraodon    ----------------------------------------------------------------------------------------------------
fugu         ----------------------------------------------------------------------------------------------------
zebrafish    ----------------------------------------------------------------------------------------------------
```

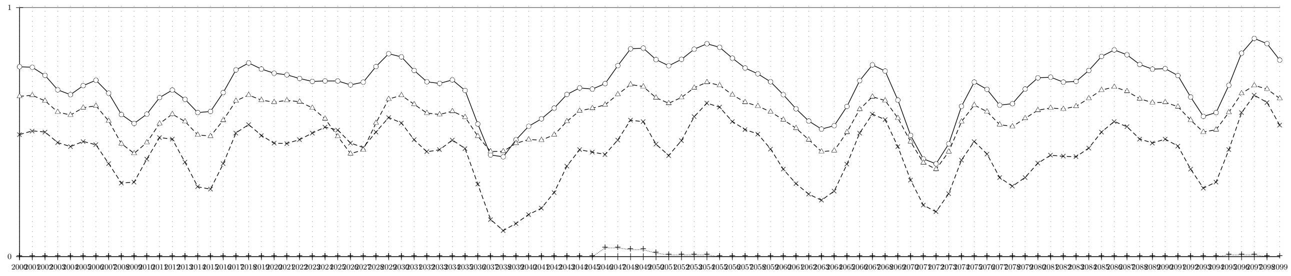

```
human        TTCCATTTTACTTTTCTTTTCTCATATTAAAATTAACTAAATACTATTTGAAAGAGAATGTACGGTATAACAAACTAATACAATTTCTCTCTTGCTTAGA
chimp        TTCCATTTTACTTTTCTTTTCTCATATTAAAATTAATTAAATGCTATTTGAAAGAGAATGTACGGTATAACAAACTAATACAATTTCTCTCTTGCTTAGA
colobus      ----------------------------------------------------------------------------------------------------
baboon       TTCCATTTTGCTTTTCTTTTCTCATATTAAAATTAACTAAATACTATTTGAAAGAGAATGTATGGTATAACAAACTAATAAAATTTCTCGC----TTAGA
macaque      -----------------------------------------------------------------------------------------------TTAGA
dusky titi   ----------------------------------------------------------------------------------------------------
owl monkey   ----------------------------------------------------------------------------------------------------
marmoset     CTCCATTGTACTT---TTTTCTCACATTGAAATTAATTAAATACTATTTGAAAGAGAATGTACAGCGTAACAGACTAGTAAAATTTCTCTCTTGCTTAGA
mouse lemur  ----------------------------------------------------------------------------------------------------
galago       TTCTGTTTTACTTTCTGTTTCTCACAGTAAAATTAACAGAACACTGTTTGCAAGAGAATGCATACTATAATAAACTAAGAGAATTTCTTCC----TTAGA
rat          CCCCACTTCCTGTTTCCTTACATGAAGTAAAGGCAACATGGAGCTG------GGAGGATA----------------------------------------
mouse        CCCCATTTAGCGTTTCCTTGCATGAAGTAACAGTCACCTGGTGCTATTTGGTGGGGGGTG----------------------------------------
rabbit       ---------------------------CAAAATCAA-----TACTGACTGAAAGAGAATGTGTGTTGTAACAGCTTTCTAAAG---CTCTATTGG-----
cow          TTCTATCTTCCCTTTCTTTTTTCACATTAAAGTTAA-----TACTATTTGAGAGAGCACATATGGTATTA--GTTTTCCAAAGTTTCACTCTTCATTAGA
dog          ---TATTTCACTTTCCTTTTCTCACATTAAAATAAA-----AAATATCTGAAAAAGAATGTATGGTATTATGATTTTGCTAAATGTCTCTCTTTGTTAGA
rfbat        TTCTGTTTTAGTTTCCATTTCTCACATAAAAATTAA-----TGCTGTTTGAAAGAGAATGTATGTTATTATAGCTTTCCAAAGTTT--CTCTTTGTTAGA
hedgehog     ----------------------------------------------------------------------------------------------------
shrew        ----------------------------------------------------------------------------------------------------
armadillo    TTCCATTTTACTTTACTTTCCTTACATGAAAATTAATTATATATTGTTTGAAAGAGAATGTGTAGTATTCAGTTTTTCTAAAACTCCTCTCTCTGATATA
elephant     TTCTGTTTTACTCCCATTTTCTTATAT----------------------------GATTGAGTGTTATTCCACTTTTCTAAAATTTCTCTCTGTA-----
tenrec       ----------------------------------------------------------------------------------------------------
monodelphis  ----------------------------------------------------------------------------------------------------
platypus     ----------------------------------------------------------------------------------------------------
chicken      ----------------------------------------------------------------------------------------------------
xenopus      ----------------------------------------------------------------------------------------------------
tetraodon    ----------------------------------------------------------------------------------------------------
fugu         ----------------------------------------------------------------------------------------------------
zebrafish    ----------------------------------------------------------------------------------------------------
```

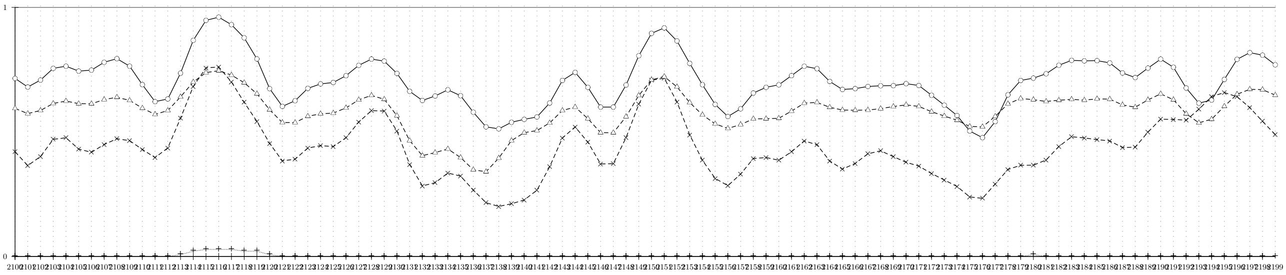

human	CTTGCTGAGATGTGTTTAAAGAGATGGTCATGATTTTTTAAGTTGGCAGTAGAATTTTGGGTGATTTAGTTTGTTTACTTTTTGCTTTCTCTTCCTACTT
chimp	CTTGCTGAGATGTGTTTAAAGAGATGGTCATGATTTTTTAAGTTGGCAGTAGAATTTTGGGTGATTTAGTTTGTTTACTTTTTGCTTTCTCTTCCTACTT
colobus	--
baboon	CTTGCTGAGATATGTTTAAAGAGAAGGTCATCATTTTTTAAGTTGGCAGTAGAATTCTGGTTGATTTAGTTTGTTGACTTTTTGCTTTCTCTTCCTACTT
macaque	CTTGCTGAGATATGTTTAAAGAGAAGGTCATCATTTTTTAAGTTGGCAGTAGAATTTTGGTTGATTTAGTTTGTTGACTTTTTGCTTTCTCTTCCTACTT
dusky titi	--
owl monkey	--
marmoset	CTTGCTGAGATGTGTTTAAAGAGATGATCATCATTTTACAGGCTGACATTAGAATGTTGGGTAATTTAGTTTATTTACTTTCTGTTTTCTCTTCCTACTT
mouse lemur	--
galago	CTTGCTCAGA----------GGTATGGTCATATTTTT---GGCTGGTGGTAGATTTTTGGGTGACTTTGCTTGCTTTTTCTTTGCTTTTTCTTCGTATTT
rat	--
mouse	--
rabbit	----------------TATAGAG---------TTTGCTCATGGTAGGAGC-GACTTCTGTGTGACTTGGTGTGCTC-----TGGCTTGCTCTGTGTACCT
cow	CCAGTTGAGATGTATTTAAAATGACAGTTGTCACTTTATTGACTGGCCGTAGAATT-----GGA-----------------CTGATTTATTTGC-----T
dog	ATTGCTGAGTTACATTTAAA-TGATGGTCATCATTTTATTGACTGGTGGTAGAATTCAGAATGA-----------------TTGCTTTCTTTTCAAACTT
rfbat	CTTGCTAAGATGTATTTAAAACGATGGTCATCATTTTGTTGGATGGTGGTAGAATTTGGGGTGATAT-GCTTGT-------TTGCTTTCTTTTCATACTT
hedgehog	--
shrew	--
armadillo	TTAGCCGAAATATGTTTAAAATAATGTTCATCAA-GTATTGACTGGTGGTAGAAGTTGGGGT------GCTTATTTGCTTTTTGCATTTTCTTTGTACTT
elephant	--
tenrec	--
monodelphis	--
platypus	--
chicken	--
xenopus	--
tetraodon	--
fugu	--
zebrafish	--

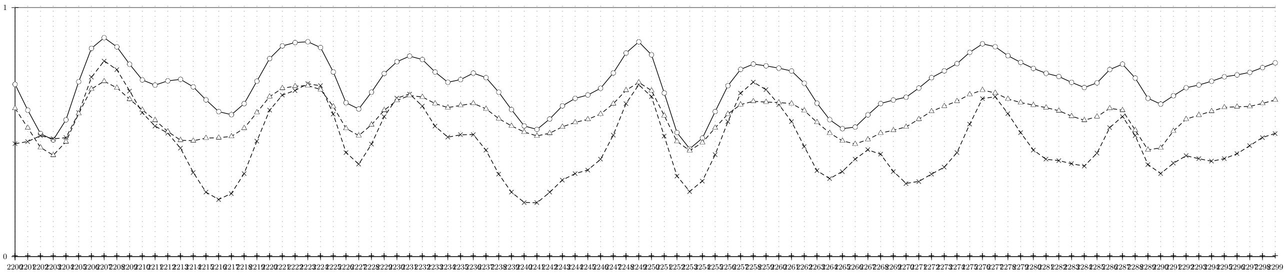

human	TTCAATCCCCAAGGAAATGGCCACCAGAGCTTGACACTGTTATCATTATTAATGCAGTAGTGACAGTGGGCAGAAGCAGCAAAACAATTACAAATTTCCT
chimp	TTCAATCCCCAAGGAAATGGCCACCAGAGCTTGACACTGT-ATCATTATTAATGCAGTGGTGACAGTGGGCAGAAGCAGCAAAACAATTACAAATTTCCT
colobus	--
baboon	TTCAATCCCCAGGGAAATGGCCACCAGCGCTTGACACT---ATCATTATTAATGCAGTGGTGACAGTGGGCAGAAGCAGCAAAGCAATTACAATTTTCCT
macaque	TTCAATCCCCAGGGAAATAGCCACCAGCGCTTGACACT---ATCATTATTAATGCAGTGGTGACAGTGGGCAGAAGCAGCAAAGCAATTACAATTTTCCT
dusky titi	--
owl monkey	--
marmoset	TTCAATCCCCAGGGAAACAGCCACCAGGGCTTGACACT---ATC---ATTAACACAGTG------GTGGGCAGAAGCAGCAAAGCAATTACGAGTTTCCT
mouse lemur	--
galago	TTTGATCCCCAGGGGAACAGCCACCAAAGCTTGACACTGG-ATCATAACTAATACGCTGGT-ACAGAGAGTAGA---ATCAAAGCAATTCTAATTCTAC-
rat	--
mouse	--
rabbit	TCCGACCCTGGGGGAGACGGCCACCAGAAGGCGGTGCTGT-GTCCCT-CTAGCACA-CGGCGGCCCCAGACTGAAGCATCTGAGATGTTCCAGTGTTACT
cow	TTATCTCCCCAAGGACTCAGTCTCCATAGCTTGATGCTCT-GGCATTACTAACATACTGGTGACACCGGTAAAAAGCAACAAACCCATTACAATTTCTCT
dog	CTGGATCCCCAAGGAATCAGCCACCACAGCTTGATGCCAT-ACCATTGCTAATGTACTGATGATACAGGGCAGAAACATCAGAGCAATTATAATTTCACC
rfbat	CTAGGTCCCCAGGTAATCAGCCACCACAGCTTGATTCTGT-ACCCTCACTAACAGACTGGTGATGCCAGGCA-CGGCATCAAAGCAATTACAATTTCACA
hedgehog	--
shrew	--
armadillo	TTCTGTCCCCAAGTAAGTAACCATC--CGCTTGCTGC----TTTATTACTAATGTGCTGGC-------------------------ATAATAATTTTACT
elephant	--
tenrec	--
monodelphis	--
platypus	--
chicken	--
xenopus	--
tetraodon	--
fugu	--
zebrafish	--

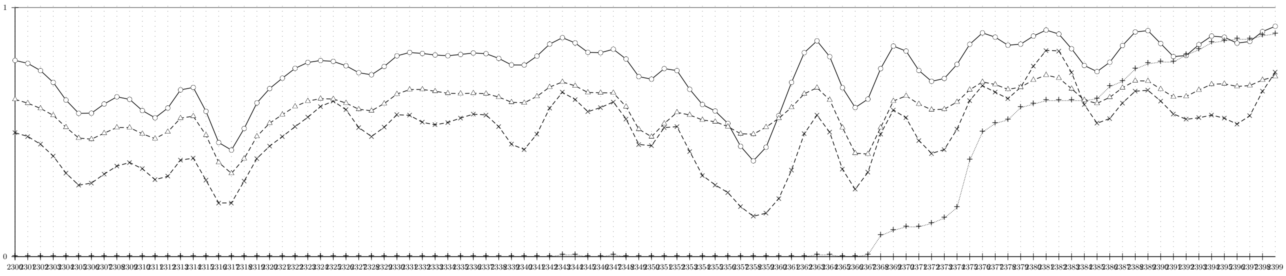

human	TGCTCCACACATGCTTACTGGGCACACAGAATTTCTACAAAAGCAAATTCATTATTTGTGAAAAAGGAAAAATCAGAAAATACCAGACAAACCAAAAGAA
chimp	TGCTCCACACATGCTTACTGAGCACACAGAATTTCTACAAAAGCAAATTCATTATTTGTGAAAAAGGAAAAATCAGAAAATACCAGACAAACCAAAAGAA
colobus	--
baboon	TGCTCCACACAGGCTTACTGGGCACACAGAATTTCTACAAAAGCAAATTCATTATTTGTGAAAAAGGAAAACTCAGAAAATACCAGACAAACCAAAAGAA
macaque	TGCTCCACACAGGCTTACTGGGCACACAGAATTTCTACAAAAGCAAATTCATTATTTGTGAAAAAGGAAAAATCAGAAAATACCAGACAAACCAAAAGAA
dusky titi	--
owl monkey	--
marmoset	TGCTCCACACACGCTTACTGAGCACACAGAATCTCTACAAAAGCACATTCATTATTTGCAAAAAAGGCAAAATCAGAAAATACCAGACAAACCAAAGGAA
mouse lemur	--
galago	TGCTGCACTCATGCTTGT--GGCACACACAATTTCTACACAAGCAAATTGCTTAC--------AAAGAAAAATCAGAATACACCAAACAAACCCAAAGAA
rat	--
mouse	--
rabbit	TGGTCTGCACATCTTTAGAGGCTTCACATAATTTCTACAAAAACAAATTTCTTATTCTTAGAAAAAGTAAA-TCAGAAAATACCA----AAGCAAAAGAA
cow	TGCTCTATACATTCTTCACGAGCACATATAATTTCTTCAAAGGCAAATTCATTATTCAAAAGAAAAAAAAAATCAGAAAATACCGGATA-----------
dog	TGCTCCAGAGGTGCTCCATGGGCACACAT----TCTACAAAAGCAAATTCATTCCCC---AAAAATGAAAAATTGGAAAATACC----------------
rfbat	TGCTTAACAC-----------GCACACCTAATTTGTATAAAAGCAAATTCATTGTTC----TAAAAACAAAGCCTGAAAATACTAGACAAACCAAAAGAA
hedgehog	--
shrew	--
armadillo	TTATCCGCATAGGCTAACTGGGCATGCATTACTTTT--AGCAGCAGG------------------------------ATATACCACACAAAACAAAAAAA
elephant	--
tenrec	--
monodelphis	--
platypus	--
chicken	--
xenopus	--
tetraodon	--
fugu	--
zebrafish	--

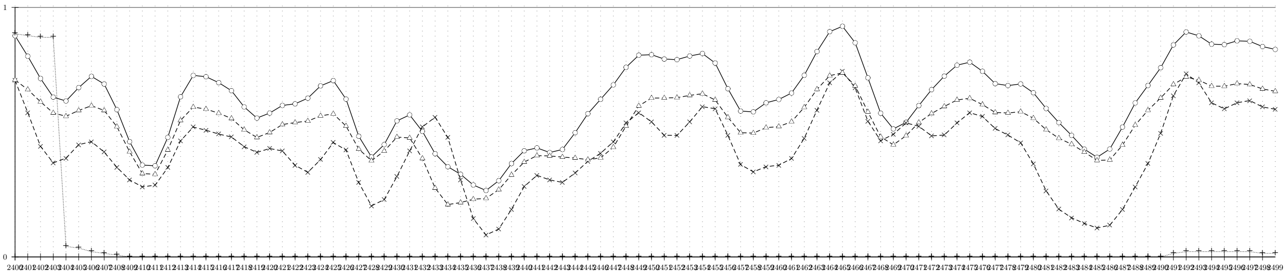

human	TAAGTCAAAGCTTCAGTACCGAGAAGTTAACACTGTTAACATCATCTGAGTTAACTATTACCCTTCTGATGTATTCATGCTACTCTATTTGAGTAAAATG
chimp	TAAGTCAAAGCTTCAGTACCGAGAAGTTAACACTGTTAACATCATCTGAGTTAACTATTACCCTTCTGATGTATTCATGCTACTCTATTTGAGTAAAATG
colobus	--
baboon	TAAGTCAAGGCCTCAGTACCGAGAAGTTAACACTGTTAACATTGTCTGAGTTAACTATTAACTTTCTGATGTATTCATGCTGTTCTATTTGAGTAAAATG
macaque	TAAGTCAAGGCCTCAGTACCGAGAAGTTAACACTGTTAACATTGTCTGAGTTAACTATTAACTTTCTGATGTATTCATGCTGTTCTATTTGAGTAAAATG
dusky titi	--
owl monkey	--
marmoset	TAAGTCAAAGCCTCACTACCGAAAAGTTAGCACCGTCCACATCACCTGAGTTAAGTACTAACCTTCTGAGGTATTCATGTTAT--TGTTTGAGTAAAATG
mouse lemur	--
galago	TAGAACTAAAAATCATTA---ATAACTCTACTCCACAGAGGTGACCTCAGTTAACTGGTAATCTTCTTACATGTCCATGCTGTCCACTGTCAGTAAAAT-
rat	--
mouse	--
rabbit	TAAATCAAAGCCCCACGATGCAGAGGTGGGCCCTGCTGCCCT---GTCAGAGAACTGCCAGGCCTCTGACGCAGCCAGGCCACTCACCTCCAGTGAGAAG
cow	-GAACCATAACACCACCACCCAAAAGTTCATAATGTCTGGTTCCC---AGTTGACTGTTAACCTTCTGGTATGTCCACACTGTTA-CATTTAGTAAAATG
dog	--------ACTGTTACTAAATAACAGTGT-TAACCTCTAGATTAC------TAGCTGTGAGCCTTCTGATATATCCATACTGTTCTCTTTTAGTAAAATG
rfbat	TGAAACATAACTCCACTACCCAGAGGT---TAACTTCCAGGTTACC--AGTTAACTGCTAACCTTCTGCTGTATCTATACTGTTAATTTTTAGTAAAATA
hedgehog	--
shrew	--
armadillo	TAAACCATAATCCCACTACTCAGAAGTTAACACTATTAGCAT---GTGGGTTAACTGTTAACCTTCTGGTGTATCCAAACTGTGAATTTTTAGTCAAATG
elephant	--
tenrec	--
monodelphis	--
platypus	--
chicken	--
xenopus	--
tetraodon	--
fugu	--
zebrafish	--

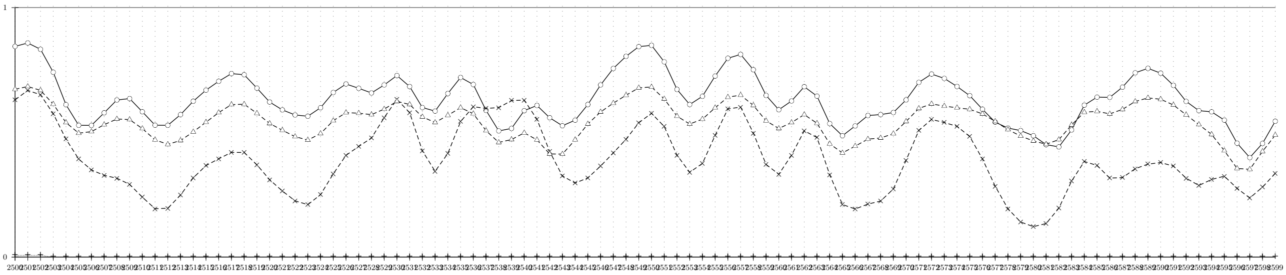

human	AGATCATACTTTATAGTTTTATAGTTTACACCCAATCATATATCACTAAAAAACTTTGAAATCATTAAGTGCAGCATGTTTTTTTTCTTTTTGAGACAGG
chimp	AGATCATACTTTATAGTTTTATAGTTTACACCCAATCATATATCACTAAAAAACTTTGAAATCATTAAGTGCAGCATGTTTTTTTTCTTTTTGAGACAGG
colobus	--
baboon	AGATCGTACTTTACAGTTTTATAGTTTACACCTGATCATGTATCACTAAAAAACTTTGAAACCATTAAGTACAGCAT-TTTTTTTTCTTTTTGAGACAGG
macaque	AGATCGTACTTTACAGTTTTATAGTTTACACCTGATCATGTATCACTAAAAAACTTTGAAACCATTAAGTACAGCAT-TTTTTTTTCTTTTTGAGACAGG
dusky titi	--
owl monkey	--
marmoset	AGATCGTACTTTACAGTTTTATACTTTACA-CTGATCATCTAACACTAAAAAACTCTGAAATCATTAAGTACAGCAT-TTTTTTTCCTTTTTGAGACCAG
mouse lemur	--
galago	---------TTTACAGTTTTATATTTTACACCTATTCACGTAACATTAAAAAATTCTGACATCACTGAATACAACAT-CTTTATTCCATCCTATGACAAG
rat	--
mouse	--
rabbit	GGATCACCCTTCTGCAGCTGGCAGGCTACG-CCAATCACGCATCACAAGGAAACCCGGATGCCAGCAGCGACATCGC-CCCA------------------
cow	GGATCATATTTACCAGTTTTAT-AGTTACACCTAATCCTATACCCTTAAAAAA-TCTGGTATCAGTAAGTATA-----TTTCTATCCTTTTTGATACCTG
dog	GGGTA---CTTGACAGCTTTAC-GTTTATACCTAATTGCATACTGTTTAAAAG-GCTGATGTCAGGAAGCATA-----TGTCCATCATTTTTAATAGCTG
rfbat	GGATCATACAGAACTGTTTTGTTGTTTATACCCAATCATATATTGTTAAAAAG-TCTGATGTCAACCAGTATA-----TTCCCTTCTTTTTTAACAGGTG
hedgehog	--
shrew	--
armadillo	GGATAGTG---TACAGTTTCATAGTTGTTCCCCAGTCATATATCATTAAAAAC-TCTGATGGCAATAAATGTA-------TT------------------
elephant	--
tenrec	--
monodelphis	--
platypus	--
chicken	--
xenopus	--
tetraodon	--
fugu	--
zebrafish	--

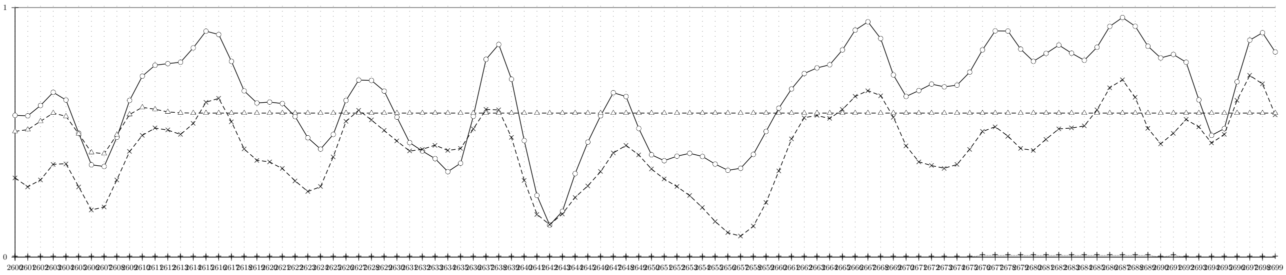

human	GCCTCCCTCTGTCCTCCAGGCTAGAGTGCAGTGGCGCAATCTCCACTTACTGCAGCCTTGACATCCCAGGCTCAAGCAATTCTCCCACCTCAGCTTCCCA
chimp	GCCTCCCCCTGTCCTCCAGGCTAGAGTGCAGTGGCGCAATCTCCACTTACTGCAGCCTTGACATCCCAGGCTCAAGCAATTCTCCCACCTCAGCTTCCCA
colobus	--
baboon	GCCTCCCTCTGTCCTCCAAGCTAGAGTGCAGTGGTGCAATCTTGACTTACTGCAGCCTTGACATTCCAGGCTCAAGCAATTCTCCCACCTCAGCCTCCCA
macaque	GCCTCCCTCTGTCCTCCAAGCTAGAGTGCAGTGGTGCAATCTCGACTTACTGCAGCCTTGACATTCCAGGCTCAAGCAATTCTCCCACCTCAGCCTCCCA
dusky titi	--
owl monkey	--
marmoset	GCCTCC--CTGTCCTCCAGGCTAGAATGTAGCGGCACAATCCTGACTTACCACAGCCTTGACATTCCAGGTTCAAGCAATTCTCCCACCTCAGCCGCCCA
mouse lemur	--
galago	TTTGCTCAATCTCTTCCAGACAATTATG-AGGAGTCCAATTTTCAGCTATCGTGAAAAAAAAATTCC-----CCAACAATACTCACACCTTAGCTTCCCA
rat	--
mouse	--
rabbit	--
cow	CATTCCTCCT--
dog	CATTCCATCT--
rfbat	CATTCTACCT--
hedgehog	--
shrew	--
armadillo	--
elephant	--
tenrec	--
monodelphis	--
platypus	--
chicken	--
xenopus	--
tetraodon	--
fugu	--
zebrafish	--

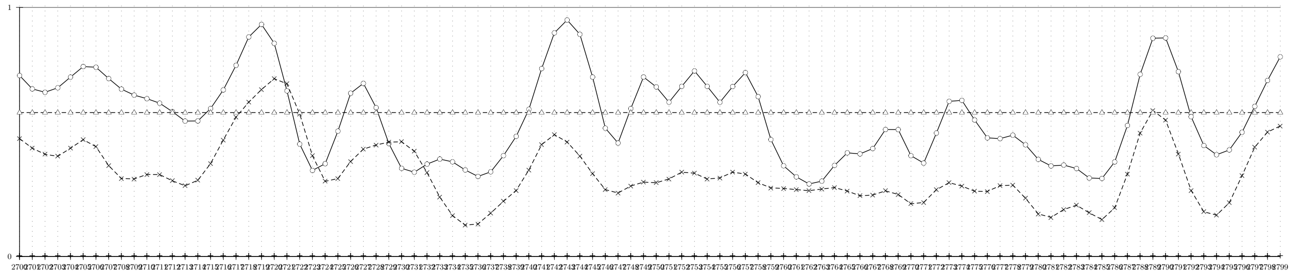

```
human        AGTAGCTGAGACAACAGGTACACGCCAACACACCCAGCTAACTTTTGTTTTGTTTGTTTGTTTGTTTGTTTGTTTTGGTAGAGACAGAATCTCACTATGT
chimp        AGTAGCTGAGACAACAGGTACACGCCAACACACCCAGCTAACTTTTGTTTTGTTTGTTTGTTTGTTTGTTTGTTTTGGTAGAGACAGAATCTCACTATGT
colobus      ----------------------------------------------------------------------------------------------------
baboon       AGTAGCTGAGACAATAGGTACACGCCAACACGCCCAGTTAACTTTTG---------------TATTTTTTTCTTCTGGTAGAGATAGAATCTCACTATGT
macaque      AGTAGCTGAGACAATAGGTACACGCCAACACGCCCAGTTAACTTTTG---------------TATTTTTTTCTTCTGGTAGAGACAGAATCTCACTATGT
dusky titi   ----------------------------------------------------------------------------------------------------
owl monkey   ----------------------------------------------------------------------------------------------------
marmoset     AGTAGCTAAGACAAC----ACACACCAACATGCCCAGCTAACTTTTT---------------------TTTTTTTTTGTAGAAACAGAATCTCACTATGT
mouse lemur  ----------------------------------------------------------------------------------------------------
galago       TGGAACTGATAAATCAAG--CATCTTAAAAGGTTTTTTTTTCTTTTTCTTTCTTTCTTTCTGTCTCTCTTATTTTTTTTTTAATTTTATCTTTTCTTAT
rat          ----------------------------------------------------------------------------------------------------
mouse        ----------------------------------------------------------------------------------------------------
rabbit       ----------------------------------------------------------------------------------------------------
cow          ----------------------------------------------------------------------------------------------------
dog          ----------------------------------------------------------------------------------------------------
rfbat        ----------------------------------------------------------------------------------------------------
hedgehog     ----------------------------------------------------------------------------------------------------
shrew        ----------------------------------------------------------------------------------------------------
armadillo    ----------------------------------------------------------------------------------------------------
elephant     ----------------------------------------------------------------------------------------------------
tenrec       ----------------------------------------------------------------------------------------------------
monodelphis  ----------------------------------------------------------------------------------------------------
platypus     ----------------------------------------------------------------------------------------------------
chicken      ----------------------------------------------------------------------------------------------------
xenopus      ----------------------------------------------------------------------------------------------------
tetraodon    ----------------------------------------------------------------------------------------------------
fugu         ----------------------------------------------------------------------------------------------------
zebrafish    ----------------------------------------------------------------------------------------------------
```

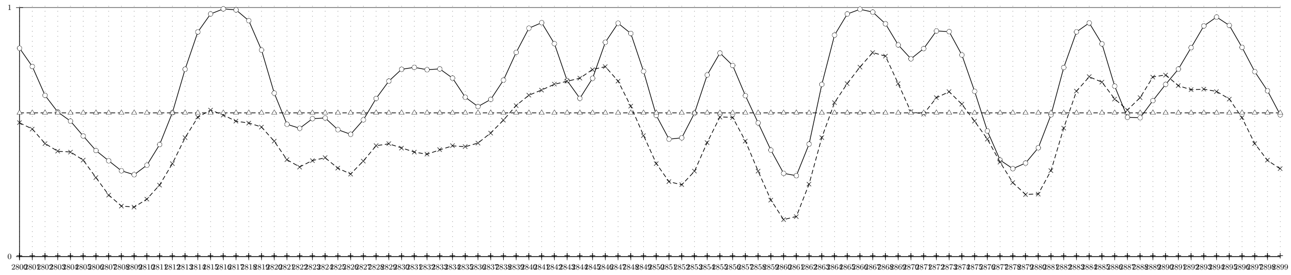

```
human        TGTCCAGGATGGTCTCAAGAACTCCCGGGCTCAAGCAATCCTCCAGCCTCCAACTCCCAAAATGCTGGGATTACAGGTGTGAGCCACGACGGCCAGCCTC
chimp        TGTCCAGGATGGTCTCAAGAACTCCCGGGCTCAAGCAATCCTCCAGCCTCCAACTCCCAAAATGCTGGGATTACAGGTGTGAGCCACGACGGCCAGCCTC
colobus      ----------------------------------------------------------------------------------------------------
baboon       TGCCCAGGACGGTCTCAAGAATTCCTGGGTTCAAGCAATCCTCCCGCCTCCACCTCCCAAAATGCTGGGATTACAAGCGTGAGCCACCACGGCCAGCCTC
macaque      TGCCCAGGACGGTCTCAAGAATTCCTGGGTTCAAGCAATCCTCCCGCCTCCACCTCCCAAAATGCTGGGATTACAGGCGTGAGCCACCACGGCCAGCCTC
dusky titi   ----------------------------------------------------------------------------------------------------
owl monkey   ----------------------------------------------------------------------------------------------------
marmoset     TGCCCAGGATGGTCTCAAGAATTCCTTCGCTCAAGCAACCCTCCCGCCTCCACCTCCTAGAGTGCTGGGATTACAGGTGTGAGCCACCACGGCCAGCCTC
mouse lemur  ----------------------------------------------------------------------------------------------------
galago       TGCTCCCAGCAACCTCA--AACTACTGGGCTCCAGTGATCCTCCTGCCTCAGCTTCCTAAGTAGCTGGGAATACAGGAACCTGCCACTATGACCAGCT--
rat          ----------------------------------------------------------------------------------------------------
mouse        ----------------------------------------------------------------------------------------------------
rabbit       ----------------------------------------------------------------------------------------------------
cow          ----------------------------------------------------------------------------------------------------
dog          ----------------------------------------------------------------------------------------------------
rfbat        ----------------------------------------------------------------------------------------------------
hedgehog     ----------------------------------------------------------------------------------------------------
shrew        ----------------------------------------------------------------------------------------------------
armadillo    ----------------------------------------------------------------------------------------------------
elephant     ----------------------------------------------------------------------------------------------------
tenrec       ----------------------------------------------------------------------------------------------------
monodelphis  ----------------------------------------------------------------------------------------------------
platypus     ----------------------------------------------------------------------------------------------------
chicken      ----------------------------------------------------------------------------------------------------
xenopus      ----------------------------------------------------------------------------------------------------
tetraodon    ----------------------------------------------------------------------------------------------------
fugu         ----------------------------------------------------------------------------------------------------
zebrafish    ----------------------------------------------------------------------------------------------------
```

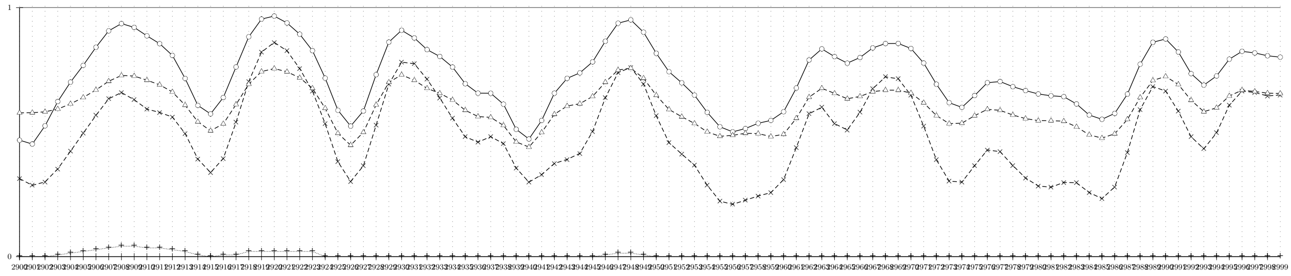

```
human        CAGAGTATTTTTAATCGCTGCATTCCATCTTATGACCAGTCCCCAATTTGCTCAGCCAATCCCTTACTGGTGGATAGTTAGAAGACCCATTTCCAGCTAA
chimp        CAGAGTATTTTTAATCGCTGCATTCCATCTTATGACCAGTCCCCAATTTGCTCAGCCAGTCCCTTACTGGTGGATAGTTACAAGACCCATTTCCAGCTAA
colobus      ----------------------------------------------------------------------------------------------------
baboon       TAGAGTATTTTTAATCGCTGCATTCCGTCTTATGACCAGTCCCCTATTTGCTCAGCCAATCTCTTAATGGTGGATAGTTAGAAGTCCCATTTCCAGCTAA
macaque      TAGAGTATTTTTAATCGCTGCATTCCGTCTTATGACCAGTCCCCTATTTGCTCAGCCAATCTCTTACTGGTGGATAGTTAGAAGTCCCATTTCCAGCTAA
dusky titi   ----------------------------------------------------------------------------------------------------
owl monkey   ----------------------------------------------------------------------------------------------------
marmoset     TATAATATTTTTAATTACTGCATTCCATCTTATGACCAGTTTTAATTTGCTCAGCTAATCCCTTACTGGTGGACAGTTAGAAGTCCCATTTCTAGCTAA
mouse lemur  ----------------------------------------------------------------------------------------------------
galago       ---AGTTTTTTT----------TTCTATTTT---------------TTTGTAGAGATGGGGTCTGGCTCTTG------------------CTCAGGCTAG
rat          ----------------------------------------------------------------------------------------------------
mouse        ----------------------------------------------------------------------------------------------------
rabbit       ----------------GCTGCACTTCCTCTTATGGCTGGCGCTCAGTTTGCTCAACTAATCCCTTACTGGTATACAGTTAGGAGCCCAATTTTCAGCGAA
cow          -------TTTTTGATACCTGCATTCCTCCTTATGACCAGTGCTCAATTTGCTCAGTC----CCTTGCTGGTGGGTAGTAAGGAGTCCGATTTTCAGCTAA
dog          -------TTTTTAATAGCTGCATTCCATCTTACGATTAGCCCTCAGTTTGCACAATCAATGCCTTGCTGGTGGACAGTTAGGAGTTCAATTTTCAGCTAA
rfbat        -------TTTTTAACAGGTGCATTCTACCTTATGACCAGACCTCAATTTGCTCGGTCGATACCTTGCTGGTGGACAGTTAG----CTAATTTTCAGCTAA
hedgehog     ----------------------------------------------------------------------------------------------------
shrew        ----------------------------------------------------------------------------------------------------
armadillo    -----TATTTCTAAGAGCTGCATTCCCTCTTACGACTAGCTCTCAATTTGCTCAACCAATCCCTTGCTGGTGCATTAGGAAATGCTCAATTTTCGGTTAA
elephant     ---------------------------------------------------------------------------TGGTGGAATTCTTATTTTTAGCTCA
tenrec       ----------------------------------------------------------------------------------------------------
monodelphis  ----------------------------------------------------------------------------------------------------
platypus     ----------------------------------------------------------------------------------------------------
chicken      ----------------------------------------------------------------------------------------------------
xenopus      ----------------------------------------------------------------------------------------------------
tetraodon    ----------------------------------------------------------------------------------------------------
fugu         ----------------------------------------------------------------------------------------------------
zebrafish    ----------------------------------------------------------------------------------------------------
```

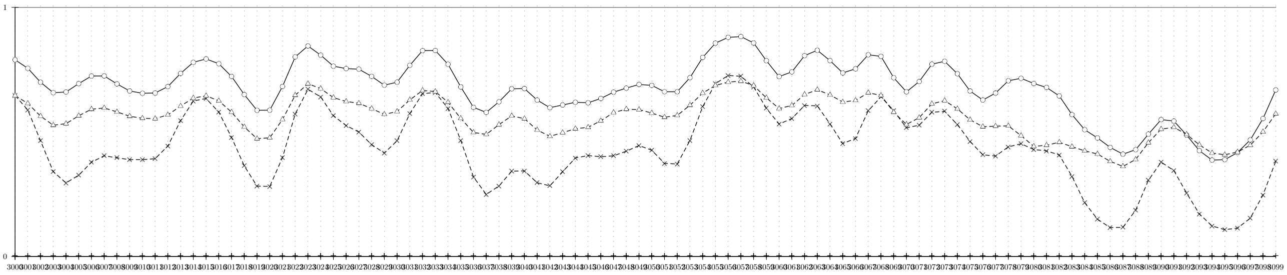

```
human       TATAAATATTATTCCCTAACAAAACCTCTACTCACATCTTTAATTATTTCACTGGGATAAATGACTAGACATGGAATTGCAGGATCAAGTATCTTAAAAG
chimp       TATAAATATTATTCCCTAACAAAACCTCTACTCACATCTTTAATTATTTCACTGGGATAAATGACTAGACATGGAATTGCAGGATCAAGTATCTTAAAAG
colobus     ----------------------------------------------------------------------------------------------------
baboon      TATAAATATTATTCCCTAACAAAACTTCTACTCACATCTGTAATTATTTCACTGGGATAAATGACTAGACATGGAATTGCAGGATCAAGTATCTTAAAAG
macaque     TATAAATATTATTCCCTAACAAAACTTCTACTCACATCTGTAATTATTTCACTGGGATAAATGACTAGACATGGAATTGCAGGATCAAGTATCTTAAAAG
dusky titi  ----------------------------------------------------------------------------------------------------
owl monkey  ----------------------------------------------------------------------------------------------------
marmoset    TATAAAGAATATTCCCTAATAAAACCTCTACTCACATCCTTAATTATTTCACCGGGATAATTGACTAGACATGGAACTGCTGGATCAAGGATCTTAACAG
mouse lemur ----------------------------------------------------------------------------------------------------
galago      TCTTGA----ACTCCTGACCTTAACTGATCCTCCCACTTTGGCCTCCCAGAGTGG----------------TAGAATTACAGGCCTGAGCATGCCCAGAT
rat         ----------------------------------------------------------------------------------------------------
mouse       ----------------------------------------------------------------------------------------------------
rabbit      CATGAAAAATCTTCCCTAACAAAACCTCTTCGCACACTCTTAATTCTTTCCTTAGTACAAG-GACCAGACGTGGGACTGCTG-CTCAAACAGCCTAGGGG
cow         TAGAAAAAGTATCCCCTAACAAAACCTCTACTCAACACCTTAATTATTCCCTTAGGATAAACGGCTGGACGTGGAATGGCTGG-----------------
dog         TATGAAAAATATCCCCTAAGAAAACCTCTACTCACATCCTTAATTCTTCCCTTAGGATAAATGACTAGACATGGAAAGGCCAGAATGTG-----------
rfbat       TATGGAAAACAGCCCCTAACCAAACCTCTAGTCACATCCTTCATTATTTCCTCAGGATAAATGACCAGACATGGGATGGCTGGCTCAGT----TCAAAAG
hedgehog    ----------------------------------------------------------------------------------------------------
shrew       ----------------------------------------------------------------------------------------------------
armadillo   TATGAAAAATATCCTCTAACCCAATCTCCATTCACATCCTTAGGTGTTTCCTTGGGATAAGTGACTGGACCGGGCACTGCTGGGTCCCACACCTGGGAAA
elephant    TATGAGAAACATGGCCTTCCCAAATCTCTATTCACATTCTTACTTATTTCCTTAG-------------AGGTGGAACTCCTAGGTCAAAC----------
tenrec      ----------------------------------------------------------------------------------------------------
monodelphis ----------------------------------------------------------------------------------------------------
platypus    ----------------------------------------------------------------------------------------------------
chicken     ----------------------------------------------------------------------------------------------------
xenopus     ----------------------------------------------------------------------------------------------------
tetraodon   ----------------------------------------------------------------------------------------------------
fugu        ----------------------------------------------------------------------------------------------------
zebrafish   ----------------------------------------------------------------------------------------------------
```

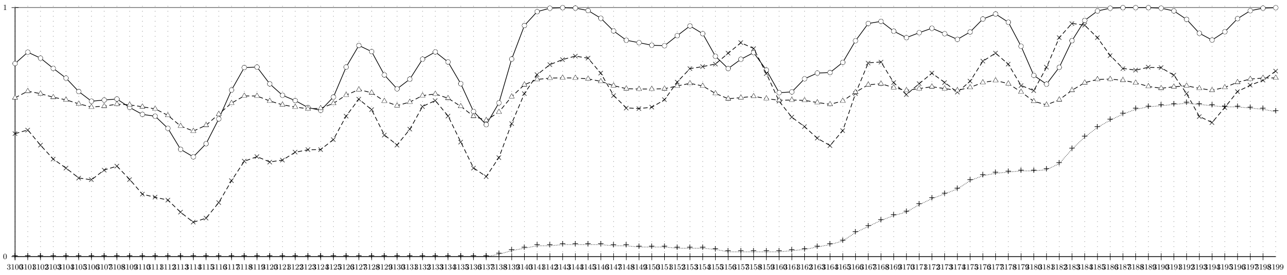

```
human       TTTTTTTATCTTTTTTCTTTATCTATTGTGCAACCCACTTCAGTGCCAGCAGCCTACGGGAACAGAGGCCAGGCAATGGTTCAGGGCTACAGTTTGCTCT
chimp       TTTTTTTATCTTTTTTCTTTATCTATTGTGCAACCCACTTCAGTGCCAGCAGCCTACGGGCACAGAGGCCAGGCAATGGTTCGGGGCTACAGTTTGCTCT
colobus     ----------------------------------------------------------------------------------------------------
baboon      TTGTTTTATCTTTTTTCTTTATCCACTGTGCAACCCACTTCAGTGCCAGCAGCCTATGGGCACAGAGGCCAGGCAATGGTTCGGGGCTACAGTTTGCTCT
macaque     TTGTTTTATCTTTTTTCTTTATCCATTGTGCAACCCACTTCAGTGCCAGCAGCCTACGGGCACAGAGGCCAGGCAATGGTTCGGGGCTACAGTTTGCTCT
dusky titi  ----------------------------------------------------------------------------------------------------
owl monkey  ----------------------------------------------------------------------------------------------------
marmoset    TT-TTTTATCTTTCTTCCTTATCCA-TGTGCAACCCCAGTCAGTGCCAGCAGCCTACGGGCACACAGGCCAGGCAATGGTTCGGGGCTACAGTTTGCTCT
mouse lemur ----------------------------------------------------------------------------------------------------
galago      TTTCTTTTTTCCTCATCTTTTTCCACTGTACGACCCACTTCAGTGCCAGCAACCTATGGGTAGAGAGGCCTGGCCATGGTTCGGGGC--CAGTTTGCTCT
rat         ----------------------------------------------------------------------------------------------------
mouse       ----------------------------------------------------------------------------------------------------
rabbit      TTTTTCCATTTTTTCCTTTTTCCTACTGTGGAACACATTTCAGTGCCAACAACCTACGG--ACAGTGGCCAGGCAATGGTGTGGGGCTAC-GTTGGCTCT
cow         ----------------------------------------------------------------------------------------------------
dog         ----------------------------------------------------------------------------------------------------
rfbat       TTT-------------------------------------------------------------------------------------------------
hedgehog    ----------------------------------------------------------------------------------------------------
shrew       ----------------------------------------------------------------------------------------------------
armadillo   TTTT------------------------------------------------------------------------------------------------
elephant    ----------------------------------------------------------------------------------------------------
tenrec      ----------------------------------------------------------------------------------------------------
monodelphis ----------------------------------------------------------------------------------------------------
platypus    ----------------------------------------------------------------------------------------------------
chicken     ----------------------------------------------------------------------------------------------------
xenopus     ----------------------------------------------------------------------------------------------------
tetraodon   ----------------------------------------------------------------------------------------------------
fugu        ----------------------------------------------------------------------------------------------------
zebrafish   ----------------------------------------------------------------------------------------------------
```

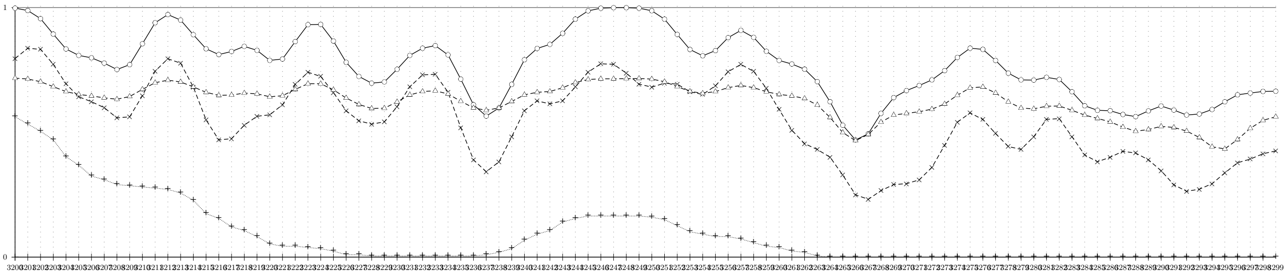

```
human       GGCATGGGGTACTATGAGAGGTCCAGGGGGACGGGCCCATCATAAATCCAAACCCATGCATAAGCACCTTAAAAGTTTTGATGTGTACAGCCAAACTTCC
chimp       GGCATGGGGTACTATGAGAGGTCCAGGGGGACGGGCCCATCATAAATCCAAACCCATGCATAAGCACCTTAAAAGTTTTGATGTGTACGGCCAAACTTCC
colobus     ----------------------------------------------------------------------------------------------------
baboon      GGCATGGGGTACTATGAGAGGCCCAGGGGGACGGGCCCATCATAAATCCAAACCCATGCATAAGCATCTTAAAAGTTTTGATGCGTACAGCCAAAC-TCC
macaque     GGCATGGGGTACTATGAGAGGCCCAGGGGGACGGGCCCATCATAAATCCAAACCCATGCATAAGCATCTTAAAAGTTTTAATGCGTACAGCCAAAC-TCC
dusky titi  ----------------------------------------------------------------------------------------------------
owl monkey  ----------------------------------------------------------------------------------------------------
marmoset    GGCATGGGGTACTATGAGAGGCCCAGGGGGACAGGCCCATCATAAATCCAAACCCATGCATGAGCATCTTAAACGTTTTGATGTGTACGGCCAAACTTCC
mouse lemur ----------------------------------------------------------------------------------------------------
galago      GGCACGGGGCACTATGACAGGCCCAGAAGTACAGGCCTATCAGAAAT-CAAACCCGTGCGTAAGCATCTTGAA-GCTTTGATGTACACGGCCAAACTGCC
rat         ----------------------------------------------------------------------------------------------------
mouse       ----------------------------------------------------------------------------------------------------
rabbit      GGCATGAGATACTATAAGAGACCCAGGGGAACAGGTTTGCCATAAATCCAAACTCATGCATAAACAGCCTAGGGGTTTTGACG-----------------
cow         ---------------------------------------------------------------------------------------CAGCTCAGCTGCC
dog         ----------------------------------------------------------------TGTGTTTAAAGTTCTGATGCATACGGCCTAAATGCC
rfbat       ----------------------------------------------------------------CAGTTCAAAAGTTTCAACGTATCCACATCCGATGGC
hedgehog    ----------------------------------------------------------------------------------------------------
shrew       ----------------------------------------------------------------------------------------------------
armadillo   -------------------------------------------------------------------------------GCTG-----------------
elephant    ----------------------------------------------------------------CGTTTTAAAAGTTTTGATG--TTCAGCCAAACTATC
tenrec      ----------------------------------------------------------------------------------------------------
monodelphis ----------------------------------------------------------------------------------------------------
platypus    ----------------------------------------------------------------------------------------------------
chicken     ----------------------------------------------------------------------------------------------------
xenopus     ----------------------------------------------------------------------------------------------------
tetraodon   ----------------------------------------------------------------------------------------------------
fugu        ----------------------------------------------------------------------------------------------------
zebrafish   ----------------------------------------------------------------------------------------------------
```

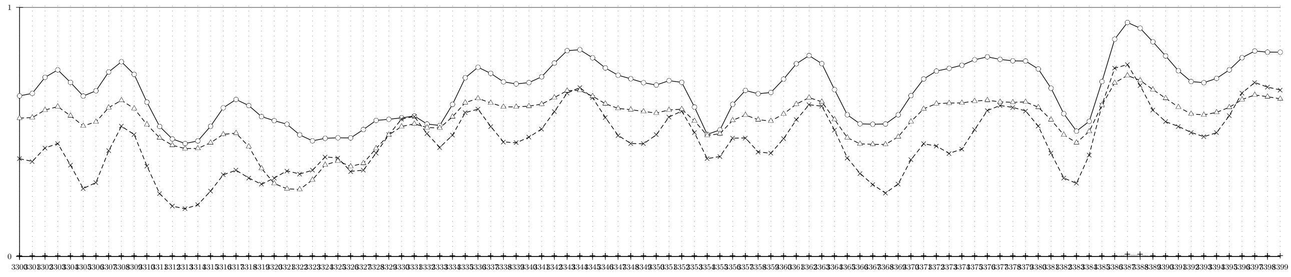

human	CAGCAGAAACATTAAACTGTAGTATGTTCCAGCCCCCTTCAACAGTGTAAATGGTACCAACTGTCACTGAATAAAAGGCCAGACAGCTGAAATGGCCCAC
chimp	CAGCAGAAACATTAAACTGTAGTACGTTCCAGCCCCCTTCAACAGTGTAAATGGTACCAACTGTCATTGAATAAAAGGCCAGACAGCTGAAATGGCCCAC
colobus	--
baboon	CAGCAGAAACATTAAACTGTAGTATGTTCCAGTCCCCTTCAACAGTGAAAATGGTACCAACTGTCCTGGAATAAAAGGCCAGATGGCTGAAATGACCCAG
macaque	CAGCAGAAACATTAAACTGTAGCACGTTCCAGTCCCCTTCAACAGTGAAAATGGTACCAACTGTCCTGGAATAAAAGGCCAGATGGCTGAAATGACCCAG
dusky titi	--
owl monkey	--
marmoset	CAGCAGCAACATTCAACTGTAGCACATTCCAGTCCCCTTCAACAGTGTAAATGGTACCACCTGTCCTTGAATAAAAGGCCAGACAGCTGAAATGACCCAG
mouse lemur	--
galago	CAGCACAAACATCATGCTACAGTACACTCT-GCGTCCTTCAACAGTGTAAATGGTACCCGCTGTCTTTATATAAAAGGCCAGATGGCTGAGATGACCCAG
rat	--
mouse	--
rabbit	---CAGAAAGCCCGCACTGCCGGACGTGCTG--CCCCTTCGGCAGTGTAAACGGAGCTCACTGTCCTTCAGCAGCAGGCCAGGCGGCTGAGAGGGCCCGG
cow	CTGCTGAAACTTTA-CCAGAAGTACATCCTTGC-TCCTTTAGCAGTGTGGACGACACCAAATGTCCTTTAATAAAA-GCCAGACA-CTGAGAAGACGCAG
dog	TTGC-AAAACACCA-ATTGAAGTGCAGCCCTG--TCTGTTAGCAGTGTAGGTGACCCCAAAAGTCTTTTAATGAAA-GTCAGACAGCTGAGATGAGCCAG
rfbat	CGGC-AGGACACTA-ACTGAGGTGCATCCCTGCACCCTCTAACAGTGTAGATGAGCCCAAATGTCCTTTCATAAAC-GGCCAATGGCTGTGACGACCCAG
hedgehog	--
shrew	--
armadillo	----------------CAGTTGGCCAAACTG---CCCTCTAGAAACACAACTGAAGCCCACTCTTACT---------CCCACAT-------------CAT
elephant	TTCCAGAAACACTATCC---
tenrec	--
monodelphis	--
platypus	--
chicken	--
xenopus	--
tetraodon	--
fugu	--
zebrafish	--

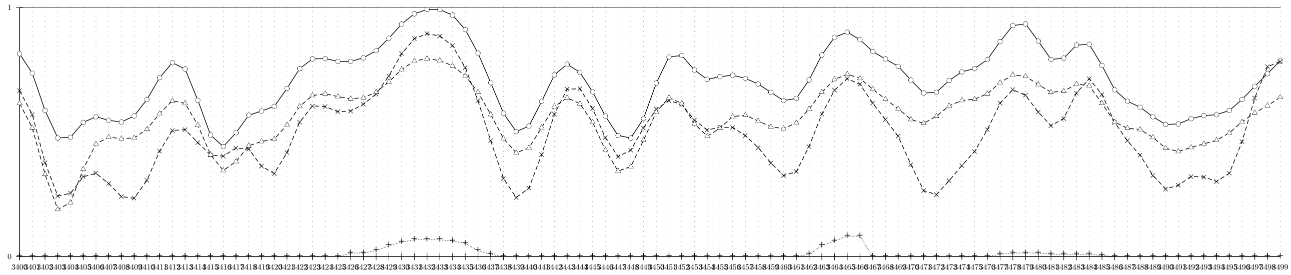

human	GGGAATTTGAAGAAACTACTGGCCCTGGCAGCAGCAAGATATCCTGAACCTAGTGCCTGGTAACAAGTAGGAAAAAAAAAAATCTGTCCGCTGCATTATG
chimp	GGGAATTTGAAGAAACTACTGGCCCTGGCAGCAGCAAGATATCCTGAACCTAGTGCCTGGTAACAAGTAGGAAAAAAAAAAATCTGTCTGCTGCATTATG
colobus	--
baboon	GGGAGTCTTAAGAAACTACTGGCTCTGGCAGCAGCAAGACATCCTGAACCTAGTTCCTAGTAACAAGTAGG--AAAAAAAAATCTGTCTGCTGCACTATG
macaque	GGGAGTCTTAAGAAACTACTGGCCCTGGCAGCAGCAAGACACCCTGAACCTAGTTCCTAGTAACAAGTAGG--AAAAAAAAATCTGTCTGCCGCATTATG
dusky titi	--
owl monkey	--
marmoset	GGGGATTTTAAAAAACAACTGGCCCTGGCAGCAGCAAGGCATCCTGAA-CTAGTTCCCGGTAACAAGCAGG-AAAAAAAAAATCTGTCTGTGGCATCATG
mouse lemur	--
galago	GGGAATTTTAAGAAACCGCTAGCCCTG---GCAGCAAGACATCCTGACTCTAGTTCCTGCTCACAAGTAGGAAAAAAAAAAACCTGTCTGCTGCATCATG
rat	--GTACCTGTTA-----CAGCAAAAAGAAAAGTC--CCTAATGCATTATG
mouse	---TTGTTA-----CAGCAAAAAGAAAAGTC--ACTAATGTATTAAG
rabbit	GGAGCTGCGAGGAAACCTCCAGCCCCGGCAGCAGCAGGATGTCCT-ACCCTGGTTCCTGGCAACCAGCAGTGGAAACA-----CTGTCTGCTGTGT----
cow	GGGAATTCTGAGAAATGCCCAGCCCTGGAAGCAG-AGGGCATCCCACGCA------CTGGCCACAAGCAGG------A----CCTGGGCACGACACCACG
dog	GGGAATTCTGCGAACT-CCTAGCCCTGGCAGCAGCAGCAGTTCCCACAGG--G---CTGGTCCCAAGCAGG------A--------------AAGGCACG
rfbat	GGATATCCTGAGAAACTCCGAGCCCT-GCGGCAGCAGGACACCCCACATG--GGACCTGGCAACAAGCAGG------A-----CTGTCCACTGCACCAGG
hedgehog	--
shrew	--
armadillo	GGTCCTTCCATGAAACCCCTGCCTCTGGCAGCAGCAGGAAACCACGCCC--A--ACCTGGCAACAAGCTGTGGGGTCA------------CCGCA-----
elephant	--------TGAGTAGATCCTCACCCCATCAGCA--------TCCCACAC--AGGACTCCGCAGCAAGCGGGAAAAACA----TCTGCCCATTGTACC-CG
tenrec	--
monodelphis	--
platypus	--
chicken	--
xenopus	--
tetraodon	--
fugu	--
zebrafish	--

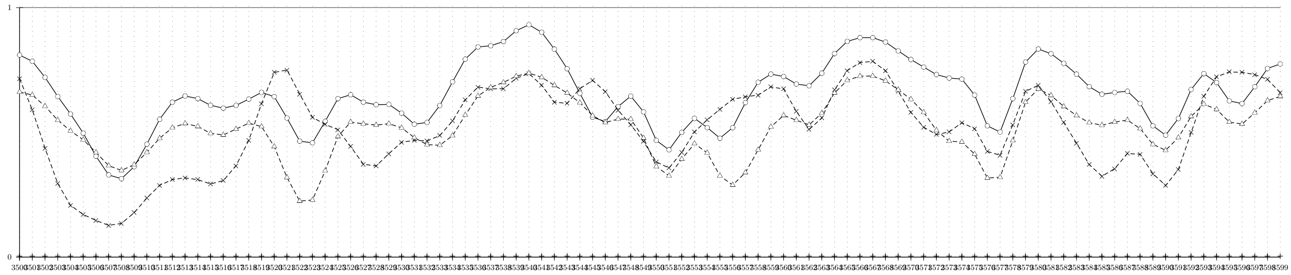

human	CAGAGACCACATCTCCACTCTCATGCTGCTCCACCACAGCAGCAATAGAGCGCCCCACACAGACCAGGCTGACCACATACAGGGTGACCGCCACCTGCAC
chimp	CAGAGACCGCATCTCCACTCTCATGCTGCTCCACCACAGCAGCAATGGAGCGCCCCACACAGACCAGGCTGACCACATACAGGGTGACCGCCACCTGCAC
colobus	--
baboon	CAGAGACCACATCTCCACTCTCACGCTGC-CCACCACAGCAGCAATGGAGGGCCCCACACAGACCAGGCTGACTACAGACAGGGTGACCACCACCCGCAC
macaque	CAGAAACCACATCTCCACTCTCACGCTGC-CCACCACAGCAGCAATGGAGGGCCCCGCACAGACCAGGCTGACTACAGACAGGGTGACCACCACCCGCAC
dusky titi	--
owl monkey	--
marmoset	CAGAGACCACTT------------------CTACCACAGCAGTGACGGAGG--CCTGCGCAGACCAGGCTGACCACACACAGGGTGGCCACC----GCAC
mouse lemur	--
galago	CAG------CACCCCTGTCCCCACGCTGCTCTACCACAGCAG---TGAAGACCCCCCCACAGATCAAGCTGAC-------------ACCATC--------
rat	CAAA--CAGTCTATCTCCTCCTGTCATGCGCTGCC-CAACAGCAACGGCGCCCC---CCCAGCCCAGGCTGG-CACGTACAGTCTC-CTTCCCCTCGCAC
mouse	CAAATGCAATCTCTCTCCTCCCCTCACGCTCTGAC-CAACAGCAACAGAGCCAC---CACGGCCCAGGCTGACCACAGACAGTCTCACTTTTACTCAGAC
rabbit	------------CTCCTCCCCAGAACTACTCC----------------------ACTCCCAGGCCAGGCGGACCACACACAGGGGC-CCTGCACCTGCAC
cow	GGAAGGAGGTGTGTCCTCCACGGGGCTAG-CCACC------------------------CGGCCCCGGCTGACCACCTCCAGGG--ACCCGTACCCGCAG
dog	CAATGAAGTTAT---CTCCACGGTGCTAC-GCGCA-------------------GCGCACGGACCAGGCTAATA---TACAGGGTGATCAGCACCTACAC
rfbat	CAAAGAGAGCATCTTCTCTACGGGGCTGC-CTGCA-------------------CTGCACAGGCCAGGCGGGCAGCAGACAGGGTGACCACCGCCCACGC
hedgehog	--
shrew	--
armadillo	--C--AAAGCAGGCTGACCACCAACGGTGACGC------------
elephant	CAATGAGCATGTCCCTTCTCCGGAGCTTT---TCCACGGCAGCAACAGAGTGGCCTGCACAGACCAGACTGAC-ACCTACATGGTGATCACCACCCACAC
tenrec	--
monodelphis	--
platypus	--
chicken	--
xenopus	--
tetraodon	--
fugu	--
zebrafish	--

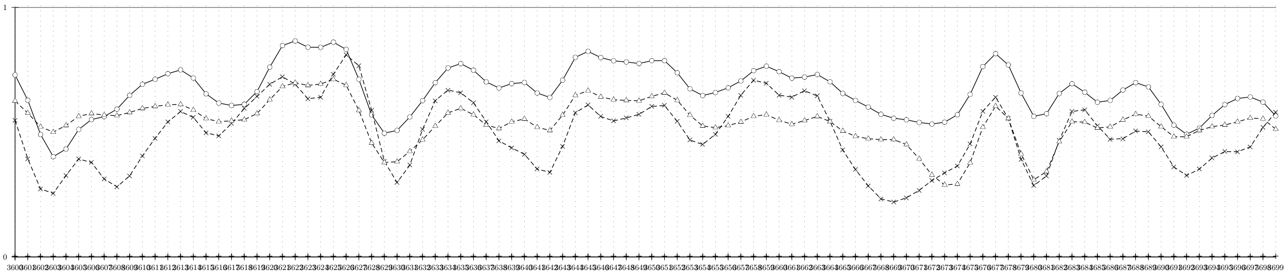

human	TCTCCTCCTAAATCGTCCCGGCAGTCACGTTCCAGGGGCAGTCCAGATCTCCCATCCTGGGAGGGAGAAGAAGGGCTGGAGACCCAAACACCCTGAGTAC
chimp	TCTCCTCCTAAATCGTCCCGGCAGTCACATTCCAGGGGCAGTCCAGATCTCCCATCCTGGGAGGGAGAAGAAGGGCTGGAGACCCAAACACCCTGGGTAC
colobus	--
baboon	TCTTCTCCTAAATGGCCCCGGCAGTCACGCTCCAGGGGCAGTTCAGATCTCCCACCCTGGGAAGGGGAAGAAGGACTGGAGACCCAAACGCCCTGAGTAC
macaque	TCTTCTCCTAAATGGCCCCGGCAGTCACGTTCCAGGGGCAGTTCAGATCTCCCACC-TGGGAGGGGGAAGAAGGACTGGAGACCCAAACGCCCTGAGTAC
dusky titi	--
owl monkey	--
marmoset	TCTTCTCCTAAATGGGGCCGG-AGTCACGTTCCTGGGGCAGTTCAGATCTCCCACCCTGGGAGGGAGGAAAAGTGCTGGAGACCCAACCACCCTGGGTAC
mouse lemur	--
galago	------CTTAAATGGCCCCAGCAGTCACGTTTTGGAGGGAGCATAGGTCCCCCACCCTGGGAAGGGGTAAGAAAGGTGGAGACCTGACCACACTGACCAC
rat	CC----------------------TCACCTGCAGCATGAAGCTCAGTTCCCCTGTCCCGGCTG--AGACAGAAGAGTTAGTCACCAGCCACTTAGGCTCC
mouse	CC----------------------TCACCCGTAGCACGAAGCTCAGTTCCCCTGCCCTGGCTG--AGGCAGAAGGGTTGGTCACCAACCACTTAGGCTAT
rabbit	CC----------------------TCACGTCCCGGAGGAAGCTCAG-ACCCCTGCCTCCTTCG----------GAGTGGACAGCTGACCACCTAGGCCAC
cow	CTGCTCGT-GAGTGGTCTCAGCAGCC-TGCCCTGGAGGGGACCCAGGTCT-CCATGCTGGGAGGGGGGGAAACGGTTG---------------CAG----
dog	CTTGTTGTTAAATGGTCCTGGCAATC---ATCTGGAGAGAGCTTAGGTCTTCCACCCTGGGAGGTGAGAGACAGGCTGGACACCCGACCACATCAG----
rfbat	CCTGCTCT-TAGTGGTCCTGACAGCCATGTTCTGGAGGCAG-GCAGGTCTCCCACCCTGGGAGGGAGGAGAAGGGCTGGAGCCCCGACCACACTGG----
hedgehog	--
shrew	--ATCACCTAACTAGGGAAGGGGGAGAAGGACTGGAAACCCAA-------------
armadillo	--CTAC
elephant	CT-TCTCCCAAACGGT-CTGGCAGTCACCTTCTGGAGGAAGTTCAG-----------------------GGCTGTCTGGTCCCCTAGATGTTCTAG----
tenrec	--
monodelphis	--
platypus	--
chicken	--
xenopus	--
tetraodon	--
fugu	--
zebrafish	--

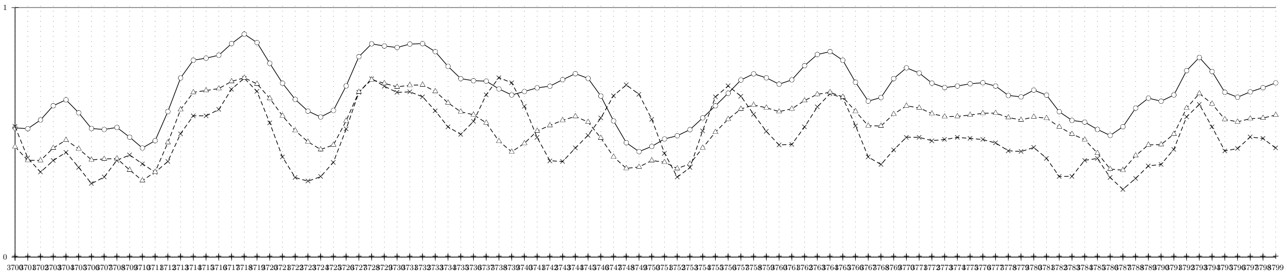

human	AGCACCCTGCTACTCCTCCTCAATAACTCCCAGAGTTCCGGCCAGGACATGGGACTGCTCCTCACCTCCAGACAGGCCAGGATTCCAGGGAATCTTACCG
chimp	AGCACCCTGCTACTCCTCCTCAATAACTCCCAGAGTTCCGGCCAGGACATGGGACTGCTCCTCACCTCCAGACAGGCCAGGATTCCAGGGAATCTTACTG
colobus	--
baboon	AGTACCCTGCTACTCCTCCTCAATAGCTCCCAGAGCTCCGGCCAGTCCACAGGACTGCTCCTCACCTCCAGACAGGCCAGGATTCCAGGGAATCTTACTG
macaque	AGTACCCTGCTACTCCTCCTCAACAGCTCCCAGAGCTCCGGCCAGTCCACGGGACTGCTCTTCACCTCCAGACAGGCCAGGATTCCAGGGAATCTTACTG
dusky titi	--
owl monkey	--
marmoset	CACACCCTGCTACTCCTCCTCAATAACTCCCAGAGTTCCGGCCA-------GGACTGCTCCTCACCTCGAGACAGGTCAGGATTCCAGGGAATCTCACTG
mouse lemur	--
galago	CACACCC-GACACTCCTCCTCAATAGTTCTCCGAGTTCCCACCAGGATGCGGGACCGCTCCCCACCTCCAGATGG----CGTTACCCAGGGATCTT----
rat	AGTACATGGGGATTCCCC--TAAGGACTCCC------------AGGACATAAGGGT-------------------------------ATGACTCTGGCTG
mouse	AGCACATGGGGACTCTCC--CAAGGACTCCCAGTGCTCTT-CTAGGACATAAGGGT-------------------------------AAGATTCTGGCTG
rabbit	GGCACCATGCGCCTCCTC--CTCCAACTCCCAGAGTTC-----TGAGCGTAACTGC-------------------------------GGGGATCTTGCTG
cow	-GCCCCATGCTCTTCCTC--CCACGAGTCTCAGGGCCCCAGCTGAGATGG-CAGCCACTCCTCACCCACAGACAGGGCTTGACCCCGGGGG-TCTCACTG
dog	-GCACCACGTCATTCCTC--CAACAACTCCCAGAGCTCCAGCAAGGAG-------CTCACCCCACCTGCAGACAGAGCTGGATTCTAGGGGGTCATACAG
rfbat	-ACACGCTGACATTCCTC--CAACAGCTC-CAGAGCTACAGCCAGGACAC-GGACCACTCGTCACCTGCAGGC----------TCTGTGGA-TCTCACCG
hedgehog	--
shrew	--
armadillo	GGTGCTC-ACCGCCC--------CACCTCCAAGAGGTCTG-CCAGGACGTGGCACCGCT---CACCTCCTGCCAGGGCATGACTCCGGTGCCTCTTCCCA
elephant	-GGCCCACACTCTTCTCCCTCAGCAGCTCCC-GAGCTCCGGTCAGGACACGGACCTGCCCCTCCACTTGAGACCCGGCATGATCTCACTGGCTCTTCCTG
tenrec	--
monodelphis	--
platypus	--
chicken	--
xenopus	--
tetraodon	--
fugu	--
zebrafish	--

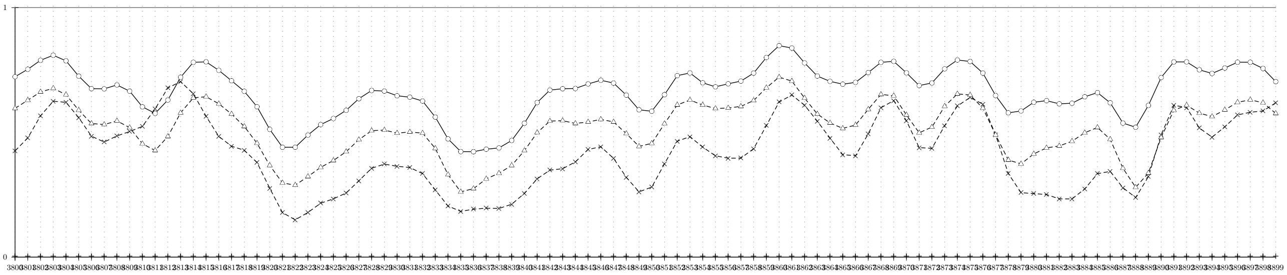

human	AGCAACTGCCATGTGCTGAGCTAAGTGCTGCCATTTATCATCTTAGTGAATCTCTTGATAAAATGCAAGGGGCCCTGGCAGATTAAATGATGCCAAGCCT
chimp	AGCAACTGCCATGTGCTGAGCTAAGTGCTGCCATTTATCATCTTAGTGAATCTCTTGATAAAATGCAAGGGGCCCT------------------------
colobus	--
baboon	AGCAACTGCCATGTGCTGAGCTAAGTGCTGCCATTGATCATCTTACTGAATCTCTTGATAAAACGCAAGGGGCCCTAGCAGATTGAATGATGCCAAGCCT
macaque	AGCAACTGCCATGTGCTGAGCTAAGTGCTGCCATTGATCATCTTACTGAATCTCTTGATAAAACGTAAGGGGCCCTAGCAGATTAAATGATGCCAAGCCT
dusky titi	--
owl monkey	--
marmoset	AGCAACTGCCATGTGCTGAGCTAAGTGCTGCCATTGATCATCTTACTGAATCTCTTGGTAAAATGCAAGGAGCCCTAACAGATTAAACAATCCCAAGCCT
mouse lemur	--
galago	AGTAACCGTCTCATGCTAAGCCAGGTACTGTCATTTATTGTCTCACTGACTCTCTTGATAAAACGCAAGTGGCCCTAACAGATTAAAGGATGCTCAGCCT
rat	A-CAACTGCCACATGCTACCATAGG--------------------------------ATAAAACACTAATGCTCCTAACAATGTA------GACAAGCTT
mouse	AGCGACTGCTATATGCTACCACAGG-ACAGTCA-TTACTATCTTTCTGAAGCTCTTAATAAAACACTAATGTTCCTAATAACT--------GGCAAGCCT
rabbit	AGCAACTGTCCCGTGCCAAGGCAGGGGCTGCCACTCACCAAGCTCCTGAACCTCCCGCCCAAATGCAAGTGGCCCTAATGGGTCACATGACGCCAAGCCT
cow	GACAACGACCCTCT--------CTGCGCTGCCATCCATGGTCTTGCGAAAA-----------AAGCTGGTGGCCCTAATGGATGAAACCATGCCAAGCCT
dog	AGCAATTGCCATGTGCTAAGTGTGGCGCTACTATTCCCTGTCTTACTGAAT--------AATATGCAAGTGCTCTCAACTAACTCAACTAAGCCCAGCCT
rfbat	AACAACCGCCATGTGCTAGGCCTAGGGCTGTGACTTACCACCTTACTGAGT--------AAAATGCAAGTGGCCATAAAAGATTAAATTATGCC-AGCCC
hedgehog	---AAAGTGTAAGTGATCCTAAAAGATAAAATGGTGCCAAGCTT
shrew	--
armadillo	GACAACCACCAAGCACTAAGCTCATTTTGGACACCGGGCGAATCACGGAGTCTCCGGACAAAATGCCAATGGCCCTAAGAGGTTAAACTCTGCCAAGCCT
elephant	AACACCTGCCATGTGACACGCACAATGGTGCCTTTTAC--TCTTACTAGTTAC--------------------------AGAATCCATTATGCCCGCACC
tenrec	--
monodelphis	---TGATGAA--GGGAATGTTCCG--CGGATTAAGTTTTGCTTATTCT
platypus	--
chicken	--
xenopus	--
tetraodon	--
fugu	--
zebrafish	--

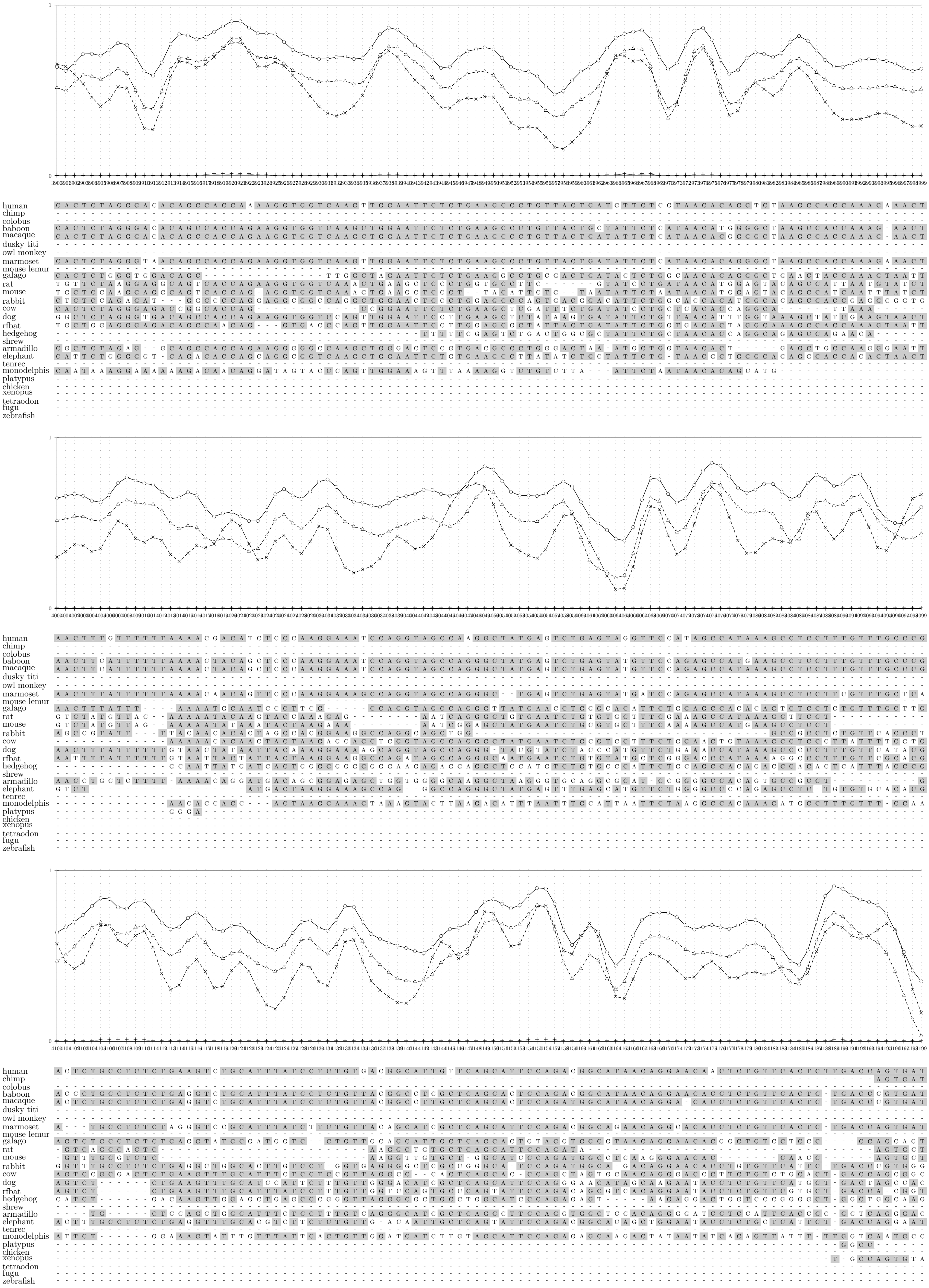
human
chimp
colobus
baboon
macaque
dusky titi
owl monkey
marmoset
mouse lemur
galago
rat
mouse
rabbit
cow
dog
rfbat
hedgehog
shrew
armadillo
elephant
tenrec
monodelphis
platypus
chicken
xenopus
tetraodon
fugu
zebrafish

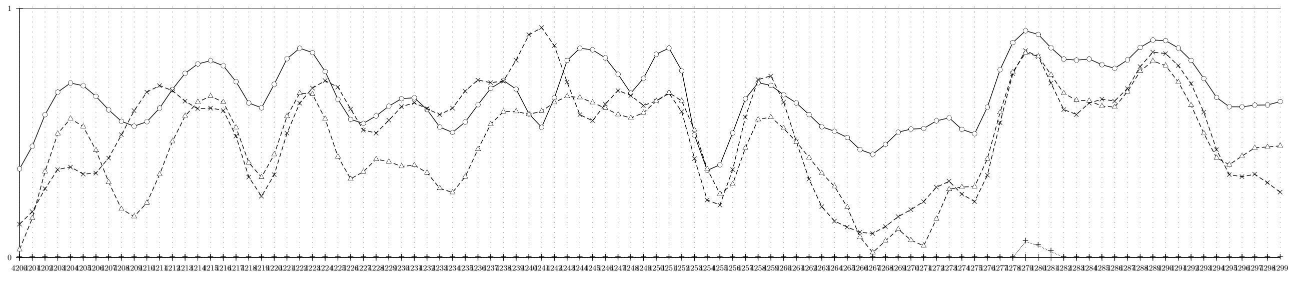

human	ATCAAAATGGTACTGACCAAGGCTTTGCACCCGCTCACCCACCTCCCTGCCCCACATCCTTCAATCTGGTGTACACAGCAACTGCAAAGGCTGCAGACTT
chimp	ATCAAAATGGTACTGACCACGGCTTTGCACCCGCTCGCCCGCCTCCCTGCCCCACACCCTTCAATCTGGTGTACACAGCAACTGCAAAGGCTGCAGACTT
colobus	--
baboon	ATTAAAATGGTACTGACCAAGGCTTTGCACCCGCTCACCCACCTCCCTGCCCCATACCCTTCAATCTGGTGTACAGAGCAACTGCAAAGGCTGCAGACCT
macaque	ATCAAAACGGTACTGACCAAGGCTTTGCACCCGCTCACCCATCTCCCTGCCCCATACCCTTCGATCTGGGGTACAGAGCAACTGCAAAGGCTGCAGACCT
dusky titi	--
owl monkey	--
marmoset	ATCAAAATGGTACTGACCAAAGCTTTGCACCCCCTCACCCGCCTTCCTG-CCCACACGCTTCAATCTGGTGTACAGAGCAGCTGCAAAGGCTGCAGACAT
mouse lemur	--
galago	ATCAAAACAACCCTGACAATAACTTCACCTCCGCGCACACA-----------CAAACCCTTCATCCCAGTGTACAGAGCAG-TGTAAAGGCTGCAGAC-T
rat	AGCAGCACACGACTGCTCACAGCCTTGC-CCCAAACACC-------------------------TGCTCTGAGCACTACAGCTA--GAGGCTGCAGGCGC
mouse	GTCAACACACAACTGCTCAAAGGCATCG-TCCGAGCAC--------------------------TGTAGCACGCACTGCAGCTACAGAGGCTGCAGCCTC
rabbit	GTCAAACCTGCACC-ACCGCTGCCTGGC-CACACGCACACGCGTTCTCA---------------GCCTGTGAA------------AGAGGCTGCGGCCTC
cow	TTTCAAGCCACGGTAACCAAAGTCTCTC-CACACACAC-------------------CCCTTATTCCTGTGTGCAAAGCAGCCACAGAGGCGGCTGACCT
dog	CTCCCAACCGCACTGACCGAAGCTTCAC-CGCATACGC-----------------GTCCTTCATTCTAGCGTGCAGAGCAGCTGCAAAAGCTGCAGACCT
rfbat	ATCCAAACCACACTGACCAAAGCTTCGC-CACCCACAC-----------------ATCCTTCAGTCTAGTGCACTCGGCAGCAGCAAAGGCTGCGGACTT
hedgehog	CGGCAGAGCGCACTGACCGGGGCTTCCT-C-----CGC-----------------CTCC----------TGAGCTCAGCAGCTGCACAGGCCACGGGCTC
shrew	--
armadillo	GGGAAAACCGTGCTGGCTAAAGCTTTGC-CACACCTA------------------GCTCTTTGAGGCAATGCACCGGGCAGCTGCACAGGCTGCAAACTG
elephant	CTCAAATCCACGCTGACCAGAGC-TCGC-CACACTGGC-----------------GGCCTGCATTCTGACAAACTCGGCAGCTGCAAAGGCTGTGAGTTT
tenrec	--
monodelphis	ATTAAAATAACA------AAAGCTTCAG-TGCATACTT-----------------TCCCCCTATTCTTTTATAAATACTAGTTG-GTAACCTATTTTCTT
platypus	-----GACCGTCACCACCCCGGCTTCCG-CCCGACGGTCCGCGTTGCCCGTCCCTCGCCGGCGGGACCGGGGGCCCGGCAGCCCCGGAGGCCGATGGCCC
chicken	--
xenopus	TTTCCACTGGTG--AATAAAAGCTTTTT-ACAACCTGTGCTGCTTGCCCCTCAAAAGAGGCCATACCATTATAAACACCAGTGGCAGGTGTTGCTGTTTT
tetraodon	--
fugu	--
zebrafish	--

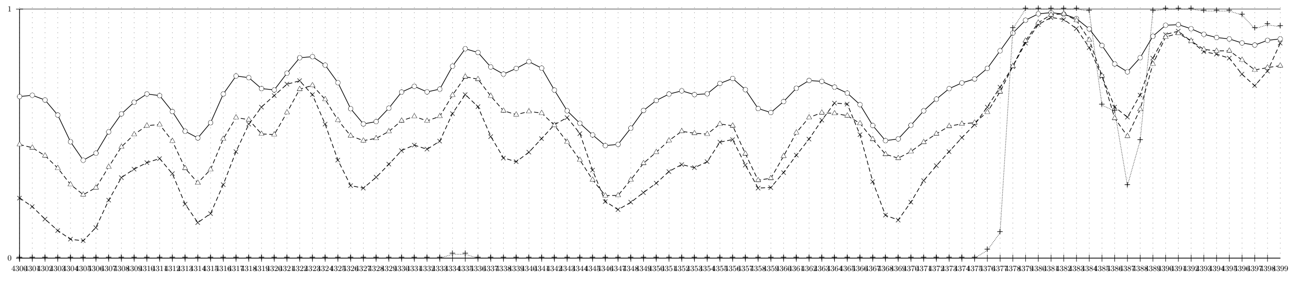

human	TTTTTGAACTTGGAAGAAACGGTTTGCAGTAGGTTTTTCAAAACAAATGGGCTTTCCTAACGGACAAGGGAGTACACCTACCTTTCACTTCCAACACCTG
chimp	TTTTTGTACTTGGAAGAAACGGTTTGCAGTGGGTTTTTCTTAACAAATGGGCTTTCCTAACGGACAAGGGAGTACACCTACCTTTCTCTTCCAACACCTG
colobus	--
baboon	TTTTCTAACTTGGACGAAACGGTTTGCAGCAGGGTTTTCAAAACAAATGGGCTTTCCTAAGGGACAAGGGGGTGCACCTACCTTTCACTTCCAACACCTG
macaque	TTTTCTAACTTGGAAGAAACGGTTTGCAGCAGGGTTTTCAAAACAAATGGGCTTTCCTAAGGGACAAGGGGGTGCACCTACCTTTCACTTCCAACACCTG
dusky titi	--
owl monkey	--
marmoset	TGTTCTAACTTGGAAGAAATGGTTTGCAGCAGGTTTTTCAAAACAAATGGGCTTTCCTAAGGGACAAAGGAGTACACCTACCTTTCACTTCCAACACTTG
mouse lemur	--
galago	TTTGCTAACCTGGAAGAAATGGTTTGCAGTAGGCTTTTAAAAACAAGTGAATTTTCCTAAGGACAAAAAAGGCACACCTACCTTTCAGTTCCAGCACCTG
rat	TTT-------TGGAAGAAACACT--------------------CGAGTAGGCTTCTC---------AGGGACCACTCCTACCTTTTATCTCCAGCACTTG
mouse	TTT-------TGGAAGAAACCCT--------------------TGCGGTAGGTTCCC---------------CACTCCCACCTTTTATCTCCAGGACTTG
rabbit	TGGTC--ACCCGGGAAGAACGGT--------------------CGAGCGGGCTTTC-TGAGGGACA------GACACGTACCTTTCAGCTCCAGCACCTG
cow	TTGTTAACC---CATTAAACG---------------TC--AGACAGGGAGGCTGGGCCCCAAGACA-----GCCCTCCTACCTTTCAACTCCAGCACTTG
dog	TTTTTATCCTTGCAGGAGACT--TTGCAGGAGACTTTCCAAAGCAATCAGGCTTTGCTATGAGACA-----GCACACCTACCTTTCAACTCCAGCACTTG
rfbat	TTTTTTGTG---GGGTAGAGT--GTGGGGTAGAGTTTT----ACAGACAGGCTTTGCTTCAAGACGAGACGGCACACCTACCTTTCACCTCCAGCACCTG
hedgehog	TGGATAAACGAAGCAAAGGCT---------------TTCGAAGTGACCGGCTTCCGCCGCCGGGCG---------GCCTACCTTTCACCTCCAGCACCTG
shrew	---TTCAGCTCCAGCACCTG
armadillo	CTTTTCAGC-TGAGAGGAACTGTTTATGGGAGGTTTTCCTAAACAAATAGGCTTGCCTAAGGGACA-AAGGGGACCCCTACCTTTCAACTCCAGCACTTG
elephant	TTT---AACCTGGGGAAAACAGTTTGTGTTAGATTTTTCAAATTGAGCAGGATGCCCCAAGGGATGAAGAGGAACACCCACCTTTCAACTCGAGCACTTG
tenrec	--
monodelphis	TTTTCTAGC-----TAAAGCTGTTCAAATTAAGTTTTCCAAAATTAATATCTTTCCCTCAGAAATA------GATGAATACCTTTCATTTCCAATACTCG
platypus	CGTCGGC---------------T---TACCTTTGAGCTCCAGCACCTG
chicken	--TAC---------------------TACCTTTTAATTCCAGCACTTG
xenopus	TCTTCTGTTTTTCCAGAA-----CTGTAACTAA---TGCAAAA-----------TAC------------------GCATACCATTCAGTTCTAGGATATG
tetraodon	-----------------------TTACTGTAAAAATAT-----------------CC-----------------A-CCTACCTTTGAATTCTAACACTTG
fugu	-----------------------TTATGGTCAAGATTT-----------------CT-----------------A-ATTACCTTTGAATTCTAAAACTTG
zebrafish	-----------------------GGGTTTTATACATTA-----------------AT---------------------TACCTTTAGTGTCCAGAATCTG

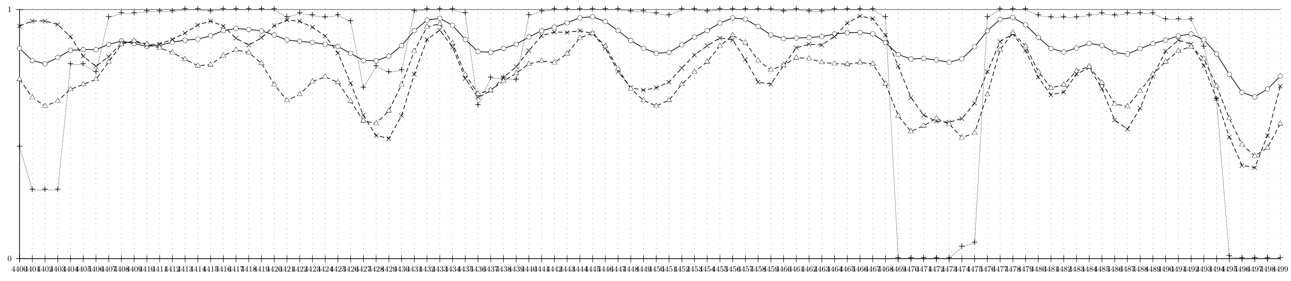

human	CACTATAGTGCTGTCATCACCCGCAATTAAAAAGGAGAGAGCAAACTGAACGTAGGCCTGCCGAACGTCGTACACTCCCTAGAGGACAAAGGAGACCTAC
chimp	CACTATAGTGCTGTCATCACCCGCCATTAAAAAGGAGAGAGCATACTCCCCCTCCGCCTGCCGAACGTCGTCCACTCCCTAGAAGACAAAGGAGACCTAC
colobus	--
baboon	CACTATAGTGCTGTCATCACCCGCAATTAGAAAGGAGAGAGCAAACTGAATGTAGGCCTGCCGAACGTCGTACACTCCCTGGAGGACAAAGGAGACCTAC
macaque	CACTATAGTGCTGTCATCACCCGCAATTAGAAAGGAGAGAGCAAACTGAATGTAGGCCTGCCGAACGTCGTACACTCCCTGGAGGACAAAGGAGACCTAC
dusky titi	--
owl monkey	--
marmoset	CACTATAGTGCTGTCATCACCCGCAATTAGAAAGGAGAGAGCAAACTGAACATAGGCCTGCCGGATGTCGTGCACTCCCTGGAGGAGAAAGGAGACCTCC
mouse lemur	--
galago	CACTATAGTGCTATCATCACCAGCAATTAAAAAGGAAAGGGCAAACTGAATGTAGGCCAGCCGGACATCATGCACTCCCTGGAGGACAGAGGAGACCCAC
rat	CCCTATAGTGTTGTCATCACCTGCAATGAGGAAGGAAAGGGCAAACTGGATATAGGCCAGCCGTACGTCATGCACTCCCTGTAGGACAAAGGAGACTCAC
mouse	TCCAATGGTGTTGTCATCACCCGCAATTAGGAAGGAAAGGGCAAACTGGATGTAGGCCAGCCGGACGTCATGCACTCCCTGTAGGACAAAGGAGACTCAC
rabbit	CGCTATAGTGCTGTCGTCACCAGCAATCAAGAAGGACAGAGCAAACTGGATGTAGGCCAGCCGGACGTCGGGCACTCCCTG-AGGACCAAAGAGAC-CAC
cow	CGTGATCGTGCTGTCATCACCGGCAATCAGAAAGGAAAGGGCGAACTGAATGTAGGCCAGCCGGACATCATGTACTCCCTGGGGG-CAAAGGAGAT-CGT
dog	CATGATCGTGCTGTCATCGCCGGCAATCAGAAAGGAAAGGGCAAACTGAATGTAGGCCAGCCGGACATCATGCACACCCTAGGGG--GAAGGAGAC-CAT
rfbat	CACTATGGTGCTGTCATCACCGGCAATCAGAAAGGAGAGGGCGAACTGGATGTAGGCCAGCCGGACGTCAGGCACGCCCTGGGGA-CAAAGGAGAC-CAC
hedgehog	CACGATGGTGCTGTCGTCGCCGGCGATGAGAAAGGCCAGGGCGAACTGGATGTAGGCCTGGCGGACGTCCTGTCCTCCCTGGGGGAGGAAGTAGAC-CTC
shrew	CACCACTGTGCTGGTGTCACCCGTGACAAGGAAGGAGAGCGCGAACTGGATATAGGCCTGCCGGACGTCGGGTGCCCCCTGGGGGATGAAGGAGAG-AGC
armadillo	CACGATGGTGCTGTCGTCACCTGCGATTAAAAAGGAAATGGCGAACTGGATGTAGGCCAGCCTGACGTCGGGCGCCCCCTGGAGGA-AAAGGAGACA---
elephant	CACGATGGTGCTGTCGTCACCGGCAATCAAGAAGGAAAGGGCAAACCGGATGTAGGCCAGCCGGACGTCGGGCACGCCCTGGAGG-GAGAGGAGGCCCAT
tenrec	--
monodelphis	TGCAATTGTATTATCATCACCAGCAATTAAGAAGGAAAGTGCAAACTGGATATATGCCAATCGAATATCTGGTAGCCCCTATTGGA-AAAGGAAACAGAT
platypus	CGCGATGGTGTTGTCGTCCCCGGCGATGAGGAAGGAGAGGGCGAACTGAACGTACGCCATCCGGACGTCGGGCAGGCCCTGGGGGACAACCGAGCGCT-C
chicken	TATCAGGACTGCAGTGTCACCAGCTATTAAAAAGGAGAGAGCAAACTGAATATAGGCCATACGGACATCAGGTCTTCCCTGTAAGAAAGATAAAATGCA-
xenopus	TATGATTGTAGGATTGTCCCCAGAAATGAAGAAAGACGTTGCAAACTGAATGTACGCCATTCTGACATCCGGCCTTCCCTAAAAAAAAAAAGAGAT--CA
tetraodon	TCCAATAGTTGTATTATCTCCAGACACCAAAAAAGACAGCACAAACTGAACAAAAGCCATGCGTACATCAGGTCTTCCCTGAGGAACAAA-GAAAA--A-
fugu	TCCAATGGTGGCATTATCTCCAGACACCAAAAAAGATAGCACAAACTGGACAAAAGCCATGCGTACATCAGGTCTTCCCTGAGTAACAAA-GAAAA--A-
zebrafish	GCCAATAGTTGTATTGTCTCCAGACATCAGGAAAGAAAGGACAAACTGAATGAAAGCCATTCTAACATCTGACTTTCCCTGAAAAAAATA-AAATC--A-

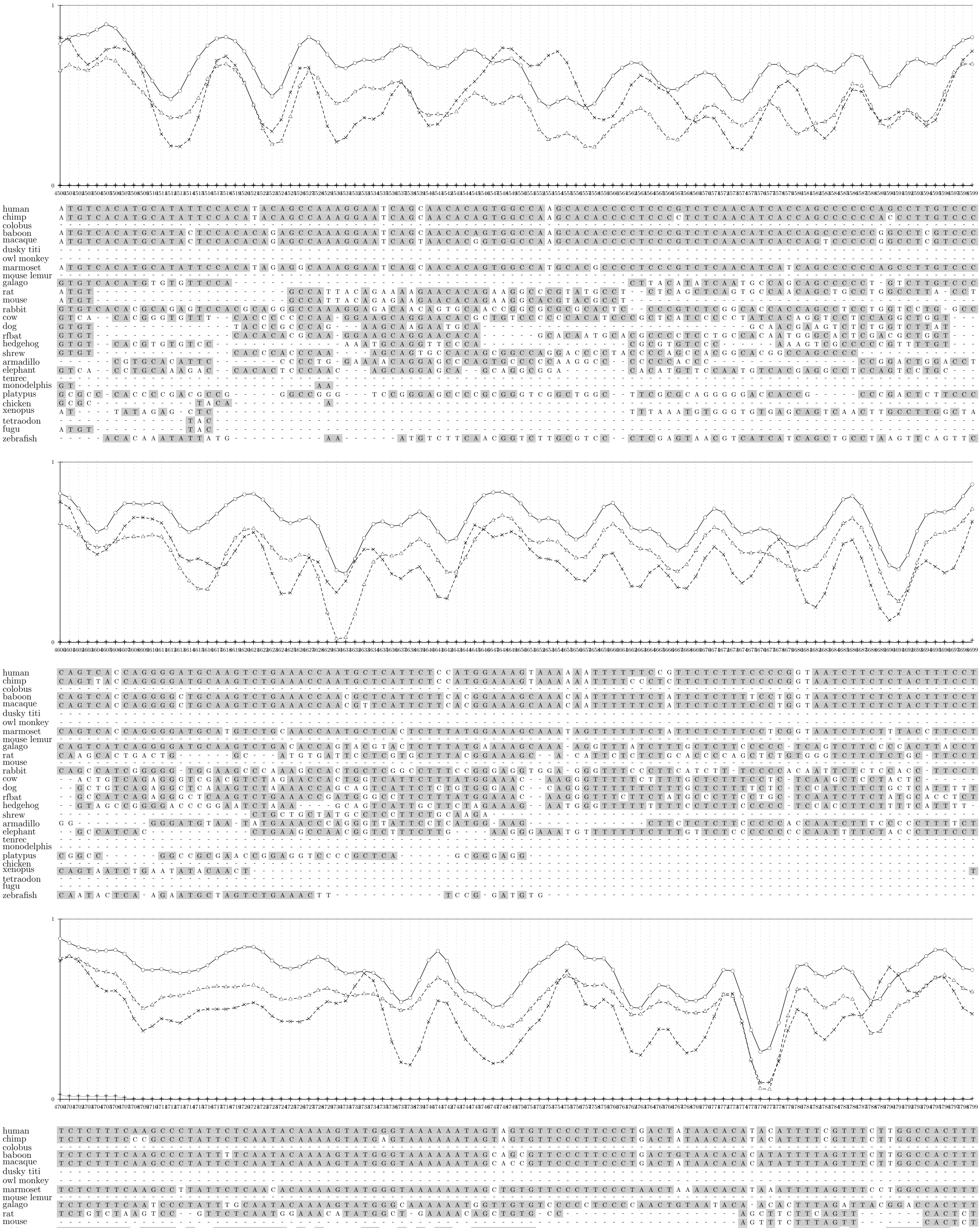

human
chimp
colobus
baboon
macaque
dusky titi
owl monkey
marmoset
mouse lemur
galago
rat
mouse
rabbit
cow
dog
rfbat
hedgehog
shrew
armadillo
elephant
tenrec
monodelphis
platypus
chicken
xenopus
tetraodon
fugu
zebrafish

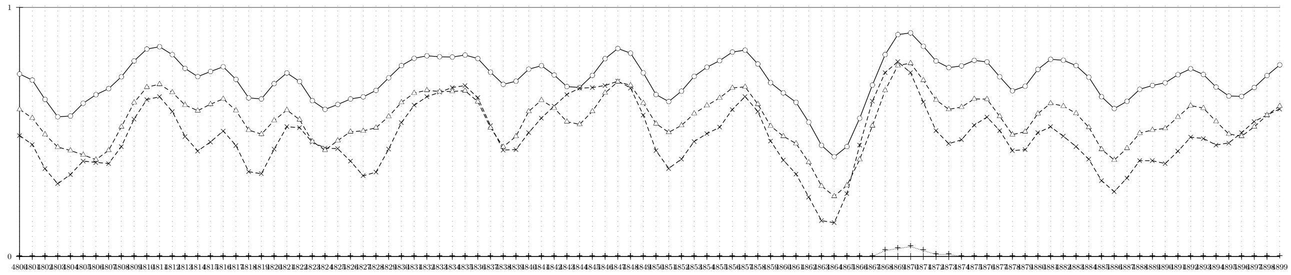

human	GGTGTAGAGCAAGACTCTCAGCCTGCCTAGATCCATCTCCTCTCGCTTGCTGATGAAAATAATCTATGTTTTTGAAAATCCAGAGATGATGAAATGGCTG
chimp	GGTGTAGAGCAAGACTCTCAGCCTGCCTAGATCCATCTCCTCTTGCTTGCTGATGAAAATAATCT--GTTTTTGAAAATCCAGAGATGATGAAATGGCTG
colobus	--
baboon	GGTGCAGAACAAGACTCTCAGCCTGCCTAGATCCATCTCCTCTCGCTTGCTAATGAAAATCATCTATGTTTTTGAAAATTCAGAGATGATGAAATGACTG
macaque	GGTGCAGAACAAGACTCTCAGCCTGCCTAGATCCATCTCCTCTCGCTTGCTAATGAAAATCATCTATGTTTTTGAAAAGTCAGAGATGATGAAATGACTG
dusky titi	--
owl monkey	--
marmoset	GGTGTACAGCAAGACTCTCAGCCTGCCTAGATCCATCTCCTCCCACTTGCTGATGAAAATAACCTATGTTTGTGAAAATCCAGAGATGATGAAATGACTG
mouse lemur	--
galago	GGCATACACCAAGATTCTCAGCCATCCAATATCCATCTTCTTTTGCTTGCTGAAGAAAAAAAATC-TGTTTTTGAAAACCCAGAGGTAATGCAAGGACTG
rat	GGGACAGGGCAAGAACCTTAGCCTGCTGGAATCCATCCCCTCTTGCTTGCTAATGCAAACAATCTATGTTTTAGAAAAGCCAGAGATAATAGAGACA-CG
mouse	AGGATAGGGAAACATCCTTTGCCTGCTGGGATCAATCCCCT-TTGCTTGCTAATGAAAACAATTATTGTTTTAGGAAAGCCAGAGATAATGGAGACA-TG
rabbit	GGTGCAGGACAAGAC--------TGCTTGGACCCATCTCCTCTCACTTGCCGATGGGAAGCATC--TGTTTTTGAAAATCCAGGGAGAAGGGAGGCG-TT
cow	GGTGTATAGCAAGATGCTTGGCCTGTCTACACCCATCTGCTCTCATGTC---ATGAAAATAGTCTCTGTTTTCGAAAATCCATAGATGATGGAAAGGCTG
dog	AGCATAGAGCAAGATTCTCAGCCCATCTAGATCCATCTATT-TCACTTCCCAGTGAAAATA-TACACTTTTTTGAAAATCCAGAGCCAATGGAAAGACCG
rfbat	GGTATAGAGCAAGATTCTCAGTC-GTCTAGATCCATCTATTCTCACTTGCCAATGAAAATAATCTACGTTTTTGAAAATCCAGAGGTAA-AGAAAGACTG
hedgehog	GCTGCATGGCGGGGTTCCTGGCCCACACAGACCCGCCTCCTCTCTCTTG-CAGTGAAAGGAGTGCTTTGTTTGGAAAAGCCTGTGAGGACGGACAGGCCG
shrew	--
armadillo	GGTACAGAGCAAGATTCTTGGTCTTCCTTGATCTGTTTATTCTCACCTGTTGATAAAAATAATCTTTTTTTTT----ATACAGAGAAAATGGAAGGACTG
elephant	GGTACAGAGCAAGGTTCTCAACCCACCTAGATCCATCTACTTTCACTTGCCAACGGAAATAACTTTTTTTTTTTTTAACTCAGAGAAAATGAAA-GGCTG
tenrec	---ACTCTCTTTTTTTGTGGCTGATTGCTAGATAAA-GCCTA
monodelphis	--
platypus	--
chicken	--
xenopus	--
tetraodon	--
fugu	--
zebrafish	--

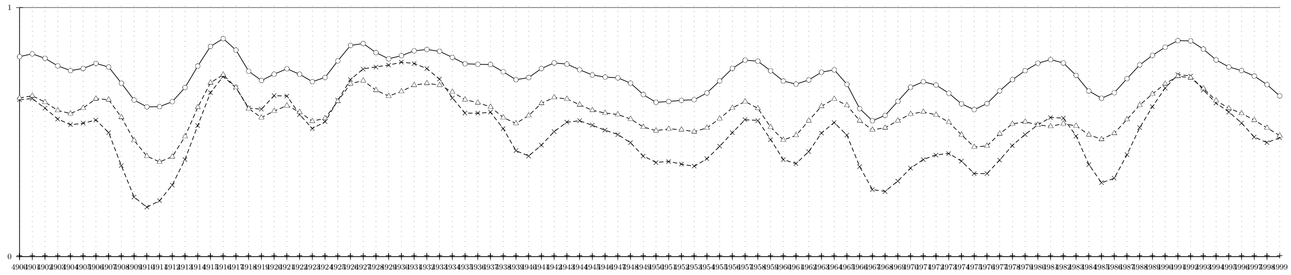

human	GTACTGCTTTTGGTGAGACACCAATGTGGTGATGTCTGGTTTTTATGAGATATTATAAATAACTTATTAGCTCATACCACAAACCAAAGCACAGATGCTG
chimp	GTACTGCTTTTGGTGAGACATCAATGTGGTGATGTCTGGTTTTTATGAGATATTATAAATAACTTATTAGCTCATACCACAAACCAAAGCACAGATGCTG
colobus	--
baboon	GTACTGCTTTGGGTGAGACATCAATGTGGCGATGTCTGGTTTTTATTAGATACTATAAATAACTTATTAGCTCATACCACAAACCAAAGCACAGATGCCG
macaque	GTACTGCTTTTGGTGAGACATCAATGTGGCGATGTCTGGTTTTTATTAGATACTATAAATAACTTATTAGCTCATACCACAAACCAAAGCACAGATGCTG
dusky titi	--
owl monkey	--
marmoset	GTACTGCTTTCAGTGAGACATCAACGTGGTGATGTCTGTTTTTTACTGGATATTATAAATAACGTATCAGCTTGTACCACAAACCAAAGCACAGATGCTG
mouse lemur	--
galago	GTCCTGCTTTTGATTAGACACCGATGTGGTGATGTCTGGTTTTCATTAGGTATTATAAAGAAGTTACTAGCTCATACCAGAAACCACAGCATAAAGGCTG
rat	GTGCTGCTTTTGATGAGAGGCCAATGTGGTGATATCTGGTTTTTATTAGGCATAGTAAATAAGTTACTACATCACACTGAAAACAAAAGTGCAGATGATG
mouse	GTGCTGCTTCTGATGAGAGGCCAATGTGGTGAGATCTGGTT---ATTAGAAATTGTAAATCAGTTATTA----ACACTGAAAAGCAAAGGACAGATGATG
rabbit	GTGCTGCTCCTGCTGAGACACCA--GTGGGGACGTGCAGCCTGTGTTGGACATCAGAAATCACTCATCA-CTCACCCCAGAGACCAGGGCACAGATGCTG
cow	GTGCTGCTTTTGATGAGACACCCATGAGGTGATGTTTGGTTTTTATTAGATGTTAGACAT-----------TCACAGCAGACA------CACAGATGAGG
dog	GTTCTACTTTAGTTGA--CACCAGTGAGGTGATGTCTGGGTTATATTAGGTACTATAAAT-----------GCACTCTGGACATCAAAGCACAGATGCTG
rfbat	GTGCTGCTTTTGATGAGATGCCAATGAGCTGATGTCTGGTTTTTACTAGATATTATAAAT-----------TTACGCCAGACACGGAAGCACAGTTGCTG
hedgehog	CTGCGGTTCTGGATGAGACACCAGCGAGGTGGTGTCTGGGTTTTATTGGGTGTCATGAGT-----------TCACCACACACACACACACACACACACAC
shrew	--
armadillo	GTGCTACTTTTGATGAGATGCCGAAG-GATGATGTCTGCTTTTTATTGGATATTGTCAATAAGTTACCAA-TCATAACAGAAACC-CAGCACAGATGCTG
elephant	GTGCCACTCTTGGCAG------AATGAGACAATGTCCGGTTTTTATTGGACATCATAAACAGGTTATCCACTTGTACCAGACACCAAAGCACAGACGCTG
tenrec	ATGTGAGTCATTTAAG------AACTAGACGATGCTTGGCCCTTTCTG--
monodelphis	--
platypus	--
chicken	--
xenopus	--
tetraodon	--
fugu	--
zebrafish	--

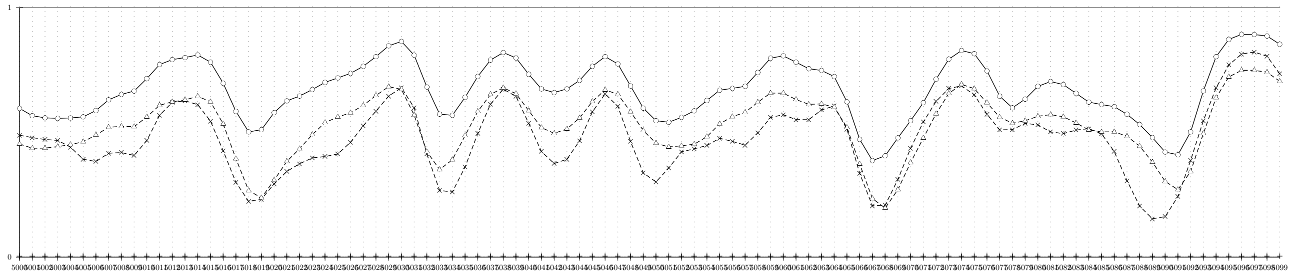

human	TCACGTTACTAACCCTGTTAGGCCTTCCCAGGGTTGTAGCCAGCACTGTCCACATGCATCAGCCACCAACCAGGGCACTGGGCACGTTTGGGGAGGGCCC
chimp	TCACGTTACTAACCCTGTTAGGCCTTCCCAGGGTTGTAGCCAGCACTGTCCACATGCATCAGCCACCAACCAGGGCACTGGGCACGTTTGGGGAGGGCCC
colobus	--
baboon	TCACGTTACCAACCCTGTTAGGCCTTCCCAGGGTTGCAGCCAGCACTGTCCACAGGCATCAGCCACCACCTAGGGCAGTGGGCACGTTTGGGGAGGGCCC
macaque	TCACGTTACCAACCCTGTTAGGCCTTCCCAGGGTTGCAGCCAGCACTGTCCACAGGCATCAGCCACCACCTAGGGCAGTGGGCACGTTTGGGGAGGGCCC
dusky titi	--
owl monkey	--
marmoset	TCACGTCACCAACCCTGTTAGGGCTTCCCTGGGTTGTAGCCAG---------CAGGCATCAGTCA-CACCCAGGGCAGTGGGCACACCTGCAGAGGGTCC
mouse lemur	--
galago	TCATGTCAC-AAACCTACAAGATCTACCTAGGGTTGTAGCCCTCACCGTCCACACGCATCAGACA-AGGCTTGGGCAGCAGGTACCCTGAAGGAGGG-CC
rat	CTATGTTACAAAG---GCATGGACATCCCAGGTC-ACAGCTTGCACTGGCTG------------ACCATGGGAGGC------------------GGGCAC
mouse	CTATGTTACGAAC---ACACGGGCATCT-GGGAT-ACAGCTCGCATTGCTTG------------ACCCAGGGAGGC------------------AGGCAC
rabbit	CGGGGCTGCAAACACTGCAGAGCCTTCCCAGGCTGGCAGCCTGCGCTGCCTGCGGGCTTCAGCCACCGGACGGGGC-----GCACCCCTGGCCAGGGCCC
cow	CCACATTACACACGCAGCATGGCCTTCCCAGGACTGCAGTCTGCACTGTCCGCGGGTGTCAGCTG-GACCCAGGAC----AGCATCCTCAACCAGGGCCC
dog	TCACATTACAAAC-CAGTGTGGCTTTTCCAGGA--------------------GAGTG----------------------GACATCCTCAAGGAGGGCCC
rfbat	TCATATTATAAACCCAGCGTGGCCTTCCCAGGATTGCAATCTGTACTGTCTACGGGCGTCAGCCA----------------------TCAGCCAAGGCCC
hedgehog	ACACACCACACACCCAGAGCGCTCTTCCCAGGACTGCAGTCTGCTCTGTCCACCAGTGTCTGTC-----CCACGACAGTGGGTGGC--CTGATGGGGCCC
shrew	--
armadillo	TTCTCTGACAAACCCAGCTTGGCCATCACAGGACGGCA-CCCGCCCTGCCCCTGGGCACCGGCCATGACCCAGGGCAGCGGGCAACCTCAGGGAGGGCCC
elephant	CTATACCATGAACCCAGCTCAGCCACGCCAGGATCCCGGCCTATACTGTTTGCAGGCCTCAGCCACAGCCCGGGACCACAGACATCCCCAGCGAGGACCC
tenrec	--
monodelphis	--
platypus	--
chicken	--
xenopus	--
tetraodon	--
fugu	--
zebrafish	--
